# Time-coexpress: temporal trajectory modeling of dynamic gene co-expression patterns using single-cell transcriptomics data

**DOI:** 10.1186/s12859-025-06218-w

**Published:** 2025-07-29

**Authors:** Shuyi Yang, Anderson Bussing, Giampiero Marra, Michelle L. Brinkmeier, Sally A. Camper, Shannon W. Davis, Yen-Yi Ho

**Affiliations:** 1 Department of Statistics, University of South Carolina, Columbia, USA; 2 Department of Statistical Science, University College London, London, UK; 3 Department of Human Genetics, University of Michigan, Ann Arbor, USA; 4 Department of Biological Sciences, University of South Carolina, Columbia, USA

**Keywords:** Zero-inflated bivariate count data, Single-cell RNA sequencing, Dynamic correlation, Pseudotime, Non-linear regression, Semiparametric regression, Covariate-dependent correlation structure

## Abstract

**Background:**

The rapid advancement of single-cell RNA sequencing (scRNAseq) technology provides high-resolution views of transcriptomic activity within individual cells. Most routine analyses of scRNAseq data focus on individual genes; however, the one-gene-at-a-time analysis is likely to miss meaningful genetic interactions. Gene co-expression analysis addresses this limitation by identifying coordinated changes in gene expression in response to cellular conditions, such as developmental or temporal trajectories. Existing approaches to gene co-expression analysis often assume restrictive linear relationships. However, gene co-expression can change in complex, non-linear ways, which suggests the need for more flexible and accurate methods.

**Results:**

We propose a copula-based framework, TIME-CoExpress, with proper data-driven smoothing functions to model non-linear changes in gene co-expression along cellular temporal trajectories. Our method provides the flexibility to incorporate characteristics commonly observed in scRNAseq data, such as over-dispersion and zero-inflation, into the modeling framework. In addition to modeling gene co-expression, TIME-CoExpress captures dynamic changes in gene-level zero-inflation rates and mean expression levels, providing a more comprehensive analysis of scRNAseq data. Through a series of simulation analyses, we evaluated the performance of the proposed approach. We further demonstrated its implementation using a scRNAseq dataset and identified differentially co-expressed gene pairs along the cellular temporal trajectory during pituitary embryonic development, comparing ${Nxn}^{-/-}$ and wild-type mice.

**Conclusions:**

The proposed framework enables flexible and robust identification of dynamic, non-linear changes in gene co-expression, zero-inflation rates, and mean expression levels along temporal trajectories in scRNAseq data. Detecting these changes provides deeper insights into the biological processes and offers a better understanding of gene regulation throughout cellular development.

## Introduction

Most existing tools for studying transcriptional activities using scRNAseq data focus on individual genes. While informative, this one-gene-at-a-time approach ignores interactions between genes. Arguably, changes in genetic interactions, sometimes occurring without alteration in mean expression levels, have been reported to play a critical role in downstream cellular transitions [1]. Gene co-expression analysis addresses this issue by identifying coordinated gene expression changes in response to the biological conditions in question. Current approaches to gene co-expression analysis, such as WGCNA [2], graphical LASSO [3], MIST [4], and CellTrek [5], assume static gene correlations and do not seek to capture the dynamic changes in genetic interactions in living cells within one unified modeling framework.

At the cellular level, two or more genes can have coordinated expressions that vary dynamically as cells progress through transitional states. For example, genes associated with pluripotency may show high co-expression early in embryogenesis, and the co-expression patterns of these genes may change over time as cells differentiate into specific lineages (such as ectoderm, mesoderm, or endoderm). Similarly, during immune cell activation, genes related to the immune response may show changing co-expression patterns along the temporal trajectory as cells transition from an inactive to an activated state. In neuronal differentiation, genes associated with neural progenitor identity may initially exhibit high co-expression, which alters as cells differentiate into distinct neuronal subtypes. Capturing these dynamic co-expression patterns along the cell temporal trajectory is critical for understanding how transcriptomic activity changes throughout cellular development.

Pseudotime is frequently employed in scRNAseq analysis to identify cells at various transitional states within a population. Pseudotime inference was first proposed by Trapnell et al. [6] to order cells along lineages by sequencing cells according to the gradual changes in gene expression rather than measuring real time. Since then, many studies, such as TSCAN [7], Slingshot [8], and Monocle [6], have validated pseudotime inference as a reliable method by comparing inferred cell pseudotime to known biological timelines. Experimental techniques such as lineage tracing have also been used as a validation approach. In this paper, we applied Slingshot [8] pseudotime analysis on scRNAseq data to reconstruct cellular temporal trajectories. This method is chosen based on the characteristics of our dataset. Slingshot is an unsupervised method that does not require predefined clusters. It is robust to noise in the data by estimating smoothed trajectories and allows the inference of multiple lineages. Other pseudotime inference methods can also be easily adapted into our proposed framework.

Several statistical methods have emerged to explore gene expression alterations along cellular temporal trajectories. Most existing analyses focus on one gene at a time. For instance, Van den Berge et al. [9] introduced tradeSeq, a generalized additive model framework based on the negative binomial distribution, which models non-linear changes in gene expression along pseudotime using zero-inflated data. However, this approach of examining genes individually may overlook important genetic interactions. Trapnell et al. [6] applied Monocle to trace single-cell gene expression trajectories over time, but it also ignores gene-gene interactions.

In biological systems, gene expression patterns often exhibit on-off behavior: some genes may turn on and then turn off over time, while others transition from inactive to active states. This biological on-off phenomenon can be observed through zero-inflation proportions of genes in scRNAseq data [10]. These zero-inflation rates often change as cells progress along pseudotime. To appropriately model these covariate-dependent zero-inflation, it is important to incorporate time-dependent zero-inflation proportions into the analysis. However, current researches on scRNAseq data analysis often fail to capture the dynamic changes of gene zero-inflation along cellular temporal trajectories.

A few methods have been introduced in the literature to study gene co-expression patterns. Yang and Ho [11] proposed the zero-inflated negative binomial dynamic correlation model (ZENCO), which incorporates zero-inflation for scRNAseq data but assumes a parametric linear relationship between gene co-expression and covariates. However, in molecular biology, gene co-expression may relate to cellular time in a non-linear semiparametric or nonparametric manner. Lau et al. [12] employed a network-based approach that inferred ten discrete co-expression networks by smoothly sliding from early to late development stages using consecutive time points. However, this method only models gene co-expression through discrete networks, whereas co-expression can change continuously over time. Hans et al. [13] proposed a boosting-based framework to capture complex dependence structures between outcome variables, which can be applied to gene co-expression analysis. However, this method does not handle zero values in gene expression measurements. Given that scRNAseq data is count-based and typically exhibits zero-inflation, especially with zero-inflation rates varying over pseudotime, the boosting method may fail to adequately capture such dynamic changes. scHOT [1] uses Spearman’s correlation to capture gene interactions along cell developmental trajectories but does not model changes in zero-inflation rates over pseudotime. In addition, scHOT can not simultaneously analyze and compare data across multiple groups (e.g., wild-type versus mutant) in the same modeling framework. SCODE [14] uses ordinary differential equations (ODEs) to model the temporal dynamics of gene expression and infers how each gene’s expression changes over pseudotime as a linear combination of the current expression levels of other genes. However, it can not directly model the non-linear changes in gene co-expression over time. Therefore, recognizing and modeling non-linear gene co-expression patterns across varying pseudotime domains is crucial for advancing our understanding of gene interactions.

We propose a flexible copula-based framework, TIME-CoExpress, to model and predict non-linear gene pair co-expression changes along cell pseudotime. A unique feature of this framework is its ability to accommodate covariate-dependent dynamic changes in correlation along cellular temporal trajectories. It also models dynamic gene zero-inflation patterns throughout cellular temporal trajectories to capture the biological on-off characteristics of gene expression. The copula-based structure enables us to construct a joint model with flexible marginal distributions, allowing TIME-CoExpress to capture the non-linear dependency between genes and to explore how predictor variables, such as cell pseudotime, influence gene-gene interactions.

An important advantage of TIME-CoExpress is its capacity for multi-group analysis, whereas many existing methods, such as scHOT [1], are limited to analyzing each group separately, which can lead to low efficiencies. The multi-group analysis enables the simultaneous examination of different groups of data, such as mutant versus wild-type groups, offering direct comparisons of gene co-expression patterns and changes in zero-inflation rates across cellular pseudotime in a unified analytical framework.

To model the correlation structure in a semiparametric manner, we extend generalized additive models for location, scale, and shape (GAMLSS) [15] to include zero-inflation and construct an additive distributional regression framework. The proposed framework allows for the modeling of multiple parameters of a distribution function, rather than just one parameter as in the traditional GAM models. Within distributional copula regression, each parameter of the response distribution is linked to covariates through additive predictors. The model is fitted using splines, which accommodate non-linear changes in dependence structures along temporal trajectories derived from scRNAseq data. A trust region method [16] is employed to simultaneously estimate the predictor effects.

Through a series of simulation studies, we verified that the proposed framework can capture the non-linear relationships between cell pseudotime and gene pairs interactions. It can also provide dynamic gene zero-inflation patterns across cell pseudotime. Our proposed analytical framework achieves higher power compared to the Gaussian-model-based model Liquid Association proposed by Li [17], the non-model-based approach scHOT [1], and the Conditional Normal Model (CNM) [18]. Moreover, our model has a significant advantage in terms of computational time compared to scHOT.

We applied the proposed algorithm to a mouse pituitary gland embryological development scRNAseq dataset. Specifically, we identified differentially co-expressed gene pairs along cellular temporal trajectories between ${Nxn}^{-/-}$ mice and the wild-type group, and found several genes with zero-inflation patterns that align with known biological processes.

This paper is structured as follows. The Materials and methods section describes the dataset used in our analysis, provides a brief theoretical overview of the proposed framework, and presents a series of simulation studies to validate the framework. This section also includes a power analysis to compare TIME-CoExpress with existing approaches. The results of real data analysis using mice *Nxn* pituitary gland embryological scRNAseq data are included in the Experimental data analysis section. Finally, the thorough discussion and conclusion are provided in the Discussion and Conclusions sections.

## Materials and methods

### Experimental dataset

The scRNAseq dataset used in this analysis was derived from dissected tissue containing part of the hypothalamus and the whole pituitary gland from embryonic day of development day 14.5 mouse embryos (e14.5). The dataset includes two groups: the control group contains tissue from one ${Nxn}^{+/+}$ and one ${Nxn}^{+/-}$ embryo, and the mutant group contains tissue from two ${Nxn}^{-/-}$ embryos. ${Nxn}^{+/-}$ mice are phenotypically normal. A total of 19,625 cells were sequenced, and the mean reads per cell was 27,387. Sequencing reads were processed, and cells were clustered using Seurat 4.1.0. Clusters were identified using marker genes, and clusters for hypothalamic cells, pituitary posterior lobe cells, blood cells, vasculature cells, and mesenchymal cells were removed from the analysis. The data set was further refined to only include anterior lobe pituitary cells that represent the *Sox2* stem cells, the *Prop1* progenitor cells, and the *Pou1f1* progenitor cells. Experimental data demonstrate that ${Nxn}^{-/-}$ embryos have a defect that alters the differentiation of pituitary stem cells into progenitor cells and eventually hormone-producing cells. We narrowed our data set to focus on this critical transition and employed TIME-CoExpress to identify gene pairs with differential co-expression patterns between the two groups during this critical developmental window. See Martinez-Mayer et al. [19] for a more detailed description of the data collection procedure.

The transcription factors (TFs): *Sox2*, *Prop1*, and *Pou1f1* are essential for cellular differentiation and the proper development of the pituitary gland. Yoshida et al. [20] demonstrated that *Prop1* coexists in *Sox2*-expressing cells. Olson et al. [21] reported that *Prop1* activates *Pou1f1* expression. The embryological developmental pathway for the pituitary gland follows the progression from *Sox2* progenitor to *Prop1* progenitor and then to *Pou1f1* progenitor, as progenitor cells differentiate into hormone-secreting cell types [22, 23]. In the following analysis, we mainly focus on *Sox2* progenitor with 766 cells, *Prop1* progenitor with 1249 cells, and *Pou1f1* progenitor with 1813 cells. There are 2138 cells from the wild-type group and 1690 cells from the mutant group.

**Fig. 1:**
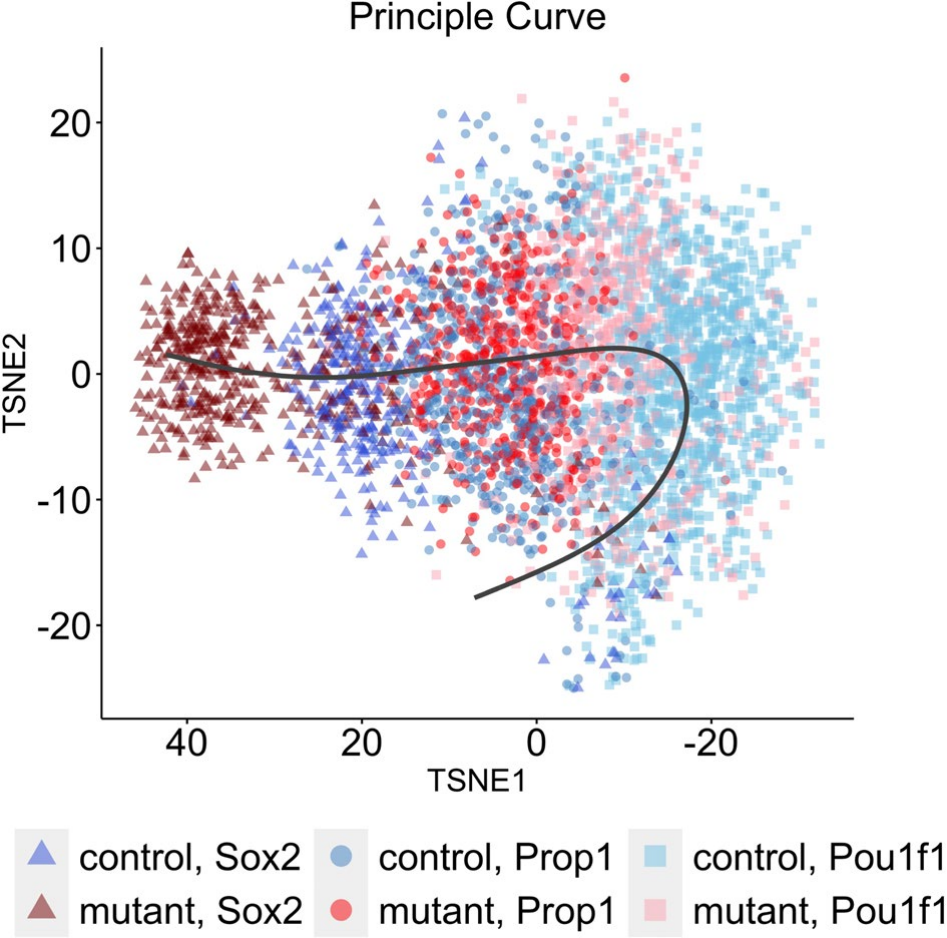
Cell Temporal Trajectory: The shades of red color points represent the mutant group, the shades of blue color represent the wild-type group; triangles, circles, and squares represent *Sox2* stem cells, *Prop1* progenitor cells, and *Pou1f1* progenitor cells, respectively. Each point represents one cell; the gray curve is the principal curve. The cell pseudotime increases along the principal curve (from left to right)

Figure 1 shows the cell trajectory estimated from Slinghot [8]. The shades of red color points are the mutant group, and the shades of blue color are the wild-type group. The triangles, circles, and squares represent *Sox2* stem cells, *Prop1* progenitor cells, and *Pou1f1* progenitor cells, respectively. There are more cells in the early cell stage (*Sox2* stem cells) and much fewer cells in the late stage (*Pou1f1* progenitor) in the mutant group compared to the wild-type group. This indicates that cells without the *Nxn* gene are unable to differentiate successfully and stall in an earlier stage of development. These findings are consistent with a known role for the WNT signaling component CTNNB1 (beta-catenin) interacting with *Prop1* to promote differentiation into *Pou1f1* progenitors and subsequent hormone cell types [21, 24]. NXN regulates the availability of Dishevelled (DVL) [25]; therefore, the stalled differentiation seen in ${Nxn}^{-/-}$ embryos is consistent with a defect in WNT mediated pituitary cell differentiation. The pseudotime analysis shows that more mutant ${Nxn}^{-/-}$ cells are in the *Sox2* stem cell and *Prop1* progenitor cell stage compared to the wild-type cells, suggesting that *Nxn* promotes the differentiation of pituitary stem cells. A similar findings of ${Nxn}^{-/-}$ embryos can be found in Brinkmeier et al. [26].

### Methods

In this section, we first introduce our zero-inflated copula model. We then describe the form of the additive predictors and the penalized likelihood used for parameter estimation. The model construction is discussed last.

#### Zero-inflated copula model

Let $\varvec{y_i}=(y_{i1}, y_{i2})^{\top }$ denote the gene expression measurements of a gene pair in cell *i*, where $i=1,...,N$ and *N* is the total number of cells. We assume that the two genes have zero-inflation rates $p_{i1}$ and $p_{i2}$, respectively. TIME-CoExpress implements a two-part model for the zero and non-zero expression values. For the non-zero expression values, denoted by $\varvec{w_i}=(w_{i1},w_{i2})^{\top }$, we assume Gamma (GA) distributions with parameters $\varvec{\vartheta _{i1} }=(\mu _{i1}, \sigma _{i1})^{\top }$ and $\varvec{\vartheta _{i2} }=(\mu _{i2}, \sigma _{i2})^{\top }$ for the two genes, respectively. We adopt the parametrization according to Rigby et al. [27]. The probability density function (PDF) and cumulative distribution function (CDF) are given by,

$$\begin{aligned} \begin{aligned}&f_{\text {GA}}(w;\mu,\sigma )=\frac{1}{\left( \mu \sigma ^2 \right) ^{\frac{1}{\sigma ^2}}\Gamma \left( \frac{1}{\sigma ^2} \right) }w^{\frac{1}{\sigma ^2}-1}\text {exp} \left\{ \frac{-w}{\mu \sigma ^2}\right\}, \end{aligned} \\ \begin{aligned}&F_{\text {GA}}(w;\mu,\sigma )=\frac{1}{\Gamma \left( \frac{1}{\sigma ^2}\right) }\gamma \left( \frac{1}{\sigma ^2},\frac{w}{\mu \sigma ^2} \right) \end{aligned}, \end{aligned}$$

where $w>0$, $\mu >0$ and $\sigma >0$. In this parametrization, $E(w)=\mu$ and $Var(w)=\sigma ^2\mu ^2$. We adopt the Gamma distribution for the marginals because the gene expression values in mouse pituitary gland scRNAseq data are normalized continuous measurements. Moreover, the Gamma distribution can account for the over-dispersion characteristic inherent in scRNAseq data. Alternative marginal distributions, such as the negative binomial (described in Supplementary Data–Section A.1), can also be implemented in the proposed framework.

The joint distribution of the non-zero values of the gene pair $\varvec{y_i}$ is modeled using a Gaussian copula. The Gaussian copula is widely used in genomic data analysis due to its flexibility and interpretability. It can capture the complex dependencies often observed in genomic data, with the dependence parameter modeled through splines. Additionally, its simple and intuitive mathematical structure, similar to the multivariate normal distribution, makes it easy to interpret when it comes to constructing joint distributions for different types of outcomes [28]. The PDF and CDF of the Gaussian copula are given by,

$$\begin{aligned} \begin{aligned} f_{W_{i1},W_{i2}}(w_{i1},w_{i2}; \varvec{\vartheta _i})&=c(F_{GA}(w_{i1}; \varvec{\vartheta }_{i1}),F_{GA}(w_{i2}; \varvec{\vartheta }_{i2}); \rho _{i})\\&\quad \cdot f_{GA}(w_{i1}; \varvec{\vartheta }_{i1}) f_{GA}(w_{i2}; \varvec{\vartheta }_{i2}), \end{aligned} \\ \begin{aligned} F_{W_{i1},W_{i2}}(w_{i1},w_{i2}; \varvec{\vartheta _i}))&=C(F_{\text {GA}}(w_{i1}; \varvec{\vartheta }_{i1}),F_{\text {GA}}(w_{i2}; \varvec{\vartheta }_{i2});\rho _i)\\&=\Phi _{2}(\Phi ^{-1}(F_{\text {GA}}(w_{i1}; \varvec{\vartheta }_{i1}),\Phi ^{-1}(F_{\text {GA}}(w_{i2}; \varvec{\vartheta }_{i2});\rho _i), \end{aligned} \end{aligned}$$

where $W_{i1}$ and $W_{i2}$ are the random variables representing the non-zero expression levels of two genes in cell *i*; $\varvec{\vartheta _i}=(\mu _{i1},\sigma _{i1},\mu _{i2},\sigma _{i2},\rho _i)^{\top }$ is the vector of all distributional parameters for cell *i*; $\rho _{i}$ is the dependence parameter that models the correlation between $w_{i1}$ and $w_{i2}$. $F_{GA}$ and $f_{GA}$ denote the CDF and PDF of the Gamma marginal distribution, respectively. $c\left( \cdot, \cdot; \rho _{i}\right)$ and $C\left( \cdot, \cdot; \rho _{i}\right)$ represent the PDF and CDF of the Gaussian copula, respectively. $\Phi _2(\cdot,\cdot; \rho _i)$ denotes the bivariate standard normal CDF with correlation $\rho _i$ and $\Phi ^{-1}(\cdot )$ is the quantile function of the standard univariate normal distribution.

For simplicity, we denote $F_{GA}(w_{i1}; \varvec{\vartheta }_{i1})$ and $F_{GA}(w_{i2}; \varvec{\vartheta }_{i2})$ by $u_{i1}$ and $u_{i2}$, respectively. The Gaussian copula density function $c\left( \cdot, \cdot; \rho _{i}\right)$ [29] is then given by:

$$\begin{aligned} \begin{aligned} c(u_{i1},u_{i2}; \rho _{i})&=\frac{\phi _2(\Phi ^{-1}(u_{i1})),\Phi ^{-1}(u_{i2}); \rho _{i})}{\phi (\Phi ^{-1}(u_{i1}))\cdot \phi (\Phi ^{-1}(u_{i2}))},\\&=\frac{1}{\sqrt{1-\rho _i^2}}\exp \left\{ -\frac{\rho _i^2\left[ \Phi ^{-1}\left( u_{i1}\right) ^2 + \Phi ^{-1}\left( u_{i2}\right) ^2\right] - 2\rho _i \Phi ^{-1}\left( u_{i1}\right) \Phi ^{-1}\left( u_{i2}\right) }{2(1-\rho _i^2)}\right\}, \end{aligned} \end{aligned}$$

where $\phi _2(\cdot,\cdot;\cdot )$ is the PDF of the bivariate normal distribution. $\Phi ^{-1}(u_{i1})$ and $\Phi ^{-1}(u_{i2})$ follow a bivariate normal distribution with correlation matrix $\varvec{\vartheta }_{i3}$:

$$\begin{aligned} \begin{aligned} (\Phi ^{-1}(u_{i1}), \Phi ^{-1}(u_{i2}))^{\top } \sim N_2 (\varvec{0},\varvec{\vartheta }_{i3}) =N_2 \begin{pmatrix} \begin{bmatrix} 0\\ 0 \end{bmatrix}, \begin{bmatrix} 1 & \rho _i \\ \rho _i & 1 \end{bmatrix} \end{pmatrix}. \end{aligned} \end{aligned}$$

To study zero-inflation patterns along the temporal trajectory, we incorporate this characteristic into the model. Define $D_{ij} \sim \text {Bern}\left( p_{ij}\right)$, for $j=1,2$, as a binary random variable indicating whether the expression measurement for gene *j* is zero in cell *i*. The random variables $Y_{i1}$ and $Y_{i2}$, representing the expression levels of two genes in cell *i*, are modeled as follows:

$$\begin{aligned} \begin{aligned} Y_{i1}&= (1-D_{i1})W_{i1},\\ Y_{i2}&= (1-D_{i2})W_{i2}. \end{aligned} \end{aligned}$$

The joint CDF of the expression levels of a gene pair can be expressed as:

1 $$\begin{aligned} \begin{aligned}&P\left( Y_{i1} \le y_{i1}, Y_{i2} \le y_{i2}\right) \\&= {\left\{ \begin{array}{ll} p_{i1}p_{i2} & \text {if } y_{i1}=0 \text { and } y_{i2}=0, \\ p_{i1}p_{i2} + F_{\text {GA}}\left( y_{i1}; \mu _{i1}, \sigma _{i1}\right) (1-p_{i1})p_{i2} & \text {if } y_{i1}>0 \text { and } y_{i2}=0, \\ p_{i1}p_{i2} + F_{\text {GA}}\left( y_{i2}; \mu _{i2}, \sigma _{i2}\right) p_{i1}(1-p_{i2}) & \text {if } y_{i1}=0 \text { and } y_{i2}>0, \\ p_{i1}p_{i2} + F_{W_{i1},W_{i2}}\left( y_{i1}, y_{i2}; \mu _{i1}, \mu _{i2}, \sigma _{i1}, \sigma _{i2}, \rho _i\right) (1-p_{i1})(1-p_{i2}) & \text {if } y_{i1}>0 \text { and } y_{i2}>0. \end{array}\right. } \end{aligned} \end{aligned}$$

The log-likelihood function is given by:

2 $$\begin{aligned} \begin{aligned} \ell _i&= \ell \left( \mu _{i1}, \mu _{i2}, \sigma _{i1}, \sigma _{i2}, \rho _i; y_{i1}, y_{i2} \right) \\&= c_i +{\left\{ \begin{array}{ll} 0 & \text {if } y_{i1}=0 \text { and } y_{i2}=0, \\ \log \left( f_{\text {GA}}\left( y_{i1}; \mu _{i1}, \sigma _{i1}\right) \right) & \text {if } y_{i1}>0 \text { and } y_{i2}=0, \\ \log \left( f_{\text {GA}}\left( y_{i2}; \mu _{i2}, \sigma _{i2}\right) \right) & \text {if } y_{i1}=0 \text { and } y_{i2}>0, \\ \log \left( f_{W_{i1}, W_{i2}}\left( y_{i1}, y_{i2}; \ \mu _{i1}, \mu _{i2}, \sigma _{i1}, \sigma _{i2}, \rho _i\right) \right) & \text {if } y_{i1}>0 \text { and } y_{i2}>0. \end{array}\right. } \end{aligned} \end{aligned}$$

where $c_i$ is an additive constant that depends on $p_{i1}$ and $p_{i2}$. The estimation of the parameters $\mu _{i1}, \mu _{i2}, \sigma _{i1}, \sigma _{i2}, \rho _i$ does not depend on the values of $p_{i1}$ and $p_{i2}$. Details on the derivation of Equation (1) and Equation (2) are provided in Supplementary Data–Section A.2. For *N* cells, the overall log-likelihood function is given by $\ell =\sum _{i=1}^N \ell _i$.

#### Additive predictor

Define the full parameter vector as $\varvec{\vartheta }=(\theta _1,...,\theta _M)^{\top }$, where *M* is the total number of parameters. In the real data analysis, we model a total of seven parameters: the marginal parameters of the Gamma distributions ($\mu _1$, $\sigma _1$, $\mu _2$ and $\sigma _2$), the copula dependence parameter capturing the correlation between the expression levels of the two genes ($\rho$), and the zero-inflation rates for each gene ($p_1$ and $p_2$). In this case, the full parameter vector is $\varvec{\vartheta }=(\mu _1,\sigma _1,\mu _2,\sigma _2, \rho, p_1, p_2)^{\top }$.

All parameters in the proposed framework can be modeled in a non-linear fashion related to covariates along the cellular temporal trajectory. Each parameter $\theta _m$ is linked to its corresponding additive predictor through a one-to-one, monotonic, and differentiable transformation function $g^{(\theta _m)}(\cdot )$, ensuring that parameter estimates remain within valid ranges. For the distributional parameters $\mu$ and $\sigma$, we use the $\text {log}()$ link function to ensure positivity. To constrain the correlation parameter $\rho$ to the interval $(-1,1)$, we use the inverse hyperbolic tangent function as its link. For the zero-inflation rate *p*, we use the $\text {logit}()$ link function to ensure that *p* lies within the interval (0, 1). For each cell *i*, the additive predictors are defined as:

$$\begin{aligned} \begin{aligned} \eta _{i}^{(\mu _j)} = \log ({\mu _{ij}}), \ \ \ \eta _i^{(\sigma _j)} = \log ({\sigma _{ij}}), \ \ \ \eta _i^{(\rho )} = \text {tanh}^{-1}({\rho _i}), \ \ \ \eta _i^{(p_{j})}=\text {logit}(p_{ij}), \end{aligned} \end{aligned}$$

where $j=1,2$ indexes the genes in the gene pair. The inverse link functions for the additive predictors are related to the model parameters as follows:

$$\begin{aligned} \begin{aligned} \mu _{ij} = \exp ({\eta _i^{(\mu _{j})}}), \ \ \ \sigma _{ij} = \exp ({\eta _i^{(\sigma _j)}}), \ \ \ \rho _i = \text {tanh}({\eta _i^{(\rho )}}), \ \ \ p_{ij}=\text {sigmoid}(\eta _i^{(p_{j})}). \end{aligned} \end{aligned}$$

Let $\varvec{z}_k=(z_{k1},...,z_{kN})^\top$ denote the vector of values for the *k*-th continuous covariate across all cells, where $z_{ki}$ is the value of the *k*-th covariate for cell *i*. In the mouse pituitary gland scRNAseq data analysis, we consider cell pseudotime as the covariate. Moreover, the model is flexible and can accommodate additional covariates.

TIME-CoExpress is flexible in modeling temporal effects across multiple categorical groups (e.g., mutant and wild-type). The group level is incorporated into the model via an indicator variable [30]. Let $g_i$ denote which group of cell *i* belongs to, and define $\mathbbm {1}(g_i=g)=1$ if cell *i* belongs to group *g* $(g\in \{1,\dots, G\})$, and 0 otherwise. In our application, we consider $G=2$ groups: mutant and wild-type. The additive predictor for $\theta _m$ consists of an intercept $\beta _1^{(\theta _m)}$ and group-specific smoothing functions $s_{k,g}^{(\theta _m)}(z_{ki})$, which capture the effect of the *k*-th covariate in group *g*. Each smoothing function is represented using splines, which are linear combinations of basis functions.

3 $$\begin{aligned} \begin{aligned} \eta _i^{(\theta _m)}&= \beta _1^{(\theta _m)} + \sum _{g=2}^{G} \mathbbm {1}(g_i = g) \beta _g^{(\theta _m)} + \sum _{g=1}^{G}\sum _{k=1}^{K}\mathbbm {1}(g_i = g) s_{k,g}^{(\theta _m)}(z_{ki}), \\&= \beta _1^{(\theta _m)} + \sum _{g=2}^{G} \mathbbm {1}(g_i = g) \beta _g^{(\theta _m)} + \sum _{g=1}^{G} \sum _{k=1}^{K} \sum _{j_k=1}^{J_k} \mathbbm {1}(g_i = g)\beta _{k,j_k,g}^{(\theta _m)} \, b_{k,j_k,g}^{(\theta _m)}(z_{ki}), \end{aligned} \end{aligned}$$

where $\beta _g^{(\theta _m)}$ is the coefficient for the fixed effect associated with the group *g*. The function $b_{k,j_k,g}^{(\theta _m)}(\cdot )$ denotes the $j_k$-th basis function of the spline for parameter $\theta _m$, corresponding to the *k*-th covariate in group *g*. ${J_k}$ is the total number of basis functions for the *k*-th covariate, and $\beta ^{(\theta _m)} _{k,j_k,g}$ is the group-specific basis coefficient. This structure enables the model to fit distinct smoothing functions for each group while sharing a common model framework. In the experimental data analysis, we set $K=1$, where $z_{1i}$ represents the cell pseudotime for the *i*-th cell. Common choices for splines include thin plate splines, Duchon splines, cubic regression splines, B-splines, P-splines, random effects, Markov random fields, Gaussian process smooths, Soap film smooths, and splines on the sphere.

Estimating zero-inflation rates $p_1$ and $p_2$ differs from estimating other parameters. The result from Equation (4) allows us to estimate $\varvec{\beta }^{(p _ 1)}$ and $\varvec{\beta }^{(p _ 2)}$ via logistic regression on $\varvec{1}\left\{ Y_{ij}=0\right\}$, while $\varvec{\beta }^{(\mu _1)}, \varvec{\beta }^{(\mu _2)}, \varvec{\beta }^{(\sigma _1)}, \varvec{\beta }^{(\sigma _2)}, \varvec{\beta }^{(\rho )}$ are estimated by maximizing $\ell =\sum _{i=1}^{n}\ell _i$. This maximization is achieved using the trust region algorithm, as detailed in Section Parameter estimation, which requires the gradient and Hessian to be explicitly calculated. The full model settings for both the simulation and experimental data analysis are provided in Supplementary Data–Section A.2.

4 $$\begin{aligned} \begin{aligned} p_{ij}&= P(D_{ij} = 1), \\&= P(D_{ij} =1 \left| Y_{ij}>0\right. )P(Y_{ij}>0) + P(D_{ij} =1 \left| Y_{ij}=0\right. )P(Y_{ij}=0), \\&= 0\cdot P(Y_{ij}>0) + 1\cdot P(Y_{ij}=0),\\&= P(Y_{ij}=0). \end{aligned} \end{aligned}$$

#### Penalization

To avoid overfitting, the model maximizes the penalized log-likelihood rather than the standard likelihood. It is defined as follows:

5 $$\begin{aligned} \ell _{p}(\varvec{\delta }) = \sum \ell _i - \frac{1}{2}\varvec{\delta }^{\top }\varvec{S}\varvec{\delta }. \end{aligned}$$

Define $\varvec{\delta }=(\varvec{\beta }^{(\mu _1)\top },\varvec{\beta }^{(\mu _2)\top },\varvec{\beta }^{(\sigma _1)\top },\varvec{\beta }^{(\sigma _2)\top },\varvec{\beta }^{(\rho )\top })^{\top }$ as a coefficient vector that combines all coefficients defined in Equation (3). The penalty matrix is defined as $\varvec{S}=\text {diag}\left( \varvec{D}^{(\mu _1)}, \varvec{D}^{(\mu _2)}, \varvec{D}^{(\sigma _1)}, \varvec{D}^{(\sigma _2)}, \varvec{D}^{(\rho )}\right)$, where each $\varvec{D}^{(\theta _m)}$ is a block-diagonal matrix constructed for the corresponding parameter. These matrices incorporate the smoothing parameters $\varvec{\lambda }^{(\theta _m)}$, which control the trade-off between model fit and smoothness. Each $\varvec{D}^{(\theta _m)}$ contains the individual penalty matrices $\varvec{D}_{k,g}^{(\theta _m)}$, corresponding to *k*-th smoothing term for group *g* in the additive predictor for parameter $\theta _m$. Specifically,

$$\begin{aligned} \varvec{D}^{(\theta _m)} = \textrm{diag}\left( \lambda _{1,1}^{(\theta _m)}\varvec{D}_{1,1}^{(\theta _m)}, \dots, \lambda _{K,1}^{(\theta _m)}\varvec{D}_{K,1}^{(\theta _m)},\dots, \lambda _{1,G}^{(\theta _m)}\varvec{D}_{1,G}^{(\theta _m)}, \dots, \lambda _{K,G}^{(\theta _m)}\varvec{D}_{K,G}^{(\theta _m)} \right), \end{aligned}$$

where $\lambda _{k,g}^{(\theta _m)}$ is the smoothing parameter associated with the *k*-th smoothing term for group *g* and parameter $\theta _m$.

As described previously, there are many choices for spline basis functions in Equation (3). In the following analysis, we use thin plate regression splines due to their flexibility and stable low-rank approximation, which avoids manual knot selection [31]. In this case, the penalty matrix $\varvec{D}^{(\theta _m)}_{k,g}$ is derived from the integrated squared second derivatives of the basis functions. The details of how $\varvec{D}^{(\theta _m)}_{k,g}$ is computed can be found in Marra et al. [32]. Penalty formulations for other splines can be found in Ruppert et al. [33] and Stasinopoulos [34].

Importantly, only the smoothing terms are penalized in the model. Fixed effect terms, including categorical covariates like group indicators, are not penalized. This is achieved by setting the corresponding rows and columns in the penalty matrix *S* to zero, which means no penalty is applied to the fixed effect coefficients.

#### Parameter estimation

We implement the extended trust region algorithm [32, 35] to maximize the penalized log-likelihood in Equation (5) for the estimations of the coefficient vector $\varvec{\delta }$ and the smoothing parameters $\varvec{\lambda }$. The estimation procedure is as follows,

1. Initialize the smoothing parameters $\lambda$s.
2. At iteration *a*, hold $\varvec{\lambda }^{[a]}$ as constants at their values from the last iteration or step 1 if it’s the first iteration. The trust region algorithm seeks to maximize $\ell _p$ in Equation (5). This is equivalent to finding a step to update the parameters, where the step is constrained by a trust region radius. The step is obtained by minimizing a quadratic approximation of $-\ell _p$ about $\varvec{\delta }^{[a]}$, which contains the penalized gradient and Hessian of Equation (5). The details about how to construct the quadratic approximation of $-\ell _p$ can be found in To evaluate whether [36]. To evaluate whether $\varvec{\delta }^{[a+1]}$ increases the penalized log-likelihood, the algorithm computes the ratio of the actual to predicted decrease in the objective function. If this ratio is small, the proposed update is rejected and the trust region radius is decreased. Otherwise, the update is accepted. The trust region radius is increased only when the ratio is sufficiently high and the step size equals the current trust region radius; otherwise, it remains unchanged. Details on the ratio calculation and radius adjustment can be found in Geyer [16].
3. Given $\varvec{\delta }^{[a+1]}$, the smoothing parameters $\varvec{\lambda }^{[a+1]}$ are updated by minimizing a smoothing parameter selection criterion that incorporates the current estimates of the penalized Hessian and gradient [36].
4. Repeat steps 2 and 3 until $\ell _p$ converges.

The trust region algorithm evaluates the objective function only after solving the trust region problem, rather than repeatedly estimating it. This makes it faster and more reliable than standard approaches such as Newton–Raphson [35, 37]. The algorithm requires both the gradient and the Hessian of the objective function; they are provided in Supplementary Data–Section A.3 and A.4. The smoothing parameter selection criterion used in step 3 follows Marra and Radice [36], which provides stable and efficient performance for copula models whose parameters depend on covariates, potentially in a non-linear manner.

Our framework uses thin plate regression splines. Other splines mentioned in Section Additive predictor can also be used. To get the estimated parameter vector $\hat{\varvec{\theta }}_{m}$, we insert the estimated additive predictors $\hat{\varvec{\eta }}^{(\theta _{m})}$ into the inverse link functions, $\hat{\varvec{\theta }}_m=g^{(\theta _m)^{-1}}\left( \hat{\varvec{\eta }}^{(\theta _{m})}\right)$. The detailed estimation procedure is described in Marra et al. [32], Radice et al. [35].

#### Model building

The mouse pituitary gland embryological development scRNAseq dataset contains two groups: wild-type and ${Nxn}^{-/-}$ mice data. We use a binary indicator $g_i$, taking values 1 and 2, to denote the wild-type and mutant groups, respectively, for cell *i*. TIME-CoExpress fits group-specific smoothing functions simultaneously for each parameter. To accommodate the two groups, we drop the subscript *k* in Equation (3), since only one continuous covariate, cell pseudotime $z_i$, is used. In this case, the additive predictor for $\theta _m$ is defined as,

6 $$\begin{aligned} \begin{aligned} \eta _i^{(\theta _{ m})}&=\beta _{1}^{(\theta _m)}+\beta _{2}^{(\theta _m)}\mathbbm {1}(g_i=2)+s_{g_i=1}^{(\theta _m)}(z_{i})\mathbbm {1}(g_i=1)+s_{g_i=2}^{(\theta _m)}(z_{i})\mathbbm {1}(g_i=2), \end{aligned} \end{aligned}$$

where $\beta _{1}^{(\theta _m)}$ is the intercept for the wild-type group, and $\beta _{2}^{(\theta _m)}$ represents the fixed effect capturing the difference between the wild-type and mutant groups. The smoothing functions $s_{g_i=1}^{(\theta _m)}(\cdot )$ and $s_{g_i=2}^{(\theta _m)}(\cdot )$ model the non-linear effect of pseudotime $z_i$ for wild-type and mutant groups, respectively. We implement the model in R using the formula $\texttt {eq}_{\theta _m}$ $\texttt {<}$- $\texttt {gene}_j$ $\sim$ group + s($\varvec{z}$, by=group), where z is the cell pseudotime. The variable $\texttt {gene}_j$ refers to the gene expression level: $\texttt {gene}_1$ is used to estimate the parameters $\mu _1$, $\sigma _1$, and $p_1$; $\texttt {gene}_2$ is used for $\mu _2$, $\sigma _2$, and $p_2$; and both gene expression levels are used to estimate $\rho$. The term $\sim$ group specifies that the model includes a fixed group effect, allowing different intercepts for each group. The function s() is the smoothing function that models the non-linear changes in parameters over pseudotime, and the number of basis functions can be specified within s(). The argument by = group in the smoothing terms ensures each group has its own smoothing curve over pseudotime.

In the experimental data analysis, we assume that the coefficient of variation (CV), $\sigma _1$ and $\sigma _2$, are modeled using a shared smoothing function s(z) across groups. We do not include a group-specific term because the relative dispersions appear biologically similar between the control and mutant groups in our dataset. Additionally, group expression differences are already captured through the modeling of $\mu _1$ and $\mu _2$; adding more spline terms would increase model complexity and risk overparameterization in this specific study.

We use quantile residuals to evaluate the goodness of fit for our model. For the non-zero continuous outcomes, the normalized quantile residuals are given by $\hat{r}_{ij}=(1-\hat{p}_{ij})\phi ^{-1}\{F_j(y_{ij}|{\hat{\mu }}_{ij},{\hat{\sigma }}_{ij})\}$, for $i=1,...,N$ cells, $j=1,2$, where $\hat{p}_{ij}$ is the predicted zero-inflation rate of gene *j* in cell *i* [36]. For the observed zero values, we sample uniform variables from the range $(0, \hat{p}_{ij}]$ as residuals [38].

### Simulation

This section presents three simulation studies conducted to evaluate the performance of the TIME-CoExpress framework, along with comparisons to existing methods. All smoothing functions in the section were estimated using thin plate regression splines.

#### Scenario I

**Fig. 2:**
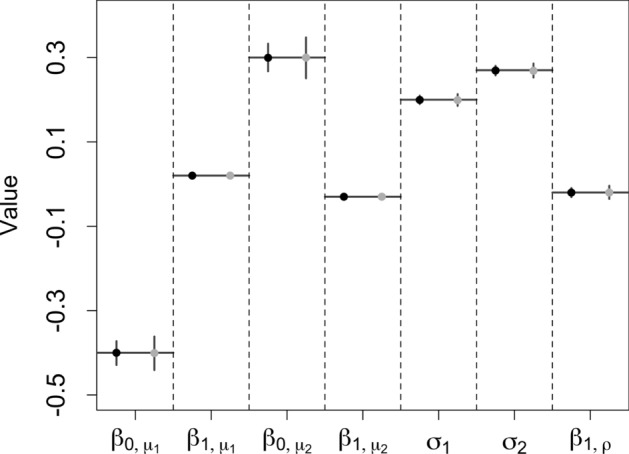
Scenario I simulation: The gray color is for data with 500 observations, and the black color is for data with 1000 observations. The horizontal lines are the true coefficients, the dots are the coefficient estimations. The vertical lines represent the 5–95% quantile

We first tested the proposed framework in a linear setting where the additive predictor has a linear relationship with cell pseudotime *z* as shown in Equation (7). Assume $\sigma _1=0.2$, and $\sigma _2=0.27$. The zero-inflation levels for gene $y_1$ and gene $y_2$ are 0.45 and 0.3, respectively. Cell pseudotime *z* is simulated from a uniform distribution (0,27). The number of observations is set to 500 and 1000. The number of iterations is 1000. The results are shown in Fig. 2. The coefficient estimations are more accurate with a larger sample size. Overall, the proposed framework performs well in a linear setting.

7 $$\begin{aligned} \mu _1= & e^{\eta _1}=e^{0.02z-0.4}, \nonumber \\ \mu _2= & e^{\eta _2}=e^{-0.03z+0.3},\nonumber \\ \rho= & \text {tanh}(\eta _3) = \text {tanh}(-0.02z). \end{aligned}$$

#### Scenario II

We conducted the simulation studies in two non-linear scenarios. In the first non-linear scenario, zero-inflated data is generated to mimic the real mouse pituitary gland embryological development data. The responses $y_1$ and $y_2$ are the two genes’ expression levels, and they are simulated from Gamma distributions with zero inflation rates $p_1=0.4$ and $p_2=0.35$, respectively. We sampled cell pseudotime *z* from the uniform distribution with interval (0, 27).

We used the Gaussian copula distribution to model the correlation $\rho$ of two genes’ expression levels $y_1$ and $y_2$; the marginal distributions of $y_1$ and $y_2$ are Gamma distributions, assuming $\sigma _1=0.2$ and $\sigma _2=0.27$. Assume the $\mu _1$, $\mu _2$ and $\rho$ have non-linear relationships with cell pseudotime *z*. The link functions for $\eta _1$ and $\eta _2$ are log functions. We used the inverse hyperbolic tangent function as the link function for the dependence parameter $\eta _3$. The parameter models are given by,

8 $$\begin{aligned} \mu _1 & =e^{\eta _1}=e^{0.02z-0.3}, \nonumber \\ \mu _2 & =e^{\eta _2}=0.5\text {cos}(0.2z-0.1)+1,\nonumber \\ \rho & =\text {tanh}(\eta _3) = \text {tanh}(0.5-e^{-0.2z}). \end{aligned}$$

**Fig. 3:**
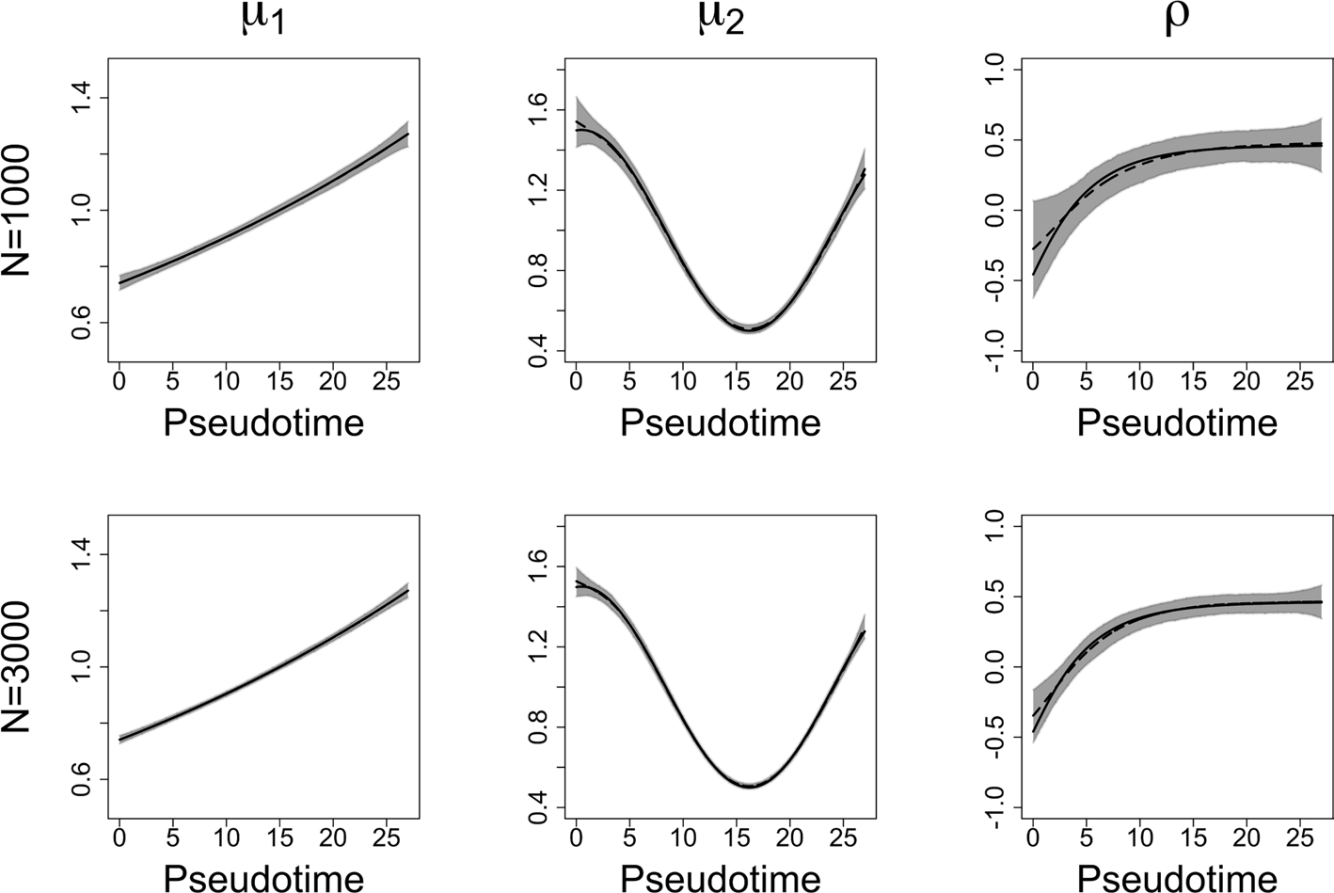
Scenario II simulation: The black solid lines are the true smoothing functions, and the black dashed lines are the mean estimates for 1000 iterations. The shaded areas are point-wise ranges from 5 to 95% quantile. The number of observations is 1000 and 3000 for each row

The hyperbolic tangent function for dependence parameter $\rho$ can rescale the additive predictor to $(-1,1)$. The exponential function is used for marginal distribution parameters to make sure the means are positive values. The number of iterations is set to 1000, and the number of observations is set to 1000 and 3000. In each iteration, a new zero-inflated data set is generated and fitted using the proposed model. The fitted curves are shown in Fig. 3.

In Fig. 3, the black solid lines are the true smoothing functions, and the black dashed lines are the mean estimates for 1000 iterations. The shaded areas are point-wise ranges from 5 to 95% quantile and they cover the true smoothing functions. The figure on the left is for $\mu _1$, the figure in the middle is for $\mu _2$, and the one on the right is for correlation $\rho$. The plots of $\sigma _1$, $\sigma _2$, $p_1$, and $p_2$ are shown in Supplementary Data-Section B.1.

In simulation scenario II, the mean expression level of the first gene increases smoothly as cell pseudotime increases, while the mean expression level of the second gene follows a U-shaped pattern: it decreases initially, reaches its minimum around pseudotime 16, and then increases. The correlation between the two genes starts at around $-$0.5 at pseudotime 0, increases steadily, crosses 0 around pseudotime 5, and becomes positive thereafter. It reaches a peak of about 0.4 around pseudotime 15 and remains stable and positive beyond that point.

For each simulated dataset, we obtained parameter estimates using the fitted model with known cell-level pseudotime $z_i$. Specifically, for each cell $i=1,\dots,N$, the fitted model outputs the estimated parameters: ${\hat{\mu }}_{i1}$, ${\hat{\mu }}_{i2}$, ${\hat{\sigma }}_{i1}$, ${\hat{\sigma }}_{i2}$, $\hat{p}_{i1}$, $\hat{p}_{i2}$, ${\hat{\rho }}_{i}$. Across $I=1000$ simulated datasets, we construct an $N \times I$ matrix $\hat{\varvec{\theta }}_m$ to store the parameter estimates for each parameter, where each row contains the estimates for a specific cell across all 1000 simulations. To evaluate model performance, we compute each parameter’s bias and Root Mean Squared Error (RMSE). The bias is defined as $\frac{1}{N}\sum ^{i=1}_{N}|\frac{1}{I}\sum ^{I}_{l=1}{\hat{\theta }}_{il}-\theta _i|$, and the RMSE is defined as $\frac{1}{N}\sum _{i=1}^{N}\sqrt{\frac{1}{I}\sum ^{I}_{l=1}({\hat{\theta }}_{il}-\theta _i)^2}$, where $\theta _i$ is the true parameter value for cell *i*, and ${\hat{\theta }}_{il}$ is the estimated value for cell *i* in the *l*-th simulated dataset. The bias and RMSE for each parameter are shown in Table 1. The results suggest that the TIME-CoExpress achieves low bias and RMSE across all parameters.

**Table 1:** Bias $\frac{1}{N}\sum ^{i=1}_{N}|\frac{1}{I}\sum ^{I}_{l=1}{\hat{\theta }}_{il}-\theta _i|$ and RMSE $\frac{1}{N}\sum _{i=1}^{N}\sqrt{\frac{1}{I}\sum ^{I}_{l=1}({\hat{\theta }}_{il}-\theta _i)^2}$ for each parameter under Scenario II, calculated across *N* cells and $I=1000$ simulation replicates

	Parameter	Bias	RMSE
$N=1000$	$\mu _{1}$	0.001	0.013
	$\mu _{2}$	0.006	0.026
	$\sigma _{1}$	0.001	0.006
	$\sigma _{2}$	0.002	0.008
	$\rho$	0.023	0.093
	$p_1$	0.001	0.015
	$p_2$	0.000	0.015
	$\mu _{1}$	0.000	0.008
	$\mu _{2}$	0.004	0.016
	$\sigma _{1}$	0.000	0.003
$N=3000$	$\sigma _{2}$	0.001	0.004
	$\rho$	0.013	0.055
	$p_1$	0.000	0.009
	$p_2$	0.000	0.009

The average time to run one iteration is 0.111 min (6.66 s) for 1000 observations and 0.176 min (10.56 s) for 3000 observations. This simulation is conducted on a high-performance computing cluster, with each node configured with 24 CPUs.

#### Scenario III

In this simulation scenario, we simulated zero-inflated data to mimic the experimental dataset where we have the wild-type group data and the mutant group data. For each cell *i*, we defined cell pseudotime as $z_{i}$, and we introduced an additional factor *g* to identify the two groups of data. The cell is from the wild-type group if $g_{i}=1$ and it’s from the mutant group if $g_{i}=2$.

For each cell *i*, we simulated the non-zero values of the two gene expression levels $w_{i1}$ and $w_{i2}$ from a Gaussian copula model mentioned in Section Zero-inflated copula model with gene pair correlation $\rho _i$. Both marginal distributions are set to Gamma distributions with distributional parameters $(\mu _{i1},\sigma _{i1})$ and $(\mu _{i2},\sigma _{i2})$, respectively. The zero-inflation rates of two genes are $p_{i1}$ and $p_{i2}$; they both have a non-linear relationship with cell pseudotime $z_{i}$. To incorporate the zero-inflation characteristic in our simulation, we simulate $D_{i1} \sim \text {Bern}(p_{i1})$ and $D_{i2} \sim \text {Bern}(p_{i2})$. The gene expressions of the gene pair will be $y_{i1}=(1-D_{i1})w_{i1}$ and $y_{i2}=(1-D_{i2})w_{i2}$.

We assumed the mean of gene expression level($\mu$), the correlation of two genes($\rho$), and the gene zero-inflation rates(*p*) change in a non-linear fashion along the cell pseudotime *z*. The parameter simulations are given by,

9 $$\begin{aligned} \mu _1= & (2-g)(0.008z^2-0.2z+2.5)+g(e^{0.08\text {exp}(0.1z)+0.2}),\nonumber \\ \mu _2= & (2-g)(e^{\text {sin}(0.1(z-16))})+g(e^{-0.02z+0.6}),\nonumber \\ \sigma _1= & 0.1g+0.1,\nonumber \\ \sigma _2= & 0.1g+0.2,\nonumber \\ \rho= & (1-g)(\text {tanh}(0.4(0.2z-1)))+g(\text {tanh}(-0.01z+0.2)),\nonumber \\ p_1= & (2-g)(\text {sigmoid}(-0.004z^2+0.037z-0.2))+g(\text {sigmoid}(0.01z-0.9)),\nonumber \\ p_2= & (2-g)(\text {sigmoid}(-0.05z+0.3))+g(\text {sigmoid}(0.01z^2-0.3z-0.1)). \end{aligned}$$

**Fig. 4:**
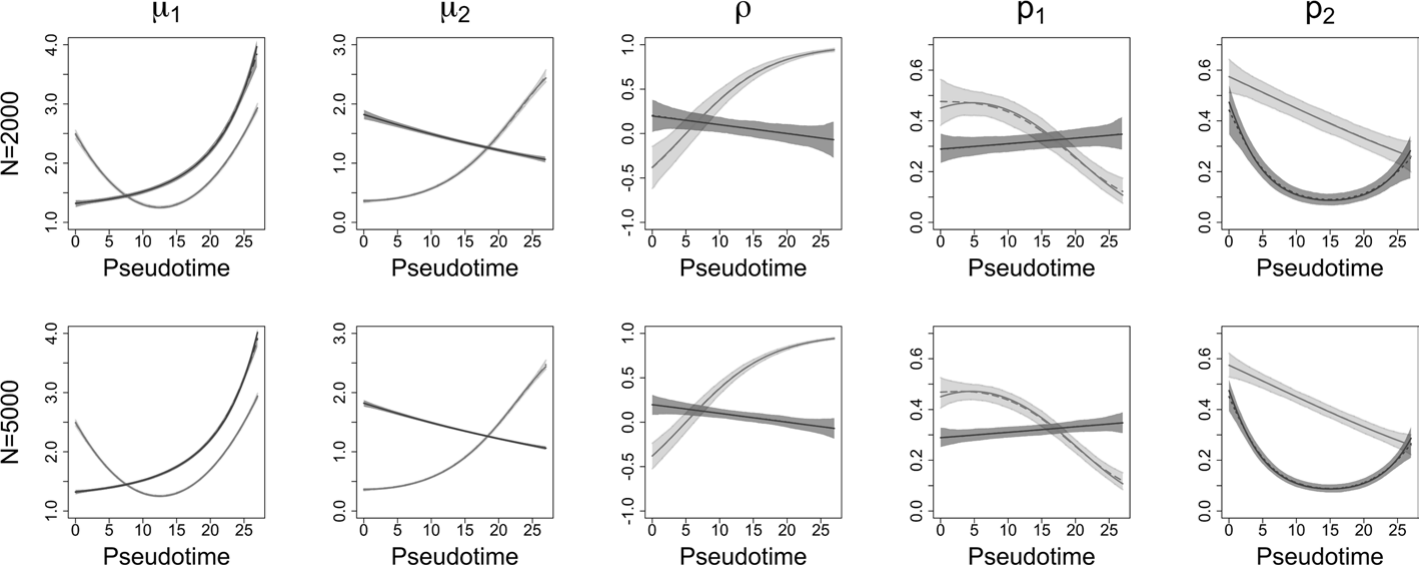
Scenario III simulation: The light gray color is for the wild-type group, and the dark gray color is for the mutant group. The solid lines are the true smoothing functions, and the dashed lines are the mean estimates for 1000 iterations. The shaded areas are point-wise ranges from 5 to 95% quantile. The number of observations is 2000 and 5000 for each row

We sampled cell pseudotime *z* from a uniform distribution with interval (0, 27) based on the experimental data analysis described in Section Experimental data analysis. The simulated functions in Equation (9) and the parameters are also based on the mouse pituitary gland scRNAseq data considered in Section Experimental data analysis. The number of iterations is set to 1000. The sample size is set to 2000 or 5000. The proportions of wild-type group and mutant group data are set to 55% and 45%. The results are shown in Fig. 4. The CV plots will be shown in Supplementary Data–Section B.1.

In Fig. 4, the light gray color is for the wild-type group, and the dark gray color is for the mutant group. The solid lines are the true smoothing functions, and the dashed lines are the mean estimates for 1000 iterations. The shaded areas are point-wise ranges from 5 to 95% quantile. For $\mu _1$, the wild-type group exhibits a U-shaped expression trajectory, with expression levels decreasing until pseudotime 10 and then rising sharply. In contrast, the mutant group displays a steadily increasing trend. For $\mu _2$, the wild-type group’s expression increases over pseudotime, while the mutant group’s expression decreases. The wild-type group shows a smooth increase in co-expression, starting from approximately $-$0.5 at pseudotime 0, crossing 0 around pseudotime 6, and reaching nearly 1 by the end. In comparison, the mutant group shows an overall weaker correlation, with $\rho$ slightly decreasing from 0.2 to $-$0.1 over pseudotime. The distinct non-linear patterns in the zero-inflation rates $p_1$ and $p_2$ suggest dynamic gene on-off regulation during cell differentiation. These simulations demonstrate the capability of TIME-CoExpress to simultaneously model multiple groups of data. The bias and RMSE of each parameter for both the wild-type group and the mutant group are shown in Table 2. The average time to run one iteration is 0.773 min (46.380 s) for 2000 observations and 0.790 min (47.400 s) for 5000 observations.

The results from both scenarios II and III suggest that the 5% to 95% quantile bounds cover the true smoothing functions, and the mean estimates align with the true smoothing functions. Both the bias and the RMSE are small, indicating that the estimations are accurate. Furthermore, bias and the RMSE are smaller with a larger number of observations. These simulation studies show that the TIME-CoExpress can capture the non-linear patterns of response correlations, means and zero-inflation rates.

**Table 2:** Bias $\frac{1}{N}\sum ^{i=1}_{N}|\frac{1}{I}\sum ^{I}_{l=1}{\hat{\theta }}_{il}-\theta _i|$ and RMSE $\frac{1}{N}\sum _{i=1}^{N}\sqrt{\frac{1}{I}\sum ^{I}_{l=1}({\hat{\theta }}_{il}-\theta _i)^2}$ for each parameter under Scenario III, calculated across *N* cells and $I=1000$ simulation replicates

	Parameter	Wild-type		Mutant	
		Bias	RMSE	Bias	RMSE
$N=2000$	$\mu _{1}$	0.005	0.017	0.008	0.034
	$\mu _{2}$	0.005	0.019	0.000	0.024
	$\sigma _{1}$	0.001	0.003	0.001	0.005
	$\sigma _{2}$	0.002	0.006	0.001	0.007
	$\rho$	0.003	0.062	0.001	0.071
	$p_1$	0.066	0.074	0.000	0.024
	$p_2$	0.001	0.028	0.006	0.023
	$\mu _{1}$	0.003	0.011	0.006	0.024
	$\mu _{2}$	0.004	0.013	0.000	0.016
	$\sigma _{1}$	0.000	0.002	0.000	0.003
$N=5000$	$\sigma _{2}$	0.001	0.004	0.000	0.004
	$\rho$	0.001	0.037	0.001	0.044
	$p_1$	0.066	0.070	0.000	0.015
	$p_2$	0.000	0.017	0.004	0.015

#### Scenario IV

In this Scenario, we evaluated the statistical power of the proposed framework by comparing it with scHOT [1], CNM [18], and liquid association (LA) proposed by Li [17]. We designed a simulation with a correlation function shown in Equation (10).

10 $$\begin{aligned} \rho =\text {tanh}(\eta ) = \text {tanh}(C(0.5\text {sin}(2\pi z)-0.8 + 1.6z)). \end{aligned}$$

This function contains a constant scaling factor *C*. When *C* is small, the correlation function is close to zero; as *C* increases, the correlation function becomes more and more different from $\rho =0$. We set the means of two genes’ expressions as $\mu _1=5$, $\mu _2=3$ and CV $\sigma _1=\frac{1}{3}$ and $\sigma _2=\frac{1}{2}$. The zero-inflation rates for these two genes are $p_1=0.3$ and $p_2=0.4$.

**Fig. 5:**
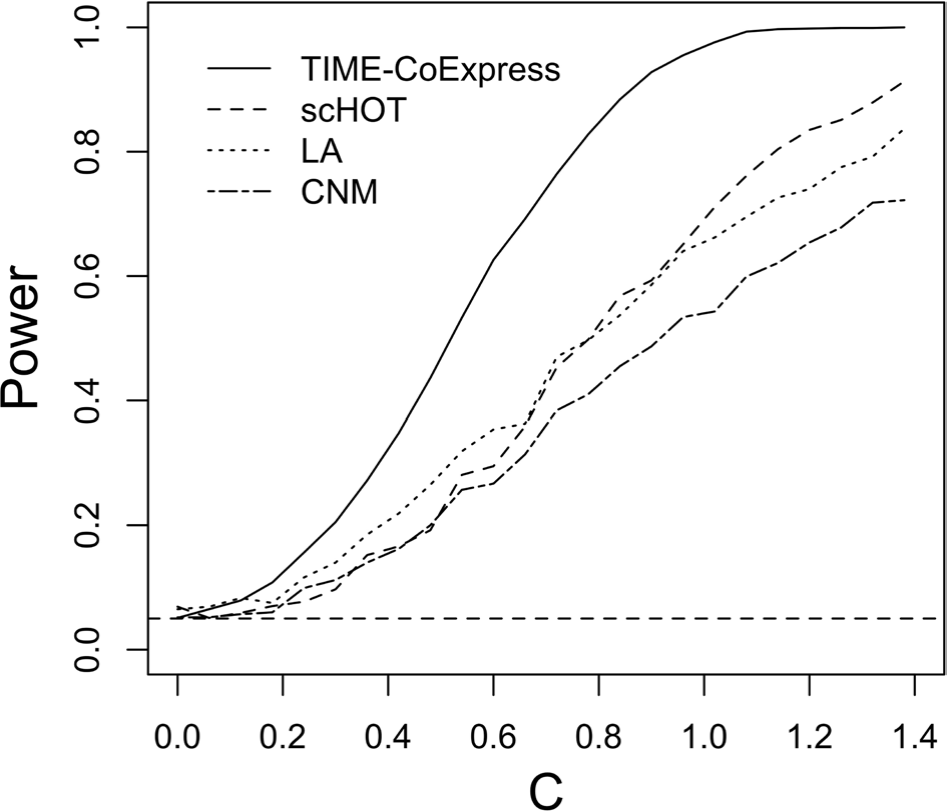
Scenario IV simulation: The power comparison of TIME-CoExpress, scHOT, CNM, and LA (with permutations). The number of observations is 1000, and the number of iterations is 1000. The power is defined as the rate of rejecting the null hypothesis when testing $S(z)=0$

The null hypothesis is defined as,

11 $$\begin{aligned} H_0: S(z)=0, \end{aligned}$$

where function *S*() is the correlation function in Equation (10). We simulated 1000 observations, and fit these four methods 1000 times for each value of *C*. We calculated the model power, defined as the proportion of simulations rejecting the null hypothesis, for different values of the constant *C*. The resulting power plot is shown in Fig. 5. The results presented in Fig. 5 show that when $C=0$, all methods maintain proper type I error at the nominal level (0.05). In addition, this power analysis also indicates that our proposed method outperforms the other three methods. The power of our method reaches 1 faster as *C* increases compared to other methods.

## Experimental data analysis

In this section, we apply the TIME-CoExpress model to embryological scRNAseq data described in Section Experimental dataset. Our analysis aims to investigate the impact of *Nxn* mutation on mouse pituitary gland development by identifying differences in gene co-expression along cellular temporal trajectories between wild-type and mutant mice, incorporating changes in zero inflation rate along the cell temporal trajectory.

We considered two gene lists in our analysis. The first list of genes is derived from the WNT signaling pathway (mmu04310) in the KEGG database [39–41]. *Nxn* is known to regulate Disheveled, a key intracellular mediator of the WNT signaling pathway [25]. *Wnt5a* is expressed in the embryonic pituitary gland and ${Wnt5a}^{-/-}$ embryos have malformed pituitary glands [42]. Therefore, we selected genes from the *Wnt5a* gene pathway for analysis using TIME-CoExpress. We removed genes from the gene list with more than 80% zeros since genes with zero expression in most cells do not provide any information. A total of 50 genes (1225 gene pairs) are selected. We studied all possible gene pair combinations by fitting our model to each pair and identified gene pairs with different co-expression patterns between wild-type and mutant groups along the cellular pseudotime. The second gene list is from pituitary TFs, and we mainly focused on evaluating zero-inflation rates for each gene along pseudotime. A total of 55 pituitary gland TFs (1485 combinations) are used. The lists of the 50 genes related to the WNT signaling pathway and the 55 TF can be found in Supplementary Data–Section B.5.

We applied the Slingshot algorithm to estimate the temporal trajectory for all cells. This method fits a single principal curve for all cells based on gene expression levels and produces a pseudotime ordering of cells, which is determined by their positions when projected onto the curve. The cell cluster information is not provided to Slingshot since it is an unsupervised method. Other pseudotime inference methods like TSCAN [7] and Monocle [6] can also be used.

For each gene pair with expression levels $y_1$ and $y_2$, we fit the proposed framework with cell pseudotime (*z*) and group (*g*). The model provided correlation estimates for each gene pair along pseudotime for both wild-type and mutant groups. This multi-group analysis enables a direct comparison between different groups. To evaluate the difference between the two correlation curves, a *p*-value is calculated to test the null hypothesis that the correlation trajectories between the wild-type and mutant groups are identical. Besides the gene pair co-expression, our model also evaluates how zero-inflation rates change through cell pseudotime for individual genes. For each gene, our model provides *p*-values to assess whether the zero-inflation rate differs significantly between the wild-type and mutant groups. To control the false discovery rate (FDR), we use the Benjamini-Hochberg (BH) procedure to adjust the *p*-values across all gene pairs. We consider the gene pairs with adjusted *p*-values less than 0.05 for significance. A total of 42 gene pairs were declared to have significant temporal co-expression changes between wild-type and mutant among all 1,225 gene pairs from the WNT pathway.

We also considered a second metric to evaluate the difference between the estimated correlation curves ($\Delta|{\hat{\rho }}|$):

12 $$\begin{aligned} \Delta|{\hat{\rho }}| = \frac{\sum _{i=1}^{n_{\text {time}}}|{\hat{\rho }} _{\text {wild-type},i}-{\hat{\rho }} _{\text {mutant},i}|}{n_{\text {time}}}, \end{aligned}$$

where $n_{\text {time}}$ is the number of pseudotime intervals we considered, ${\hat{\rho }} _{\text {wild-type}}$ and ${\hat{\rho }} _{\text {mutant}}$ are the gene pair correlation predictions for the wild-type group and the mutant group respectively. Due to inherent differences between cells in the wild-type and mutant groups, their respective cell pseudotimes varied. To facilitate a meaningful comparison of the dynamic correlation between these two groups, we generate evenly spaced pseudotime intervals to predict the dynamic correlation using the fitted model to calculate $\Delta|{\hat{\rho }}|$.

Table 3 shows the 42 significant gene pairs co-expression with the smallest adjusted *p*-value between the wild-type group and the mutant group. $|\Delta {\hat{\rho }} _{\text {diff,max}}|$ is defined as the maximum predicted correlation difference between these two groups. The range of correlation is between -1 and 1, and the maximum number $|\Delta {\hat{\rho }} _{\text {diff,max}}|$ can be no larger than 2. AUD is the difference in the area under the predicted correlation curves between the mutant versus wild-type. The larger the AUD, the greater the difference between the two predicted correlation curves.

**Table 3:** Top 42 significant gene pairs with adjusted *p*-value less than 0.05

	Gene 1	Gene 2	*p*-value	Adjusted *p*-value	${\Delta|{\hat{\rho }}|}$	${|\Delta {\hat{\rho }} _{{diff,max}}|}$	AUD
1	*Apc*	*Fbxw11*	< 0.001	< 0.001	0.27	0.58	8.10
2	*Apc*	*Senp2*	< 0.001	< 0.001	0.37	0.81	10.95
3	*Btrc*	*Ctbp1*	< 0.001	< 0.001	0.30	0.62	9.10
4	*Btrc*	*Fzd3*	< 0.001	< 0.001	0.43	0.90	12.73
5	*Btrc*	*Lef1*	< 0.001	< 0.001	0.49	0.99	14.69
6	*Btrc*	*Senp2*	< 0.001	< 0.001	0.75	1.45	22.48
7	*Camk2d*	*Tbl1x*	< 0.001	< 0.001	0.37	0.64	11.18
8	*Ccnd2*	*Lrp6*	< 0.001	< 0.001	0.24	0.63	7.25
9	*Ccnd3*	*Siah1a*	< 0.001	< 0.001	0.11	0.25	3.16
10	*Crebbp*	*Fbxw11*	< 0.001	< 0.001	0.52	0.89	15.67
11	*Csnk1e*	*Lgr4*	< 0.001	< 0.001	0.17	0.34	4.94
12	*Csnk1e*	*Siah1a*	< 0.001	< 0.001	0.13	0.29	3.86
13	*Csnk2a1*	*Lrp6*	< 0.001	< 0.001	0.22	0.61	6.66
14	*Csnk2a2*	*Rock2*	< 0.001	< 0.001	0.43	0.95	12.79
15	*Csnk2b*	*Siah1a*	< 0.001	< 0.001	0.15	0.41	4.63
16	*Ctbp1*	*Lrp6*	< 0.001	< 0.001	0.13	0.26	3.94
17	*Ctbp1*	*Ppp3cb*	< 0.001	< 0.001	0.28	0.65	8.51
18	*Ctnnb1*	*Jun*	< 0.001	< 0.001	0.30	0.67	9.01
19	*Ctnnbip1*	*Siah1a*	< 0.001	< 0.001	0.11	0.34	3.25
20	*Ctnnd2*	*Siah1a*	< 0.001	< 0.001	0.46	0.83	13.76
21	*Ep300*	*Lgr4*	< 0.001	< 0.001	0.25	0.56	7.42

	Gene 1	Gene 2	*p*-**value**	Adjusted *p*-**value**	$\varvec{\Delta|{\hat{\rho }}|}$	${|\Delta {\hat{\rho }} _{{{diff,max}}}|}$	AUD
22	*Fzd3*	*Ryk*	< 0.001	< 0.001	0.17	0.39	5.06
23	*Fzd3*	*Tle1*	< 0.001	<0.001	0.23	0.45	6.78
24	*Gsk3b*	*Lgr4*	< 0.001	< 0.001	0.18	0.37	5.23
25	*Gsk3b*	*Prickle1*	< 0.001	< 0.001	0.26	0.75	7.90
26	*Gsk3b*	*Senp2*	< 0.001	< 0.001	0.13	0.29	3.93
27	*Lrp6*	*Prkacb*	< 0.001	< 0.001	0.47	1.06	14.15
28	*Plcb4*	*Prkaca*	< 0.001	< 0.001	0.27	0.62	8.00
29	*Ppp3ca*	*Ryk*	< 0.001	< 0.001	0.12	0.24	3.47
30	*Ruvbl1*	*Senp2*	< 0.001	<0.001	0.30	0.58	8.86
31	*Ccnd3*	*Fbxw11*	< 0.001	< 0.001	0.14	0.30	4.29
32	*Gsk3b*	*Tbl1x*	< 0.001	< 0.001	0.30	0.56	8.90
33	*Ctbp1*	*Fzd3*	< 0.001	< 0.001	0.15	0.30	4.39
34	*Rock2*	*Ryk*	< 0.001	< 0.001	0.64	1.17	19.10
35	*Ppp3ca*	*Senp2*	< 0.001	< 0.001	0.17	0.46	4.98
36	*Camk2d*	*Fbxw11*	< 0.001	< 0.001	0.93	1.60	27.87
37	*Csnk2a2*	*Lef1*	< 0.001	< 0.001	0.17	0.37	5.16
38	*Ctnnd2*	*Tcf7l2*	< 0.001	0.003	0.52	0.97	15.57
39	*Rac1*	*Senp2*	< 0.001	0.004	0.32	0.54	9.52
40	*Lgr4*	*Tcf7l2*	< 0.001	0.023	0.21	0.54	6.34
41	*Prkaca*	*Tcf7l2*	< 0.001	0.030	0.64	1.22	19.28
42	*Cby1*	*Siah1a*	<0.001	0.035	0.60	1.28	18.10

$\Delta|{\hat{\rho }}|$ is the overall correlation difference between the wild-type group and the mutant group; $|\Delta {\hat{\rho }} _{\text {diff,max}}|$ is the max predicted correlation difference between these two groups; AUD is the area under the correlation difference curve

**Fig. 6:**
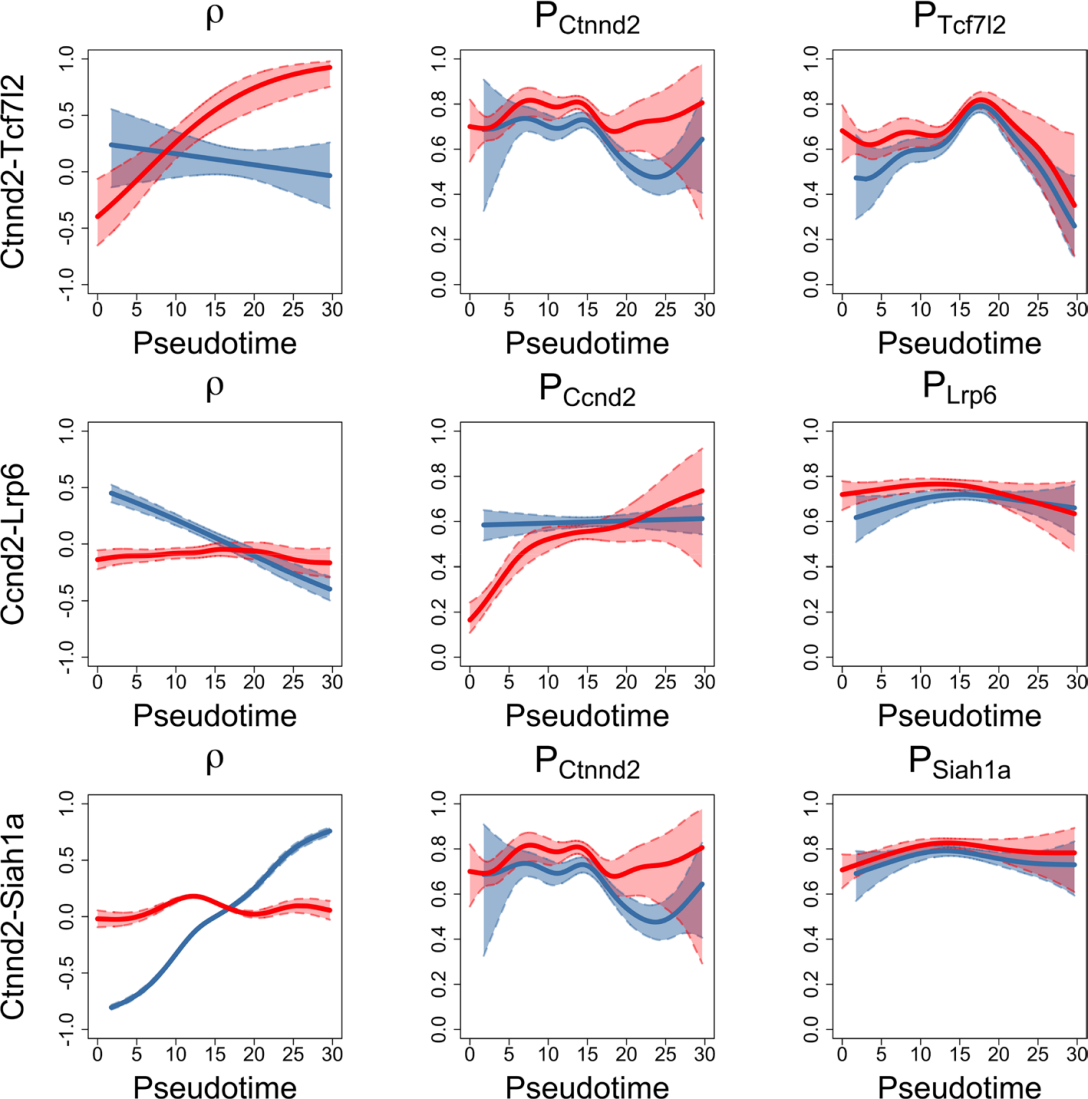
Significant gene pairs examples. The figures in the first column show how gene pair correlations ($\rho$) change along the cellular pseudotime. The figures in the second and third columns show how gene zero inflation rates ($P_{(\cdot )}$) change along the cellular pseudotime. The blue solid curve is the fitted line for the wild-type group, and the blue dashed line is the 95% CI of the fit. The red solid curve is the fitted line for the mutant group, and the red dashed line is the 95% CI of the fit

We show three gene pairs as examples from our analysis (*Ctnnd2* and *Tcf712*; *Ccnd2* and *Lrp6*; *Ctnnd2* and *Siah1a*) in Fig. 6. These selected gene pairs demonstrate significant correlation differences between wild-type (blue) and mutant (red) groups. The solid curves represent how gene pair correlations ($\rho$) or gene zero inflation rates ($P_{(\cdot )}$) change along the cellular pseudotime in both the wild-type group and mutant group, where the x-axis values are the pseudotime generated from Slingshot. The shaded areas represent the 95% CI of the fit. More gene pair correlation plots are available in Supplementary Data–Section B.2.

We use quantile–quantile (Q–Q) plots to evaluate the model’s goodness-of-fit. It provides a visual comparison between the observed and predicted correlation distributions for each gene pair. The Q–Q plots for the gene pairs fitting are presented in Supplementary Data–Section B.3. The data points align around the $y=x$ line, indicating the model accurately captured the distributional characteristics of the data across pseudotime.

Based on Table 3 and Fig. 6, the TIME-CoExpress analysis identifies gene pairs that are likely involved in the differentiation process. For instance, *Ctnnd2*, or Delta-catenin, and the ubiquitin ligase *Siah1a* are negatively correlated in *Sox2*-expressing stem cells but have an increasing correlation as pituitary stem cells differentiate towards the *Pou1f1* expressing progenitor stage. In ${Nxn}^{-/-}$ embryos, these two genes’ expression patterns are not correlated. The expression correlation for *Ctnnd2* and the TF *Tcf7l2* decreases in wild-type embryos during differentiation, while it is dramatically increased in ${Nxn}^{-/-}$ embryos. These results suggest that *Ctnnd2* expression in combination with *Siah1a* promotes differentiation, while *Ctnnd2* expression with *Tcf7l2* maintains the stem cell state, which are experimentally testable hypotheses. The expressions of *Ccnd2* and *Lrp6* are correlated in stem cells in the control group, but this correlation weakens as differentiation progresses. In the mutant group, *Ccnd2* and *Lrp6* show no significant correlation. The distinct correlation patterns in the control and mutant groups emphasize the impact of the mutation on gene pair co-expression during differentiation.

**Fig. 7:**
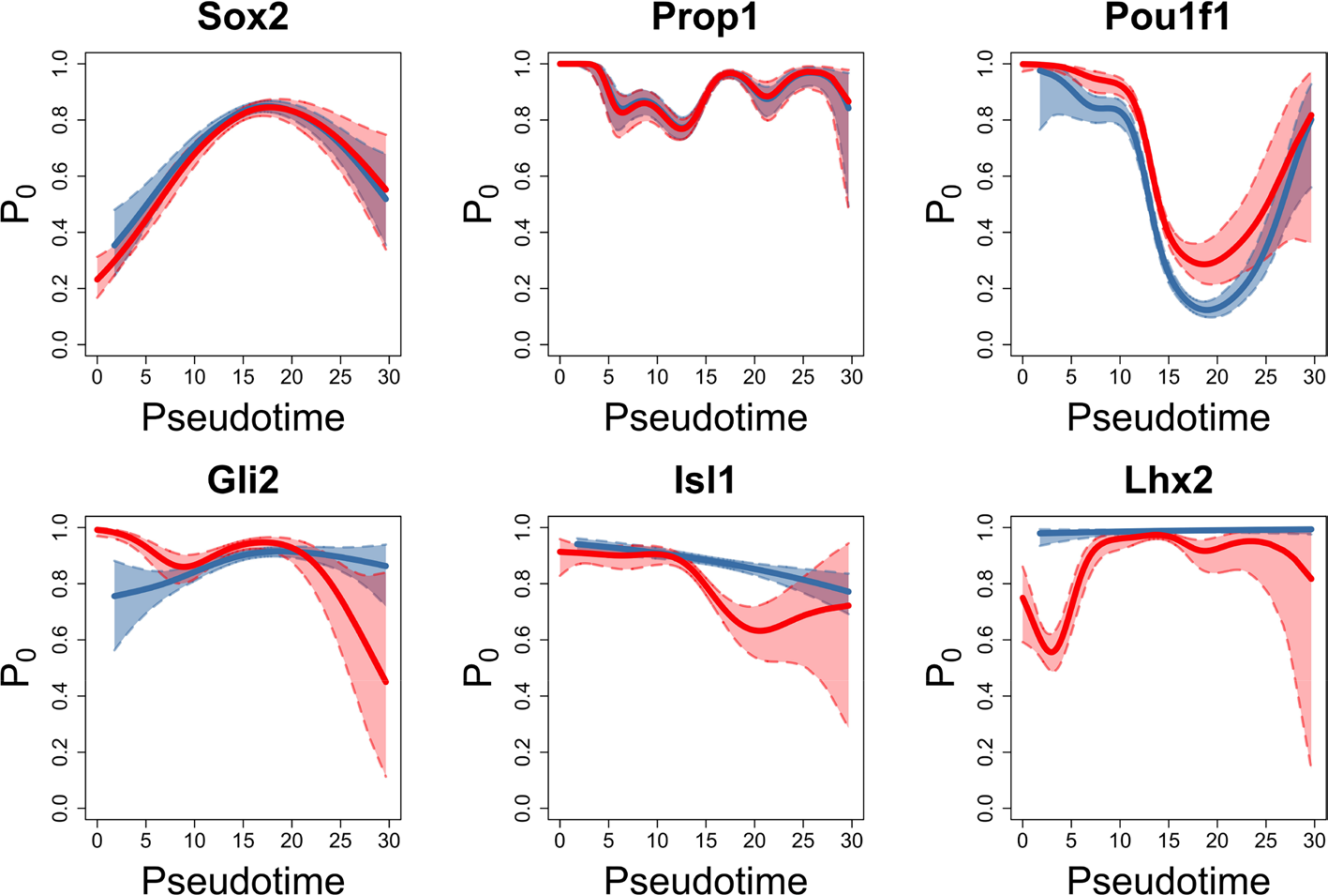
Examples of zero-inflation rate ($P_0$) changes from important TFs. The blue solid curve is the fitted line for the wild-type group, and the blue dashed line is 95% CI of the fit. The red solid curve is the fitted line for the mutant group, and the red dashed line is 95% CI of the fit

In the gene zero-inflation analysis, we utilized TFs from the dataset to reveal how the zero-inflation rate ($P_0$) changes for each gene along pseudotime. The figures in the first row in Fig. 7 present how the zero-inflation rates of *Sox2*, *Prop1*, and *Pou1f1* progenitors change through cell pseudotime. The figures in the second row in Fig. 7 present three TFs: *Gli2*, *Isl1*, *Lhx2*, with a small *p*-value of zero inflation rates between mutant versus wild-type. More genes’ zero-inflation rate plots can be found in Supplementary Data–Section B.4.

All analyses were performed using high-performance computing resources, with each gene pair computation averaging approximately 4.463 min (267.780 s). The computational setup consisted of 200 nodes running simultaneously, each node utilizing 24 CPUs to parallelize the workload. To test TIME-CoExpress, we limited the analysis by providing a relatively short gene list associated with WNT.

## Discussion

Understanding how gene pair co-expression changes along the cellular temporal trajectories is crucial for gaining deeper insights into the dynamic interactions within biological systems. In this paper, we move beyond the traditional one-gene-at-a-time analysis and introduce TIME-CoExpress, a flexible, zero-inflated, copula-based, analytical framework designed to capture dynamic shifts in gene co-expression across cellular pseudotime.

Unlike traditional methods, which often assume gene co-expression to be static rather than time-dependent, TIME-CoExpress accounts for the non-linear characteristic of gene-to-gene interactions through a Gaussian copula-based model. This capability is important in biological analysis, as genes exhibit complex temporal changes in gene interactions within a living cell. Moreover, the proposed framework accommodates covariate-dependent zero-inflation, allowing the zero-inflation rate to vary across cell pseudotime. Tracking these changes provides insights into gene activation and deactivation throughout cell differentiation. By incorporating the non-linear changes of zero-inflation, TIME-CoExpress provides a more accurate representation of gene pair co-expression dynamics along the temporal trajectory.

The popularity of pseudotime inference enables researchers to investigate gene expression changes along a continuous cellular trajectory. In this paper, we employ the Slingshot method [8] to infer cell pseudotime from scRNAseq data. Slingshot is compatible with handling bifurcations or multiple branching in cellular trajectories, and it is flexible to use because it does not require predefined clusters or starting points. Additionally, it is not sensitive to outliers and noise due to its use of principal curves. Other pseudotime inference methods, such as TSCAN [7], and Monocle [6], may also be applied depending on the user’s needs. Our proposed framework is also applicable to real cell time if such data is available.

TIME-CoExpress is designed to analyze scRNAseq data from multiple groups simultaneously in a unified analytical framework. In contrast, existing methods such as scHOT are limited to analyzing only one group at a time, making comparisons between different treatment conditions challenging. TIME-CoExpress offers more comprehensive insights by including direct comparisons across different experimental conditions in the same model framework.

We conducted a series of simulation analyses to demonstrate the effectiveness of our copula-based framework. The results show that TIME-CoExpress is capable of capturing dynamic correlation changes between two covariates over time. Additionally, the simulation studies illustrate that the framework effectively models dynamic zero-inflation patterns of genes as they change across cell pseudotime.

In the experimental data analysis, we applied the proposed model to experimental data generated from mouse pituitary gland embryogenesis. We compared wild-type mouse embryos with *Nxn* mutant embryos in terms of gene co-expression and zero-inflation rates along the cellular temporal trajectory. In this analysis, cell pseudotime was used as the covariate. Other covariates, such as cell status, can also be incorporated into the model.

Our model identified differentially co-expressed gene pair combinations along the cell temporal trajectory between ${Nxn}^{-/-}$ and wild-type mice and uncovered several gene pair interactions that may help explain the observed phenotypic differences. TIME-CoExpress provides a more detailed view of how genes interact over time during the mouse embryological development. Using this innovative framework, we also uncovered gene zero-inflation patterns that underlie developmental differences and demonstrated the dynamic transcriptional regulation that occurs during organogenesis of the mouse pituitary gland. The altered developmental trajectory for pituitary stem cells is consistent with and supported by experimental data [26]. Our TIME-CoExpress further identifies gene pairs whose transcriptional changes may underlie the altered developmental trajectory.

The goodness of fit for our model is assessed through residual analysis and Q-Q plots, allowing us to visually compare the distribution of model-predicted gene co-expression values with observed data across cell pseudotime. The Q-Q plot of the residuals follows a nearly straight line, suggesting a good fit to the data and validating the correctness of our model in capturing the gene co-expression changes across cellular temporal trajectories.

Additionally, Q-Q plots serve as a useful tool for parameter tuning. There are several parameter selections we need to consider in the zero-inflated copula model established in Section Methods. The inflation factor of splines controls the smoothness of the fitted curves. A larger inflation factor gives smoother estimates. We tune these parameters iteratively through visual inspection, adjusting them until the Q–Q plot indicates an optimal fit. This process helps ensure that the model accurately captures the dynamic, non-linear patterns of gene co-expression and zero-inflation in the data. In this model, we use thin plate splines, other splines are also available to use. The smoothing parameter $\lambda$ controls the trade-off between fit and smoothness. A large $\lambda$ gives high smoothness results but may underfit the data, while a small $\lambda$ gives a better fit but increases the risk of overfitting. Another parameter is the basis dimension, which affects whether the model can capture the complexity of the underlying data. The number of basis functions also affects the smoothness of fit; a greater number of basis functions can improve the accuracy of the fit, but it may also lead to overfitting. In practice, users can use the Q-Q plot and visual inspection as a guide for tuning the number of basis functions.

The marginal distributions of the copula model are set to Gamma distributions since gene expressions in mouse pituitary gland embryological scRNAseq data are normalized continuous values and skewed. Other marginal distributions can also be applied in the TIME-CoExpress model, depending on the data under study. For example, the negative binomial distribution may be used as the marginal if the data are count-based. The Tweedie distribution is another option and can be easily implemented in our framework.

Our proposed method has higher power in detecting the difference between two correlation curves compared to scHOT [1], LA with permutations [17], and CNM [18]. In addition to improved power, TIME-CoExpress is also more computationally efficient. Under a simulation of 1000 observations and 1000 iterations in Scenario IV, TIME-CoExpress took an average of 11.23 s per iteration. In comparison, scHOT, LA(with permutations), and CNM using a generalized estimation equations (GEE)-based approach [18] required approximately 84.48, 14.58, and 0.082 s per iteration, respectively. While CNM is computationally fast, it has low statistical power. Among the methods evaluated, scHOT is more computationally intensive.

TIME-CoExpress is designed to detect dynamic co-expression changes between gene pairs along a temporal trajectory. It begins by modeling changes in pairwise genetic interactions, which can then be formed into networks. The model captures non-linear changes in mean expression levels, correlations, and gene-specific zero-inflation rates across cellular pseudotime. In addition, it accommodates the zero-inflation and over-dispersion inherent to scRNAseq data. While other methods, such as SCODE and WGCNA, focus on constructing overall gene regulatory networks, they do not capture dynamic, non-linear co-expression changes over cell pseudotime. There are several practical strategies for implementing TIME-CoExpress in typical whole-genome scRNAseq datasets with thousands of genes. (i) Pathway-based search: consider candidate genes in pathways associated with the relevant experimental condition or covariates of interest. For example, our analysis focuses on genes from the WNT signaling pathway and TFs related to *Nxn* mutation. (ii) All possible gene-pair combinations search: To reduce computational cost, pre-screening approaches, such as the Liquid Association Coefficient (LAC) [43], and the fastLA [18] can be used in conjunction with TIME-Coexpress. LAC is calculated as the difference between the Pearson correlation of squared (or absolute) gene expression values and the square (or absolute) of the original correlation. fastLA measures the difference in Pearson correlation of gene expression values between early and late time points.

## Conclusions

In this study, we propose TIME-CoExpress, a flexible, copula-based framework for identifying dynamic gene co-expression patterns along the cellular temporal trajectory in single-cell transcriptomics data. TIME-CoExpress accommodates zero-inflation and over-dispersion characteristics inherent in scRNAseq data, enabling more accurate and biologically meaningful analyses of gene co-expression dynamics, along with other gene expression features such as mean expression levels and dropout rates.

Unlike traditional single-group analyses, our framework supports direct multi-group comparisons, allowing the identification of differential co-expression patterns across various biological conditions or experimental groups. Through simulation studies, we demonstrate that TIME-CoExpress accurately captures non-linear co-expression relationships, correctly models zero-inflation rates and mean expression levels, and outperforms existing methods in statistical power.

In the mouse pituitary gland scRNAseq data analysis, TIME-CoExpress identified gene pairs exhibiting differential co-expression trajectories along cell pseudotime between ${Nxn}^{-/-}$ and wild-type mice. Furthermore, temporal changes in gene-specific zero-inflation rates provided insights into gene activation and silencing during cell development.

Gene regulation is dynamic and complex. TIME-CoExpress offers a valuable and comprehensive tool for understanding how genes interact dynamically over time in transcriptomic data. It can be applied in various biological domains beyond temporal datasets, such as cancer progression and TF regulatory networks.

## Additional file

Supplementary file 1 (pdf 22942 KB)

## Data Availability

Single-cell RNA sequencing data generated in association with this manuscript can be accessed through GEO Database, using series accession number GSE246211 (https://www.ncbi.nlm.nih.gov/geo/query/acc.cgi?acc=GSE246211) [44], and samples GSM7864906 (https://www.ncbi.nlm.nih.gov/geo/query/acc.cgi?acc=GSM7864906) [45], and GSM7864907 (https://www.ncbi.nlm.nih.gov/geo/query/acc.cgi?acc=GSM7864907) [46]. The mutant data can be accessed using accession number GSE281783 and GSM8628052. TIME-CoExpress code can be found at https://github.com/YenYiHo-Lab/TIME-CoExpress.git

## Abbreviations

scRNAseq: Single-cell RNA sequencing
ZENCO: Zero-inflated negative binomial dynamic correlation model
ODEs: Ordinary differential equations
GAMLSS: Additive models for location, scale, and shape
CNM: Conditional normal model
TFs: Transcription factors
DVL: Dishevelled
GA: Gamma
CV: Coefficient of variation
LA: Liquid association
LAC: Liquid association coefficient
FDR: False discovery rate
BH: Benjamini–Hochber
Q–Q: Quantile–quantile
