# Supplementary material for: Time-coexpress: temporal trajectory modeling of dynamic gene co-expression patterns using single-cell transcriptomics data
Source: BMC Bioinformatics. 2025 Jul 29;26:199. doi: 10.1186/s12859-025-06218-w (PMC12308957; doi:10.1186/s12859-025-06218-w)
Supplement: Supplementary file 1 — (pdf 22942 KB) [file 12859_2025_6218_MOESM1_ESM.pdf]

A Supplementary Methods

A.1 Marginal distribution

The marginal distributions can be specified as negative binomial distributions if the data are count-based. The corresponding PDF and CDF are given by Rigby et al. (2019),

$$f_{\mathrm{NB}}(w;\mu,\sigma)=\frac{\Gamma(w+\sigma^{-1})}{\Gamma(\sigma^{-1})\Gamma(w+1)}(\frac{\sigma\mu}{1+\sigma\mu})^{w}(\frac{1}{1+\sigma\mu})^{\sigma^{-1}},$$

$$F_{\mathrm{NB}}(w;\mu,\sigma)=1-\frac{\mathrm{B}(w+1,\sigma^{-1},\mu\sigma(1+\mu\sigma)^{-1})}{\mathrm{B}(w+1,\sigma^{-1})},$$

where B() is the beta function.

A.2 Detailed model

This section provides a detailed description of the proposed framework.

To incorporate gene zero-inflation characteristics into the model, let $D_{ij}\sim\mathrm{Bern}\,(p_{ij})$, $j=1,2$, denote the random variable indicating whether gene j is zeroed out in cell i due to dropout. The gene expression levels can then be modeled as follows:

$$Y_{i1}=(1-D_{i1})W_{i1},$$
$$Y_{i2}=(1-D_{i2})W_{i2}.$$

The joint CDF is given by:

$$\begin{aligned}P\left(Y_{i1}\le y_{i1},Y_{i2}\le y_{i2}\right)=&P\left(Y_{i1}\le y_{i1},Y_{i2}\le y_{i2}\mid D_{i1}=1,D_{i2}=1\right)P(D_{i1}=1)P(D_{i2}=1)\\&+P\left(Y_{i1}\le y_{i1},Y_{i2}\le y_{i2}\mid D_{i1}=1,D_{i2}=0\right)P(D_{i1}=1)P(D_{i2}=0)\\&+P\left(Y_{i1}\le y_{i1},Y_{i2}\le y_{i2}\mid D_{i1}=0,D_{i2}=1\right)P(D_{i1}=0)P(D_{i2}=1)\\&+P\left(Y_{i1}\le y_{i1},Y_{i2}\le y_{i2}\mid D_{i1}=0,D_{i2}=0\right)P(D_{i1}=0)P(D_{i2}=0),\\=&1\cdot p_{i1}p_{i2}\\&+P\left(Y_{i2}\le y_{i2}\,|D_{i2}=0\right)p_{i1}(1-p_{i2})\\&+P\left(Y_{i1}\le y_{i1}\,|D_{i1}=0\right)(1-p_{i1})p_{i2}\\&+P\left(Y_{i1}\le y_{i1},Y_{i2}\le y_{i2}\mid D_{i1}=0,D_{i2}=0\right)(1-p_{i1})(1-p_{i2}),\\=&p_{i1}p_{i2}\\&+P\left(W_{i2}\le y_{i2}\right)p_{i1}(1-p_{i2})\\&+P\left(W_{i1}\le y_{i1}\right)(1-p_{i1})p_{i2}\\&+P\left(W_{i1}\le y_{i1},W_{i2}\le y_{i2}\right)(1-p_{i1})(1-p_{i2}),\end{aligned}$$

which can be simplified as:

$$P\left(Y_{i1}\le y_{i1},Y_{i2}\le y_{i2}\right)=\begin{cases}p_{i1}p_{i2} & \text{if } y_{i1}=0 \text{ and } y_{i2}=0,\\ p_{i1}p_{i2}+F_{\mathrm{GA}}\left(y_{i1};\mu_{i1},\sigma_{i1}\right)(1-p_{i1})p_{i2} & \text{if } y_{i1}>0 \text{ and } y_{i2}=0,\\ p_{i1}p_{i2}+F_{\mathrm{GA}}\left(y_{i2};\mu_{i2},\sigma_{i2}\right)p_{i1}(1-p_{i2}) & \text{if } y_{i1}=0 \text{ and } y_{i2}>0,\\ p_{i1}p_{i2}+F_{W_{i1},W_{i2}}\left(y_{i1},y_{i2};\ \mu_{i1},\mu_{i2},\sigma_{i1},\sigma_{i2},\rho_i\right)(1-p_{i1})(1-p_{i2}) & \text{if } y_{i1}>0 \text{ and } y_{i2}>0.\end{cases}$$

The log-likelihood can be expressed as:

$$\begin{aligned}\ell_i&=\ell\left(\mu_{i1},\mu_{i2},\sigma_{i1},\sigma_{i2},\rho_i,p_{i1},p_{i2};y_{i1},y_{i2}\right)\\&=\begin{cases}\log(p_{i1})+\log(p_{i2}) & \text{if } y_{i1}=0 \text{ and } y_{i2}=0,\\ \log\left(f_{\mathrm{GA}}\left(y_{i1};\mu_{i1},\sigma_{i1}\right)\right)+\log\left(1-p_{i1}\right)+\log\left(p_{i2}\right) & \text{if } y_{i1}>0 \text{ and } y_{i2}=0,\\ \log\left(f_{\mathrm{GA}}\left(y_{i2};\mu_{i2},\sigma_{i2}\right)\right)+\log\left(p_{i1}\right)+\log\left(1-p_{i2}\right) & \text{if } y_{i1}=0 \text{ and } y_{i2}>0,\\ \log\left(f_{W_{i1},W_{i2}}\left(y_{i1},y_{i2};\ \mu_{i1},\mu_{i2},\sigma_{i1},\sigma_{i2},\rho_i\right)\right)+\log\left(1-p_{i1}\right)+\log\left(1-p_{i2}\right) & \text{if } y_{i1}>0 \text{ and } y_{i2}>0.\end{cases}\end{aligned}$$

Since p_{i1},p_{i2} appear solely as additive constants in the log-likelihood function, the estimation of parameters $\mu_{i1},\mu_{i2},\sigma_{i1},\sigma_{i2},\rho_i$ does not depend on their values. Therefore, the log-likelihood can be simplified as follows:

$$\ell_i=c_i+\begin{cases}0 & \text{if } y_{i1}=0 \text{ and } y_{i2}=0,\\ \log\left(f_{\mathrm{GA}}\left(y_{i1};\mu_{i1},\sigma_{i1}\right)\right) & \text{if } y_{i1}>0 \text{ and } y_{i2}=0,\\ \log\left(f_{\mathrm{GA}}\left(y_{i2};\mu_{i2},\sigma_{i2}\right)\right) & \text{if } y_{i1}=0 \text{ and } y_{i2}>0,\\ \log\left(f_{W_{i1},W_{i2}}\left(y_{i1},y_{i2};\mu_{i1},\mu_{i2},\sigma_{i1},\sigma_{i2},\rho_i\right)\right) & \text{if } y_{i1}>0 \text{ and } y_{i2}>0.\end{cases}$$

The estimation of p_{i1} and p_{i2} can then be carried out in a separate step.

Recall from Section 2.2.2 that the parameters of our model are made functions of covariates in the following way:

$$\eta_i^{(\theta_m)} = \beta_1^{(\theta_m)} + \sum_{g=2}^{2} \mathbb{1}(g_i = g)\beta_g^{(\theta_m)} + \sum_{g=1}^{2}\sum_{j_k=1}^{J_k} \mathbb{1}(g_i = g)\beta_{j_k,g}^{(\theta_m)} b_{j_k,g}^{(\theta_m)}(z_{ki}).$$

This can be written in vector form,

$$\eta_i^{(\theta_m)} = \boldsymbol{\beta}^{(\theta_m)\top}\boldsymbol{b}_i^{(\theta_m)},$$

where

$$\boldsymbol{\beta}^{(\theta_m)} = \left(\beta_1^{(\theta_m)}, \beta_2^{(\theta_m)}, \beta_{1,1}^{(\theta_m)}, \ldots, \beta_{J_k,1}^{(\theta_m)}, \beta_{1,2}^{(\theta_m)}, \ldots, \beta_{J_k,2}^{(\theta_m)}\right)^\top,$$

and

$$\boldsymbol{b}_i^{(\theta_m)} = \left(1,\ \mathbf{1}(g_i=2),\ \mathbf{1}(g_i=1)b_{1,1}^{(\theta_m)}(z_i),\ \ldots\ \mathbf{1}(g_i=1)b_{J_k,1}^{(\theta_m)}(z_i),\ \mathbf{1}(g_i=2)b_{1,2}^{(\theta_m)}(z_i),\ \ldots,\ \mathbf{1}(g_i=2)b_{J_k,2}^{(\theta_m)}(z_i)\right)^\top.$$

For notational simplicity, for the remainder of these appendices we will use $\boldsymbol{\beta}_j$ in place of $\boldsymbol{\beta}^{(\mu_j)}$, $\boldsymbol{\alpha}_j$ in place of $\boldsymbol{\beta}^{(\sigma_j)}$, $\boldsymbol{\tau}$ in place of $\boldsymbol{\beta}^{(\rho)}$, and $\boldsymbol{\kappa}_j$ in place of $\boldsymbol{\beta}^{(p_j)}$.

The parameters of our model can be expressed in the following way:

$$\begin{aligned}
\mu_{ij} &= \exp\left\{\boldsymbol{\beta}_j^\top \boldsymbol{b}^{(\mu_j)}\right\},\\
\sigma_{ij} &= \exp\left\{\boldsymbol{\alpha}_j^\top \boldsymbol{b}^{(\sigma_j)}\right\},\\
\rho_i &= \tanh\left\{\boldsymbol{\tau}^\top \boldsymbol{b}^{(\rho)}\right\},\\
p_{ij} &= \text{sigmoid}\left\{\boldsymbol{\kappa}_j^\top \boldsymbol{b}^{(p_1)}\right\}.
\end{aligned}$$

A.3 Gradient

This section provides the gradient of the log-likelihood. Taking the derivative of ℓ_p with respect to $\boldsymbol{\delta}$ yields:

$$\begin{aligned}
\frac{\partial}{\partial\boldsymbol{\delta}}\ell_p &= \frac{\partial}{\partial\boldsymbol{\delta}}\left(\sum \ell_i - \frac{1}{2}\boldsymbol{\delta}^\top \boldsymbol{S}\boldsymbol{\delta}\right),\\
&= \sum \frac{\partial}{\partial\boldsymbol{\delta}}\ell_i - \boldsymbol{S}\boldsymbol{\delta}.
\end{aligned}$$

So we turn our focus to calculating $\frac{\partial}{\partial\boldsymbol{\delta}}\ell_i$. For notational simplicity, we will drop the subscript i for the remainder of this document. We will also let

$$\begin{aligned}
u_1 &= F_{\text{GA}}(y_1;\ \mu_1, \sigma_1),\\
u_2 &= F_{\text{GA}}(y_2;\ \mu_2, \sigma_2),\\
u_1' &= f_{\text{GA}}(y_1;\ \mu_1, \sigma_1),\\
u_2' &= f_{\text{GA}}(y_2;\ \mu_2, \sigma_2).
\end{aligned}$$

which means the log-likelihood ℓ can be written:

$$\ell = c + \begin{cases}
0 & \text{if } y_{i1} = 0 \text{ and } y_{i2} = 0,\\
\log(u_1') & \text{if } y_{i1} > 0 \text{ and } y_{i2} = 0,\\
\log(u_2') & \text{if } y_{i1} = 0 \text{ and } y_{i2} > 0,\\
\log(c(u_1, u_2; \rho)) + \log(u_1') + \log(u_2') & \text{if } y_{i1} > 0 \text{ and } y_{i2} > 0.
\end{cases}$$

So in order to calculate the first derivatives of ℓ with respect to $\boldsymbol{\beta}_1, \boldsymbol{\beta}_2, \boldsymbol{\alpha}_1, \boldsymbol{\alpha}_2, \boldsymbol{\tau}$, we will need to first calculate the derivatives of $u_1', u_2', u_1, u_2, c(u_1, u_2; \rho)$ with respect to all those coefficients. The first step of that process will be to calculate $\frac{\partial\mu_1}{\partial\boldsymbol{\beta}_1}, \frac{\partial\mu_2}{\partial\boldsymbol{\beta}_2}, \frac{\partial\sigma_1}{\partial\boldsymbol{\alpha}_1}, \frac{\partial\sigma_2}{\partial\boldsymbol{\alpha}_2}, \frac{\partial\rho}{\partial\boldsymbol{\tau}}$, so we will get that out of the way first:

$$\begin{aligned}
\frac{\partial\mu_1}{\partial\boldsymbol{\beta}_1} &= \exp\left\{\boldsymbol{\beta}_1^\top \boldsymbol{b}^{(\mu_1)}\right\}\boldsymbol{b}^{(\mu_1)},\\
\frac{\partial\mu_2}{\partial\boldsymbol{\beta}_2} &= \exp\left\{\boldsymbol{\beta}_2^\top \boldsymbol{b}^{(\mu_2)}\right\}\boldsymbol{b}^{(\mu_2)},\\
\frac{\partial\sigma_1}{\partial\boldsymbol{\alpha}_1} &= \exp\left\{\boldsymbol{\alpha}_1^\top \boldsymbol{b}^{(\sigma_1)}\right\}\boldsymbol{b}^{(\sigma_1)},\\
\frac{\partial\sigma_2}{\partial\boldsymbol{\alpha}_2} &= \exp\left\{\boldsymbol{\alpha}_2^\top \boldsymbol{b}^{(\sigma_2)}\right\}\boldsymbol{b}^{(\sigma_2)},\\
\frac{\partial\rho}{\partial\boldsymbol{\tau}} &= \cosh^2\left\{\boldsymbol{\tau}^\top \boldsymbol{b}^{(\rho)}\right\}\boldsymbol{b}^{(\rho)}.
\end{aligned}$$

Now we can calculate the aforementioned derivatives of $u_1', u_2', u_1, u_2, c(u_1, u_2; \rho)$:

$$\frac{\partial u_j'}{\partial \boldsymbol{\beta}_j} = \frac{\partial f_{\mathrm{GA}}\left(y_j;\ \mu_j, \sigma_j\right)}{\partial \boldsymbol{\beta}_j} = \frac{\partial f_{\mathrm{GA}}\left(y_j;\ \mu_j, \sigma_j\right)}{\partial \mu_j}\frac{\partial \mu_j}{\partial \boldsymbol{\beta}_j} = f_{\mathrm{GA}}\left(y_j;\ \mu_j, \sigma_j\right)\left[\frac{-1}{\mu_j} + \frac{y_j}{\mu_j^2\sigma_j^2}\right]\frac{\partial \mu_j}{\partial \boldsymbol{\beta}_j}.$$

$$\frac{\partial u_j}{\partial \boldsymbol{\beta}_j} = \frac{\partial \int_0^{y_j} f_{\mathrm{GA}}\left(t;\ \mu_j, \sigma_j\right) dt}{\partial \boldsymbol{\beta}_j} = \int_0^{y_j} \frac{\partial f_{\mathrm{GA}}\left(t;\ \mu_j, \sigma_j\right)}{\partial \mu_j}\frac{\partial \mu_j}{\partial \boldsymbol{\beta}_j} dt = \frac{\partial \mu_j}{\partial \boldsymbol{\beta}_j}\int_0^{y_j} f_{\mathrm{GA}}\left(t;\ \mu_j, \sigma_j\right)\left[\frac{-1}{\mu_j} + \frac{t}{\mu_j^2\sigma_j^2}\right] dt.$$

$$\begin{aligned}\frac{\partial u_j'}{\partial \boldsymbol{\alpha}_j} &= \frac{\partial f_{\mathrm{GA}}\left(y_j;\ \mu_j, \sigma_j\right)}{\partial \boldsymbol{\alpha}_j} = \frac{\partial f_{\mathrm{GA}}\left(y_j;\ \mu_j, \sigma_j\right)}{\partial \sigma_j}\frac{\partial \sigma_j}{\partial \boldsymbol{\alpha}_j}, \\ &= f_{\mathrm{GA}}\left(y_j;\ \mu_j, \sigma_j\right)\left[\frac{2}{\sigma_j^3}\log(\mu_j\sigma_j^2) - \frac{1}{\sigma_j^3} + \frac{2}{\sigma_j^3}\psi\left(\frac{1}{\sigma_j^2}\right) - \frac{2\log y_j}{\sigma_j^3} + \frac{2y_j}{\mu_j\sigma_j^3}\right]\frac{\partial \sigma_j}{\partial \boldsymbol{\alpha}_j}.\end{aligned}$$

$$\begin{aligned}\frac{\partial u_j}{\partial \boldsymbol{\alpha}_j} &= \frac{\partial \int_0^{y_j} f_{\mathrm{GA}}\left(t;\ \mu_j, \sigma_j\right) dt}{\partial \boldsymbol{\alpha}_j} = \int_0^{y_j} \frac{\partial f_{\mathrm{GA}}\left(t;\ \mu_j, \sigma_j\right)}{\partial \sigma_j}\frac{\partial \sigma_j}{\partial \boldsymbol{\alpha}_j} dt, \\ &= \frac{\partial \sigma_j}{\partial \boldsymbol{\alpha}_j}\int_0^{y_j} f_{\mathrm{GA}}\left(t;\ \mu_j, \sigma_j\right)\left[\frac{2}{\sigma_j^3}\log(\mu_j\sigma_j^2) - \frac{1}{\sigma_j^3} + \frac{2}{\sigma_j^3}\psi\left(\frac{1}{\sigma_j^2}\right) - \frac{2\log y_j}{\sigma_j^3} + \frac{2y_j}{\mu_j\sigma_j^3}\right] dt.\end{aligned}$$

$$\begin{aligned}\frac{\partial c(u_1, u_2; \rho)}{\partial \boldsymbol{\tau}} &= \frac{\partial c(u_1, u_2; \rho)}{\partial \rho}\frac{\partial \rho}{\partial \boldsymbol{\tau}}, \\ &= -(1-\rho^2)^{\frac{-5}{2}}\left(\rho^3 - \Phi^{-1}(u_1)\Phi^{-1}(u_2)\rho^2 + \rho\Phi^{-1}(u_2)^2 + \rho\Phi^{-1}(u_1)^2 - \rho - \Phi^{-1}(u_1)\Phi^{-1}(u_2)\right), \\ &\quad \times \exp\left\{\frac{1}{2}\frac{\rho(\Phi^{-1}(u_1)^2 + \Phi^{-1}(u_2)^2 - 2x_1\Phi^{-1}(u_2))}{(\rho-1)(\rho+1)}\right\}\frac{\partial \rho}{\partial \boldsymbol{\tau}}.\end{aligned}$$

$$\begin{aligned}\frac{\partial c(u_1, u_2; \rho)}{\partial \boldsymbol{\beta}_1} &= \frac{\partial c(u_1, u_2; \rho)}{\partial u_1}\frac{\partial u_1}{\partial \boldsymbol{\beta}_1}, \\ &= c(u_1, u_2; \rho)\left(-\frac{2\rho^2\Phi^{-1}(u_1)\left(\frac{1}{\phi\{\Phi^{-1}(u_1)\}}\right) - 2\rho\Phi^{-1}(u_2)\left(\frac{1}{\phi\{\Phi^{-1}(u_1)\}}\right)}{2(1-\rho^2)}\right)\frac{\partial u_1}{\partial \boldsymbol{\beta}_1}.\end{aligned}$$

$$\begin{aligned}\frac{\partial c(u_1, u_2; \rho)}{\partial \boldsymbol{\beta}_2} &= \frac{\partial c(u_1, u_2; \rho)}{\partial u_2}\frac{\partial u_2}{\partial \boldsymbol{\beta}_2}, \\ &= c(u_1, u_2; \rho)\left(-\frac{2\rho^2\Phi^{-1}(u_2)\left(\frac{1}{\phi\{\Phi^{-1}(u_2)\}}\right) - 2\rho\Phi^{-1}(u_1)\left(\frac{1}{\phi\{\Phi^{-1}(u_2)\}}\right)}{2(1-\rho^2)}\right)\frac{\partial u_2}{\partial \boldsymbol{\beta}_2}.\end{aligned}$$

$$\frac{\partial c(u_1, u_2; \rho)}{\partial \boldsymbol{\alpha}_j} = \frac{\partial c(u_1, u_2; \rho)}{\partial u_j}\frac{\partial u_j}{\partial \boldsymbol{\alpha}_j}.$$

With these derivatives done, it will be easy to calculate the gradient of our log-likelihood ℓ.

A.3.1 Derivative of log-likelihood with respect to $\boldsymbol{\beta_1}$

$$\frac{\partial \ell}{\partial \boldsymbol{\beta}_1} = \frac{1}{\mathcal{L}}\begin{cases} 0 & \text{if } y_1 = 0 \text{ and } y_2 = 0, \\ \frac{\partial u_1'}{\partial \boldsymbol{\beta}_1} & \text{if } y_1 > 0 \text{ and } y_2 = 0, \\ 0 & \text{if } y_1 = 0 \text{ and } y_2 > 0, \\ \frac{\partial c(u_1, u_2; \rho)}{\partial \boldsymbol{\beta}_1}u_1'u_2' + c(u_1, u_2; \rho)\frac{\partial u_1'}{\partial \boldsymbol{\beta}_1}u_2' & \text{if } y_1 > 0 \text{ and } y_2 > 0. \end{cases}$$

A.3.2 Derivative of log-likelihood with respect to $\boldsymbol{\beta_2}$

$$\frac{\partial \ell}{\partial \boldsymbol{\beta}_2} = \frac{1}{\mathcal{L}}\begin{cases} 0 & \text{if } y_1 = 0 \text{ and } y_2 = 0, \\ 0 & \text{if } y_1 > 0 \text{ and } y_2 = 0, \\ \frac{\partial u_2'}{\partial \boldsymbol{\beta}_2} & \text{if } y_1 = 0 \text{ and } y_2 > 0, \\ \frac{\partial c(u_1, u_2; \rho)}{\partial \boldsymbol{\beta}_2}u_1'u_2' + c(u_1, u_2; \rho)u_1'\frac{\partial u_2'}{\partial \boldsymbol{\beta}_2} & \text{if } y_1 > 0 \text{ and } y_2 > 0. \end{cases}$$

A.3.3 Derivative of log-likelihood with respect to $\boldsymbol{\alpha_1}$

$$\frac{\partial \ell}{\partial \boldsymbol{\alpha}_1} = \frac{1}{\mathcal{L}} \begin{cases} 0 & \text{if } y_1 = 0 \text{ and } y_2 = 0, \\ \frac{\partial u_1'}{\partial \boldsymbol{\alpha}_1} & \text{if } y_1 > 0 \text{ and } y_2 = 0, \\ 0 & \text{if } y_1 = 0 \text{ and } y_2 > 0, \\ \frac{\partial c(u_1,u_2;\rho)}{\partial \boldsymbol{\alpha}_1} u_1' u_2' + c(u_1,u_2;\rho) \frac{\partial u_1'}{\partial \boldsymbol{\alpha}_1} u_2' & \text{if } y_1 > 0 \text{ and } y_2 > 0. \end{cases}$$

A.3.4 Derivative of log-likelihood with respect to $\boldsymbol{\alpha_2}$

$$\frac{\partial \ell}{\partial \boldsymbol{\alpha}_2} = \frac{1}{\mathcal{L}} \begin{cases} 0 & \text{if } y_1 = 0 \text{ and } y_2 = 0, \\ 0 & \text{if } y_1 > 0 \text{ and } y_2 = 0, \\ \frac{\partial u_2'}{\partial \boldsymbol{\alpha}_2} & \text{if } y_1 = 0 \text{ and } y_2 > 0, \\ \frac{\partial c(u_1,u_2;\rho)}{\partial \boldsymbol{\alpha}_2} u_1' u_2' + c(u_1,u_2;\rho) u_1' \frac{\partial u_2'}{\partial \boldsymbol{\alpha}_2} & \text{if } y_1 > 0 \text{ and } y_2 > 0. \end{cases}$$

A.3.5 Derivative of log-likelihood with respect to $\boldsymbol{\tau}$

$$\frac{\partial \ell}{\partial \boldsymbol{\tau}} = \frac{1}{\mathcal{L}} \begin{cases} 0 & \text{if } y_1 = 0 \text{ and } y_2 = 0, \\ 0 & \text{if } y_1 > 0 \text{ and } y_2 = 0, \\ 0 & \text{if } y_1 = 0 \text{ and } y_2 > 0, \\ \frac{\partial c(u_1,u_2;\rho)}{\partial \boldsymbol{\tau}} u_1' u_2' & \text{if } y_1 > 0 \text{ and } y_2 > 0. \end{cases}$$

A.4 Hessian

This section provides the Hessian of the log-likelihood. Taking the second derivative of ℓ_p with respect to $\boldsymbol{\delta}$ yields:

$$\begin{aligned} \frac{\partial^2}{\partial \boldsymbol{\delta}^2} \ell_p &= \frac{\partial^2}{\partial \boldsymbol{\delta}^2} \left(\sum \ell_i - \frac{1}{2} \boldsymbol{\delta}^\top \boldsymbol{S} \boldsymbol{\delta} \right), \\ &= \sum \frac{\partial^2}{\partial \boldsymbol{\delta}^2} \ell_i - \boldsymbol{S}. \end{aligned}$$

So we turn our focus to calculating $\frac{\partial^2}{\partial \boldsymbol{\delta}^2} \ell_i$. To calculate the second derivatives of ℓ with respect to $\boldsymbol{\beta}_1, \boldsymbol{\beta}_2, \boldsymbol{\alpha}_1, \boldsymbol{\alpha}_2, \boldsymbol{\tau}$, we will need to calculate the second derivatives of $u_1', u_2', u_1, u_2, c(u_1,u_2;\rho)$ with respect to all those coefficients. First, we will calculate some of the basic components that will appear in the chain rule calculations throughout:

$$\begin{aligned} \frac{\partial^2 \mu_1}{\partial \boldsymbol{\beta}_1^2} &= \exp\left\{ \boldsymbol{\beta}_1^\top \boldsymbol{b}^{(\mu_1)} \right\} \boldsymbol{b}^{(\mu_1)} \boldsymbol{b}^{(\mu_1)\top}, \\ \frac{\partial^2 \mu_2}{\partial \boldsymbol{\beta}_2^2} &= \exp\left\{ \boldsymbol{\beta}_2^\top \boldsymbol{b}^{(\mu_2)} \right\} \boldsymbol{b}^{(\mu_2)} \boldsymbol{b}^{(\mu_2)\top}, \\ \frac{\partial^2 \sigma_1}{\partial \boldsymbol{\alpha}_1^2} &= \exp\left\{ \boldsymbol{\alpha}_1^\top \boldsymbol{b}^{(\sigma_1)} \right\} \boldsymbol{b}^{(\sigma_1)} \boldsymbol{b}^{(\sigma_1)\top}, \\ \frac{\partial^2 \sigma_2}{\partial \boldsymbol{\alpha}_2^2} &= \exp\left\{ \boldsymbol{\alpha}_2^\top \boldsymbol{b}^{(\sigma_2)} \right\} \boldsymbol{b}^{(\sigma_2)} \boldsymbol{b}^{(\sigma_2)\top}, \\ \frac{\partial^2 \rho}{\partial \boldsymbol{\tau}^2} &= -2\text{sech}^2 \left\{ \boldsymbol{\tau}^\top \boldsymbol{b}^{(\rho)} \right\} \tanh \left\{ \boldsymbol{\tau}^\top \boldsymbol{b}^{(\rho)} \right\} \boldsymbol{b}^{(\rho)} \boldsymbol{b}^{(\rho)\top}. \end{aligned}$$

Now we can calculate the aforementioned second derivatives of $u_1', u_2', u_1, u_2, c(u_1,u_2;\rho)$:

$$\begin{aligned} \frac{\partial^2 u_j'}{\partial \boldsymbol{\beta}_j^2} &= \frac{\partial}{\partial \boldsymbol{\beta}_j} \left[\frac{\partial f_{\text{GA}}\left(y_j;\ \mu_j, \sigma_j\right)}{\partial \boldsymbol{\beta}_j} \right] = \frac{\partial}{\partial \boldsymbol{\beta}_j} \left[\frac{\partial f_{\text{GA}}\left(y_j;\ \mu_j, \sigma_j\right)}{\partial \mu_j} \frac{\partial \mu_j}{\partial \boldsymbol{\beta}_j} \right], \\ &= \left(\frac{\partial}{\partial \boldsymbol{\beta}_j} \frac{\partial f_{\text{GA}}\left(y_j;\ \mu_j, \sigma_j\right)}{\partial \mu_j} \right) \frac{\partial \mu_j}{\partial \boldsymbol{\beta}_j} + \frac{\partial f_{\text{GA}}\left(y_j;\ \mu_j, \sigma_j\right)}{\partial \mu_j} \left(\frac{\partial}{\partial \boldsymbol{\beta}_j} \frac{\partial \mu_j}{\partial \boldsymbol{\beta}_j} \right), \\ &= \frac{\partial^2 f_{\text{GA}}\left(y_j;\ \mu_j, \sigma_j\right)}{\partial \mu_j^2} \left[\frac{\partial \mu_j}{\partial \boldsymbol{\beta}_j} \right]^2 + \frac{\partial f_{\text{GA}}\left(y_j;\ \mu_j, \sigma_j\right)}{\partial \mu_j} \frac{\partial^2 \mu_j}{\partial \boldsymbol{\beta}_j^2}, \\ &= f_{\text{GA}}(y_j; \mu_j, \sigma_j) \left[\frac{2}{\mu_j^2} - \frac{4 y_j}{\mu_j^3 \sigma_j^2} + \frac{y_j^2}{\mu_j^4 \sigma_j^4} \right] \left[\frac{\partial \mu_j}{\partial \boldsymbol{\beta}_j} \right]^2 + \frac{\partial f_{\text{GA}}\left(y_j;\ \mu_j, \sigma_j\right)}{\partial \mu_j} \frac{\partial^2 \mu_j}{\partial \boldsymbol{\beta}_j^2}. \end{aligned}$$

$$\frac{\partial^2 u_j}{\partial \boldsymbol{\beta}_j^2} = \left[\frac{\partial \mu_j}{\partial \boldsymbol{\beta}_j} \right]^2 \int_0^{y_j} f_{\text{GA}}(t; \mu_j, \sigma_j) \left[\frac{2}{\mu_j^2} - \frac{4t}{\mu_j^3 \sigma_j^2} + \frac{t^2}{\mu_j^4 \sigma_j^4} \right] dt + \frac{\partial^2 \mu_j}{\partial \boldsymbol{\beta}_j^2} \int_0^{y_j} \frac{\partial f_{\text{GA}}\left(t;\ \mu_j, \sigma_j\right)}{\partial \mu_j} dt.$$

$$
\begin{aligned}
\frac{\partial^2 u_j'}{\partial \boldsymbol{\alpha}_j^2} &= \frac{\partial}{\partial \boldsymbol{\alpha}_j}\left[\frac{\partial f_{\mathrm{GA}}\left(y_j;\ \mu_j,\sigma_j\right)}{\partial \boldsymbol{\alpha}_j}\right] = \frac{\partial}{\partial \boldsymbol{\alpha}_j}\left[\frac{\partial f_{\mathrm{GA}}\left(y_j;\ \mu_j,\sigma_j\right)}{\partial \sigma_j}\frac{\partial \sigma_j}{\partial \boldsymbol{\alpha}_j}\right],\\
&= \left(\frac{\partial}{\partial \boldsymbol{\alpha}_j}\frac{\partial f_{\mathrm{GA}}\left(y_j;\ \mu_j,\sigma_j\right)}{\partial \sigma_j}\right)\frac{\partial \sigma_j}{\partial \boldsymbol{\alpha}_j} + \frac{\partial f_{\mathrm{GA}}\left(y_j;\ \mu_j,\sigma_j\right)}{\partial \sigma_j}\left(\frac{\partial}{\partial \boldsymbol{\alpha}_j}\frac{\partial \sigma_j}{\partial \boldsymbol{\alpha}_j}\right),\\
&= \frac{\partial^2 f_{\mathrm{GA}}\left(y_j;\ \mu_j,\sigma_j\right)}{\partial \sigma_j^2}\left[\frac{\partial \sigma_j}{\partial \boldsymbol{\alpha}_j}\right]^2 + \frac{\partial f_{\mathrm{GA}}\left(y_j;\ \mu_j,\sigma_j\right)}{\partial \sigma_j}\frac{\partial^2 \sigma_j}{\partial \boldsymbol{\alpha}_j^2},\\
&= f(y_j;\mu_j,\sigma_j)\left[\frac{2}{\sigma_j^4}\left(3\log(\mu_j\sigma_j^2)-1\right) + \frac{3}{\sigma_j^4} + \frac{2}{\sigma_j^4}\left(3\psi\left(\frac{1}{\sigma_j^2}\right) + \frac{1}{\sigma_j^2}\psi'\left(\frac{1}{\sigma_j^2}\right)\right) + \frac{6\log y_j}{\sigma_j^4} + \frac{6y_j}{\mu_j\sigma_j^4}\right]\left[\frac{\partial \sigma_j}{\partial \boldsymbol{\alpha}_j}\right]^2\\
&\quad + \frac{\partial f_{\mathrm{GA}}\left(y_j;\ \mu_j,\sigma_j\right)}{\partial \sigma_j}\frac{\partial^2 \sigma_j}{\partial \boldsymbol{\alpha}_j^2}.
\end{aligned}
$$

$$
\begin{aligned}
\frac{\partial^2 u_j}{\partial \boldsymbol{\alpha}_j^2} &= \left[\frac{\partial \sigma_j}{\partial \boldsymbol{\alpha}_j}\right]^2\int_0^{y_j} f(t;\mu_j,\sigma_j)\left[\frac{2}{\sigma_j^4}\left(3\log(\mu_j\sigma_j^2)-1\right) + \frac{3}{\sigma_j^4} + \frac{2}{\sigma_j^4}\left(3\psi\left(\frac{1}{\sigma_j^2}\right) + \frac{1}{\sigma_j^2}\psi'\left(\frac{1}{\sigma_j^2}\right)\right) + \frac{6\log t}{\sigma_j^4} + \frac{6t}{\mu_j\sigma_j^4}\right]dt\\
&\quad + \frac{\partial^2 \sigma_j}{\partial \boldsymbol{\alpha}_j^2}\int_0^{y_j}\frac{\partial f_{\mathrm{GA}}\left(t;\ \mu_j,\sigma_j\right)}{\partial \sigma_j}dt.
\end{aligned}
$$

$$
\begin{aligned}
\frac{\partial^2 u_j'}{\partial \boldsymbol{\alpha}_j\partial \boldsymbol{\beta}_j} &= \frac{\partial}{\partial \boldsymbol{\alpha}_j}\left[\frac{\partial u_j'}{\partial \boldsymbol{\beta}_j}\right] = \frac{\partial}{\partial \boldsymbol{\alpha}_j}\left[f_{\mathrm{GA}}\left(y_j;\ \mu_j,\sigma_j\right)\left[\frac{-1}{\mu_j} + \frac{y_j}{\mu_j^2\sigma_j^2}\right]\frac{\partial \mu_j}{\partial \boldsymbol{\beta}_j}\right],\\
&= \frac{\partial f_{\mathrm{GA}}\left(y_j;\ \mu_j,\sigma_j\right)}{\partial \boldsymbol{\alpha}_j}\left[\frac{-1}{\mu_j} + \frac{y_j}{\mu_j^2\sigma_j^2}\right]\frac{\partial \mu_j}{\partial \boldsymbol{\beta}_j} + f_{\mathrm{GA}}\left(y_j;\ \mu_j,\sigma_j\right)\frac{\partial}{\partial \sigma_j}\left[\frac{-1}{\mu_j} - \frac{2y_j}{\mu_j^2\sigma_j^3}\right]\frac{\partial \sigma_j}{\partial \boldsymbol{\alpha}_j}\frac{\partial \mu_j}{\partial \boldsymbol{\beta}_j}.
\end{aligned}
$$

$$
\frac{\partial^2 u_j}{\partial \boldsymbol{\alpha}_j\partial \boldsymbol{\beta}_j} = \frac{\partial \mu_j}{\partial \boldsymbol{\beta}_j}\int_0^{y_j}\frac{\partial f_{\mathrm{GA}}\left(t;\ \mu_j,\sigma_j\right)}{\partial \boldsymbol{\alpha}_j}\left[\frac{-1}{\mu_j} + \frac{t}{\mu_j^2\sigma_j^2}\right]dt + \frac{\partial \mu_j}{\partial \boldsymbol{\beta}_j}\int_0^{y_j} f_{\mathrm{GA}}\left(t;\ \mu_j,\sigma_j\right)\left[\frac{-1}{\mu_j} - \frac{2t}{\mu_j^2\sigma_j^3}\right]dt.
$$

$$
\begin{aligned}
\frac{\partial^2 c(u_1,u_2;\rho)}{\partial \boldsymbol{\tau}^2} &= \frac{\partial}{\partial \boldsymbol{\tau}}\left[\frac{\partial c(u_1,u_2;\rho)}{\partial \boldsymbol{\tau}}\right],\\
&= \frac{1}{(1-\rho^2)^5}\Bigg(-(1-\rho^2)^{\frac{5}{2}}\Bigg(3\rho^2 - \frac{\partial\Phi^{-1}(u_1)}{\partial\rho}2\rho^2 - \Phi^{-1}(u_1)\frac{\partial\Phi^{-1}(u_2)}{\partial\rho}\rho^2 - 2\Phi^{-1}(u_1)\Phi^{-1}(u_2)\rho + \Phi^{-1}(u_2)^2\\
&\quad + 2\rho\frac{\partial\Phi^{-1}(u_2)}{\partial\rho}\Phi^{-1}(u_2) + \Phi^{-1}(u_1)^2 + 2\rho\frac{\partial\Phi^{-1}(u_1)}{\partial\rho}\Phi^{-1}(u_1) - 1 - \frac{\partial\Phi^{-1}(u_1)}{\partial\rho} - \Phi^{-1}(u_1)\frac{\partial\Phi^{-1}(u_2)}{\partial\rho} - \Phi^{-1}(u_2)\Bigg)\\
&\quad \times\exp\left(\frac{1}{2}\frac{\rho(\Phi^{-1}(u_1)^2 + \Phi^{-1}(u_2)^2 - 2\Phi^{-1}(u_1)\Phi^{-1}(u_2))}{(\rho-1)(\rho+1)}\right)\\
&\quad + \left(\rho^3 - \Phi^{-1}(u_1)\Phi^{-1}(u_2)\rho^2 + \Phi^{-1}(u_2)^2 + \Phi^{-1}(u_1)^2 - \rho - \Phi^{-1}(u_1)\Phi^{-1}(u_2)\right)\\
&\quad \times\exp\left(\frac{1}{2}\frac{\rho(\Phi^{-1}(u_1)^2 + \Phi^{-1}(u_2)^2 - 2\Phi^{-1}(u_1)\Phi^{-1}(u_2))}{(\rho-1)(\rho+1)}\right)\\
&\quad + \frac{1}{2}\Bigg(\left(\Phi^{-1}(u_1)^2 + \Phi^{-1}(u_2)^2 - 2\Phi^{-1}(u_1)\Phi^{-1}(u_2)\right) + \rho\Bigg(\Phi^{-1}(u_1)^2 + 2\Phi^{-1}(u_1)\frac{\partial\Phi^{-1}(u_1)}{\partial\rho} + \Phi^{-1}(u_2)^2\\
&\quad + 2\Phi^{-1}(u_2)\frac{\partial\Phi^{-1}(u_2)}{\partial\rho}\Bigg)\Bigg)\cdot(\rho-1)(\rho+1) - \left(\rho(\Phi^{-1}(u_1)^2 + \Phi^{-1}(u_2)^2 - 2\Phi^{-1}(u_1)\Phi^{-1}(u_2))\right)\\
&\quad \times\left((\rho-1)^2 + (\rho+1)^2\right)\Big/\left((\rho-1)^2(\rho+1)^2\right)\\
&\quad - \left(\rho^3 - \Phi^{-1}(u_1)\Phi^{-1}(u_2)\rho^2 + \Phi^{-1}(u_2)^2 + \Phi^{-1}(u_1)^2 - \rho - \Phi^{-1}(u_1)\Phi^{-1}(u_2)\right)\\
&\quad \times\exp\left(\frac{1}{2}\frac{\rho(\Phi^{-1}(u_1)^2 + \Phi^{-1}(u_2)^2 - 2\Phi^{-1}(u_1)\Phi^{-1}(u_2))}{(\rho-1)(\rho+1)}\right)(1-\rho^2)^{\frac{3}{2}}5\rho\Bigg).
\end{aligned}
$$

$$\begin{aligned}
\frac{\partial^2 c(u_1,u_2;\rho)}{\partial \boldsymbol{\beta}_1^2} &= \frac{\partial}{\partial \boldsymbol{\beta}_1}\left[\frac{\partial c(u_1,u_2;\rho)}{\partial \boldsymbol{\beta}_1}\right] = \left[\frac{\partial}{\partial u_1}\left(\frac{\partial c(u_1,u_2;\rho)}{\partial u_1}\frac{\partial u_1}{\partial \boldsymbol{\beta}_1}\right)\right]\frac{\partial u_1}{\partial \boldsymbol{\beta}_1},\\
&= \left[\frac{\partial^2 c(u_1,u_2;\rho)}{\partial u_1^2}\frac{\partial u_1}{\partial \boldsymbol{\beta}_1} + \frac{\partial c(u_1,u_2;\rho)}{\partial u_1}\frac{\partial^2 u_1}{\partial \boldsymbol{\beta}_1^2}\left(\frac{\partial u_1}{\partial \boldsymbol{\beta}_1}\right)^{-1}\right]\frac{\partial u_1}{\partial \boldsymbol{\beta}_1},\\
&= \frac{\partial^2 c(u_1,u_2;\rho)}{\partial u_1^2}\left[\frac{\partial u_1}{\partial \boldsymbol{\beta}_1}\right]^2 + \frac{\partial c(u_1,u_2;\rho)}{\partial u_1}\frac{\partial^2 u_1}{\partial \boldsymbol{\beta}_1^2},\\
&= \left\{\frac{\partial c(u_1,u_2;\rho)}{\partial u_1}\left(\frac{-2\rho^2\Phi^{-1}(u_1)\frac{1}{\phi\{\Phi^{-1}(u_1)\}} - 2\rho\Phi^{-1}(u_2)\frac{1}{\phi\{\Phi^{-1}(u_1)\}}}{2(1-\rho^2)}\right)\right.\\
&\quad \left. + c(u_1,u_2;\rho)\left(\frac{-2\rho^2\left(\left(\frac{1}{\phi\{\Phi^{-1}(u_1)\}}\right)^2 + \Phi^{-1}(u_1)\frac{\partial^2\Phi^{-1}(u_1)}{\partial u_1^2}\right) - 2\rho\Phi^{-1}(u_2)\frac{\partial^2\Phi^{-1}(u_1)}{\partial u_1^2}}{2(1-\rho^2)}\right)\right\}\left[\frac{\partial u_1}{\partial \boldsymbol{\beta}_1}\right]^2\\
&\quad + \frac{\partial c(u_1,u_2;\rho)}{\partial u_1}\frac{\partial^2 u_1}{\partial \boldsymbol{\beta}_1^2}.
\end{aligned}$$

$$\begin{aligned}
\frac{\partial^2 c(u_1,u_2;\rho)}{\partial \boldsymbol{\beta}_2^2} &= \frac{\partial}{\partial \boldsymbol{\beta}_2}\left[\frac{\partial c(u_1,u_2;\rho)}{\partial \boldsymbol{\beta}_2}\right] = \left[\frac{\partial}{\partial u_2}\left(\frac{\partial c(u_1,u_2;\rho)}{\partial u_2}\frac{\partial u_2}{\partial \boldsymbol{\beta}_2}\right)\right]\frac{\partial u_2}{\partial \boldsymbol{\beta}_2},\\
&= \left[\frac{\partial^2 c(u_1,u_2;\rho)}{\partial u_2^2}\frac{\partial u_2}{\partial \boldsymbol{\beta}_2} + \frac{\partial c(u_1,u_2;\rho)}{\partial u_2}\frac{\partial^2 u_2}{\partial \boldsymbol{\beta}_2^2}\left(\frac{\partial u_2}{\partial \boldsymbol{\beta}_2}\right)^{-1}\right]\frac{\partial u_2}{\partial \boldsymbol{\beta}_2},\\
&= \frac{\partial^2 c(u_1,u_2;\rho)}{\partial u_2^2}\left[\frac{\partial u_2}{\partial \boldsymbol{\beta}_2}\right]^2 + \frac{\partial c(u_1,u_2;\rho)}{\partial u_2}\frac{\partial^2 u_2}{\partial \boldsymbol{\beta}_2^2},\\
&= \left\{\frac{\partial c(u_1,u_2;\rho)}{\partial u_2}\left(\frac{-2\rho^2\Phi^{-1}(u_2)\frac{1}{\phi\{\Phi^{-1}(u_2)\}} - 2\rho\Phi^{-1}(u_1)\frac{1}{\phi\{\Phi^{-1}(u_2)\}}}{2(1-\rho^2)}\right)\right.\\
&\quad \left. + c(u_1,u_2;\rho)\left(\frac{-2\rho^2\left(\left(\frac{1}{\phi\{\Phi^{-1}(u_2)\}}\right)^2 + \Phi^{-1}(u_2)\frac{\partial^2\Phi^{-1}(u_2)}{\partial u_2^2}\right) - 2\rho\Phi^{-1}(u_1)\frac{\partial^2\Phi^{-1}(u_2)}{\partial u_2^2}}{2(1-\rho^2)}\right)\right\}\left[\frac{\partial u_2}{\partial \boldsymbol{\beta}_2}\right]^2\\
&\quad + \frac{\partial c(u_1,u_2;\rho)}{\partial u_2}\frac{\partial^2 u_2}{\partial \boldsymbol{\beta}_2^2}.
\end{aligned}$$

$$\begin{aligned}
\frac{\partial^2 c(u_1,u_2;\rho)}{\partial \boldsymbol{\alpha}_j^2} &= \frac{\partial}{\partial \boldsymbol{\alpha}_j}\left[\frac{\partial c(u_1,u_2;\rho)}{\partial \boldsymbol{\alpha}_j}\right] = \left[\frac{\partial}{\partial u_j}\left(\frac{\partial c(u_1,u_2;\rho)}{\partial u_j}\frac{\partial u_j}{\partial \boldsymbol{\alpha}_j}\right)\right]\frac{\partial u_j}{\partial \boldsymbol{\alpha}_j},\\
&= \left[\frac{\partial^2 c(u_1,u_2;\rho)}{\partial u_j^2}\frac{\partial u_j}{\partial \boldsymbol{\alpha}_j} + \frac{\partial c(u_1,u_2;\rho)}{\partial u_j}\frac{\partial^2 u_j}{\partial \boldsymbol{\alpha}_j^2}\left(\frac{\partial u_j}{\partial \boldsymbol{\alpha}_j}\right)^{-1}\right]\frac{\partial u_j}{\partial \boldsymbol{\alpha}_j},\\
&= \frac{\partial^2 c(u_1,u_2;\rho)}{\partial u_j^2}\left[\frac{\partial u_j}{\partial \boldsymbol{\alpha}_j}\right]^2 + \frac{\partial c(u_1,u_2;\rho)}{\partial u_j}\frac{\partial^2 u_j}{\partial \boldsymbol{\alpha}_j^2}.
\end{aligned}$$

$$\begin{aligned}
\frac{\partial^2 c(u_1,u_2;\rho)}{\partial \boldsymbol{\beta}_1\partial \boldsymbol{\beta}_2} &= \frac{\partial}{\partial \boldsymbol{\beta}_1}\left[\frac{\partial c(u_1,u_2;\rho)}{\partial \boldsymbol{\beta}_2}\right] = \left[\frac{\partial}{\partial u_1}\left(\frac{\partial c(u_1,u_2;\rho)}{\partial u_2}\frac{\partial u_2}{\partial \boldsymbol{\beta}_2}\right)\right]\frac{\partial u_1}{\partial \boldsymbol{\beta}_1},\\
&= \left[\frac{\partial^2 c(u_1,u_2;\rho)}{\partial u_1\partial u_2}\frac{\partial u_2}{\partial \boldsymbol{\beta}_2} + \frac{\partial c(u_1,u_2;\rho)}{\partial u_2}\left(\frac{\partial}{\partial u_1}\frac{\partial u_2}{\partial \boldsymbol{\beta}_2}\right)\right]\frac{\partial u_1}{\partial \boldsymbol{\beta}_1},\\
&= \frac{\partial^2 c(u_1,u_2;\rho)}{\partial u_1\partial u_2}\frac{\partial u_2}{\partial \boldsymbol{\beta}_2}\frac{\partial u_1}{\partial \boldsymbol{\beta}_1},\\
&= c(u_1,u_2;\rho)\left[\left(\frac{2\rho^2\Phi^{-1}(u_2)\frac{1}{\phi\{\Phi^{-1}(u_2)\}} - 2\rho\Phi^{-1}(u_1)\frac{1}{\phi\{\Phi^{-1}(u_2)\}}}{2(1-\rho^2)}\right)\left(\frac{\left(2\rho^2\Phi^{-1}(u_1)\frac{1}{\phi\{\Phi^{-1}(u_1)\}} - 2\rho\Phi^{-1}(u_2)\frac{1}{\phi\{\Phi^{-1}(u_1)\}}\right)}{2(1-\rho^2)}\right)\right.\\
&\quad \left. + \frac{\rho\frac{1}{\phi\{\Phi^{-1}(u_1)\}}\frac{1}{\phi\{\Phi^{-1}(u_2)\}}}{1-\rho^2}\right]\frac{\partial u_2}{\partial \boldsymbol{\beta}_2}\frac{\partial u_1}{\partial \boldsymbol{\beta}_1}.
\end{aligned}$$

$$\frac{\partial^2 c(u_1,u_2;\rho)}{\partial \boldsymbol{\alpha}_1 \partial \boldsymbol{\alpha}_2} = \frac{\partial}{\partial \boldsymbol{\alpha}_1}\left[\frac{\partial c(u_1,u_2;\rho)}{\partial \boldsymbol{\alpha}_2}\right] = \left[\frac{\partial}{\partial u_1}\left(\frac{\partial c(u_1,u_2;\rho)}{\partial u_2}\frac{\partial u_2}{\partial \boldsymbol{\alpha}_2}\right)\right]\frac{\partial u_1}{\partial \boldsymbol{\alpha}_1},$$
$$= \left[\frac{\partial^2 c(u_1,u_2;\rho)}{\partial u_1 \partial u_2}\frac{\partial u_2}{\partial \boldsymbol{\alpha}_2} + \frac{\partial c(u_1,u_2;\rho)}{\partial u_2}\left(\frac{\partial}{\partial u_1}\frac{\partial u_2}{\partial \boldsymbol{\alpha}_2}\right)\right]\frac{\partial u_1}{\partial \boldsymbol{\alpha}_1},$$
$$= \frac{\partial^2 c(u_1,u_2;\rho)}{\partial u_1 \partial u_2}\frac{\partial u_2}{\partial \boldsymbol{\alpha}_2}\frac{\partial u_1}{\partial \boldsymbol{\alpha}_1}.$$

$$\frac{\partial^2 c(u_1,u_2;\rho)}{\partial \boldsymbol{\alpha}_j \partial \boldsymbol{\beta}_j} = \frac{\partial}{\partial \boldsymbol{\alpha}_j}\left[\frac{\partial c(u_1,u_2;\rho)}{\partial \boldsymbol{\beta}_j}\right] = \left[\frac{\partial}{\partial u_j}\left(\frac{\partial c(u_1,u_2;\rho)}{\partial u_j}\frac{\partial u_j}{\partial \boldsymbol{\beta}_j}\right)\right]\frac{\partial u_j}{\partial \boldsymbol{\alpha}_j},$$
$$= \left[\frac{\partial^2 c(u_1,u_2;\rho)}{\partial u_j^2}\frac{\partial u_j}{\partial \boldsymbol{\beta}_j} + \frac{\partial c(u_1,u_2;\rho)}{\partial u_j}\frac{\partial^2 u_j}{\partial \boldsymbol{\alpha}_j \partial \boldsymbol{\beta}_j}\left(\frac{\partial u_j}{\partial \boldsymbol{\beta}_j}\right)^{-1}\right]\frac{\partial u_j}{\partial \boldsymbol{\alpha}_j},$$
$$= \frac{\partial^2 c(u_1,u_2;\rho)}{\partial u_j^2}\frac{\partial u_j}{\partial \boldsymbol{\beta}_j}\frac{\partial u_j}{\partial \boldsymbol{\alpha}_j} + \frac{\partial c(u_1,u_2;\rho)}{\partial u_j}\frac{\partial^2 u_j}{\partial \boldsymbol{\alpha}_j \partial \boldsymbol{\beta}_j}\left(\frac{\partial u_j}{\partial \boldsymbol{\beta}_j}\right)^{-1}\frac{\partial u_j}{\partial \boldsymbol{\alpha}_j}.$$

$$\frac{\partial^2 c(u_1,u_2;\rho)}{\partial \boldsymbol{\alpha}_2 \partial \boldsymbol{\beta}_1} = \frac{\partial}{\partial \boldsymbol{\alpha}_2}\left[\frac{\partial c(u_1,u_2;\rho)}{\partial \boldsymbol{\beta}_1}\right] = \left[\frac{\partial}{\partial u_2}\left(\frac{\partial c(u_1,u_2;\rho)}{\partial u_1}\frac{\partial u_1}{\partial \boldsymbol{\beta}_1}\right)\right]\frac{\partial u_2}{\partial \boldsymbol{\alpha}_2},$$
$$= \left[\frac{\partial^2 c(u_1,u_2;\rho)}{\partial u_2 \partial u_1}\frac{\partial u_1}{\partial \boldsymbol{\beta}_1} + \frac{\partial c(u_1,u_2;\rho)}{\partial u_1}\left(\frac{\partial}{\partial u_2}\frac{\partial u_1}{\partial \boldsymbol{\beta}_1}\right)\right]\frac{\partial u_2}{\partial \boldsymbol{\alpha}_2},$$
$$= \frac{\partial^2 c(u_1,u_2;\rho)}{\partial u_2 \partial u_1}\frac{\partial u_1}{\partial \boldsymbol{\beta}_1}\frac{\partial u_2}{\partial \boldsymbol{\alpha}_2}.$$

$$\frac{\partial^2 c(u_1,u_2;\rho)}{\partial \boldsymbol{\alpha}_1 \partial \boldsymbol{\beta}_2} = \frac{\partial}{\partial \boldsymbol{\alpha}_1}\left[\frac{\partial c(u_1,u_2;\rho)}{\partial \boldsymbol{\beta}_2}\right] = \left[\frac{\partial}{\partial u_1}\left(\frac{\partial c(u_1,u_2;\rho)}{\partial u_2}\frac{\partial u_2}{\partial \boldsymbol{\beta}_2}\right)\right]\frac{\partial u_1}{\partial \boldsymbol{\alpha}_1},$$
$$= \left[\frac{\partial^2 c(u_1,u_2;\rho)}{\partial u_1 \partial u_2}\frac{\partial u_2}{\partial \boldsymbol{\beta}_2} + \frac{\partial c(u_1,u_2;\rho)}{\partial u_2}\left(\frac{\partial}{\partial u_1}\frac{\partial u_2}{\partial \boldsymbol{\beta}_2}\right)\right]\frac{\partial u_1}{\partial \boldsymbol{\alpha}_1},$$
$$= \frac{\partial^2 c(u_1,u_2;\rho)}{\partial u_1 \partial u_2}\frac{\partial u_2}{\partial \boldsymbol{\beta}_2}\frac{\partial u_1}{\partial \boldsymbol{\alpha}_1}.$$

$$\frac{\partial^2 c(u_1,u_2;\rho)}{\partial \boldsymbol{\tau} \partial \boldsymbol{\beta}_j} = \frac{\partial}{\partial \boldsymbol{\tau}}\left[\frac{\partial c(u_1,u_2;\rho)}{\partial \boldsymbol{\beta}_j}\right] = \left[\frac{\partial}{\partial \rho}\left(\frac{\partial c(u_1,u_2;\rho)}{\partial u_j}\frac{\partial u_j}{\partial \boldsymbol{\beta}_j}\right)\right]\frac{\partial \rho}{\partial \boldsymbol{\tau}},$$
$$= \left[\frac{\partial^2 c(u_1,u_2;\rho)}{\partial \rho \partial u_j}\frac{\partial u_j}{\partial \boldsymbol{\beta}_j} + \frac{\partial c(u_1,u_2;\rho)}{\partial u_j}\left(\frac{\partial}{\partial \rho}\frac{\partial u_j}{\partial \boldsymbol{\beta}_j}\right)\right]\frac{\partial \rho}{\partial \boldsymbol{\tau}},$$
$$= \frac{\partial^2 c(u_1,u_2;\rho)}{\partial \rho \partial u_j}\frac{\partial u_j}{\partial \boldsymbol{\beta}_j}\frac{\partial \rho}{\partial \boldsymbol{\tau}},$$
$$= \left[-\frac{1}{(1-\rho^2)^{\frac{5}{2}}}\left(-\frac{1}{\phi\left\{\Phi^{-1}(u_1)\right\}}\Phi^{-1}(u_2)\rho^2 + 2\rho\Phi^{-1}(u_1)\frac{1}{\phi\left\{\Phi^{-1}(u_1)\right\}} - \frac{1}{\phi\left\{\Phi^{-1}(u_1)\right\}}\right)\right.$$
$$\times \exp\left\{\frac{1}{2}\frac{\rho\left(\rho\left\{\Phi^{-1}(u_1)\right\}^2 + \rho\left\{\Phi^{-1}(u_2)\right\}^2 - 2\Phi^{-1}(u_1)\Phi^{-1}(u_2)\right)}{(\rho-1)(\rho+1)}\right\}$$
$$\left. + \frac{\partial c(u_1,u_2;\rho)}{\partial \rho}\frac{\rho}{2(\rho-1)(\rho+1)}\left(2\Phi^{-1}(u_1)\frac{1}{\phi\left\{\Phi^{-1}(u_1)\right\}} - 2\frac{1}{\phi\left\{\Phi^{-1}(u_1)\right\}}\Phi^{-1}(u_2)\right)\right]\frac{\partial u_j}{\partial \boldsymbol{\beta}_j}\frac{\partial \rho}{\partial \boldsymbol{\tau}}.$$

$$\frac{\partial^2 c(u_1,u_2;\rho)}{\partial \boldsymbol{\tau} \partial \boldsymbol{\alpha}_j} = \frac{\partial}{\partial \boldsymbol{\tau}}\left[\frac{\partial c(u_1,u_2;\rho)}{\partial \boldsymbol{\alpha}_j}\right] = \left[\frac{\partial}{\partial \rho}\left(\frac{\partial c(u_1,u_2;\rho)}{\partial u_j}\frac{\partial u_j}{\partial \boldsymbol{\alpha}_j}\right)\right]\frac{\partial \rho}{\partial \boldsymbol{\tau}},$$
$$= \left[\frac{\partial^2 c(u_1,u_2;\rho)}{\partial \rho \partial u_j}\frac{\partial u_j}{\partial \boldsymbol{\alpha}_j} + \frac{\partial c(u_1,u_2;\rho)}{\partial u_j}\left(\frac{\partial}{\partial \rho}\frac{\partial u_j}{\partial \boldsymbol{\alpha}_j}\right)\right]\frac{\partial \rho}{\partial \boldsymbol{\tau}},$$
$$= \frac{\partial^2 c(u_1,u_2;\rho)}{\partial \rho \partial u_j}\frac{\partial u_j}{\partial \boldsymbol{\alpha}_j}\frac{\partial \rho}{\partial \boldsymbol{\tau}}.$$

With these second derivatives done, it will be easy to calculate the Hessian of our log-likelihood ℓ.

A.4.1 2nd derivative with respect to $\boldsymbol{\beta}_1$

$$\frac{\partial^2 \ell}{\partial \boldsymbol{\beta}_1^2} = \frac{\partial}{\partial \boldsymbol{\beta}_1}\left(\frac{1}{\mathcal{L}}\frac{\partial \mathcal{L}}{\partial \boldsymbol{\beta}_1}\right) = \left(\frac{\partial}{\partial \boldsymbol{\beta}_1}\frac{1}{\mathcal{L}}\right)\frac{\partial \mathcal{L}}{\partial \boldsymbol{\beta}_1} + \frac{1}{\mathcal{L}}\frac{\partial^2 \mathcal{L}}{\partial \boldsymbol{\beta}_1^2},$$

$$= \frac{-1}{\mathcal{L}^2}\left[\frac{\partial \mathcal{L}}{\partial \boldsymbol{\beta}_1}\right]^2 + \frac{1}{\mathcal{L}}\left(\frac{\partial}{\partial \boldsymbol{\beta}_1}\begin{cases} 0 & \text{if } y_1 = 0 \text{ and } y_2 = 0 \\ \frac{\partial u_1'}{\partial \boldsymbol{\beta}_1} & \text{if } y_1 > 0 \text{ and } y_2 = 0 \\ 0 & \text{if } y_1 = 0 \text{ and } y_2 > 0 \\ \frac{\partial c(u_1,u_2;\rho)}{\partial \boldsymbol{\beta}_1} u_1' u_2' + c(u_1,u_2;\rho)\frac{\partial u_1'}{\partial \boldsymbol{\beta}_1} u_2' & \text{if } y_1 > 0 \text{ and } y_2 > 0 \end{cases}\right),$$

$$= \frac{-1}{\mathcal{L}^2}\left[\frac{\partial \mathcal{L}}{\partial \boldsymbol{\beta}_1}\right]^2 + \frac{1}{\mathcal{L}}\begin{cases} 0 & \text{if } y_1 = 0 \text{ and } y_2 = 0 \\ \frac{\partial^2 u_1'}{\partial \boldsymbol{\beta}_1^2} & \text{if } y_1 > 0 \text{ and } y_2 = 0 \\ 0 & \text{if } y_1 = 0 \text{ and } y_2 > 0 \\ \frac{\partial^2 c(u_1,u_2;\rho)}{\partial \boldsymbol{\beta}_1^2} u_1' u_2' + \frac{\partial c(u_1,u_2;\rho)}{\partial \boldsymbol{\beta}_1}\frac{\partial u_1'}{\partial \boldsymbol{\beta}_1} u_2' + \frac{\partial c(u_1,u_2;\rho)}{\partial \boldsymbol{\beta}_1}\frac{\partial u_1'}{\partial \boldsymbol{\beta}_1} u_2' + c(u_1,u_2;\rho)\frac{\partial^2 u_1'}{\partial \boldsymbol{\beta}_1^2} u_2' & \text{if } y_1 > 0 \text{ and } y_2 > 0 \end{cases}.$$

A.4.2 2nd derivative with respect to $\boldsymbol{\beta}_2$

$$\frac{\partial^2 \ell}{\partial \boldsymbol{\beta}_2^2} = \frac{\partial}{\partial \boldsymbol{\beta}_2}\left(\frac{1}{\mathcal{L}}\frac{\partial \mathcal{L}}{\partial \boldsymbol{\beta}_2}\right) = \left(\frac{\partial}{\partial \boldsymbol{\beta}_2}\frac{1}{\mathcal{L}}\right)\frac{\partial \mathcal{L}}{\partial \boldsymbol{\beta}_2} + \frac{1}{\mathcal{L}}\frac{\partial^2 \mathcal{L}}{\partial \boldsymbol{\beta}_2^2},$$

$$= \frac{-1}{\mathcal{L}^2}\left[\frac{\partial \mathcal{L}}{\partial \boldsymbol{\beta}_2}\right]^2 + \frac{1}{\mathcal{L}}\left(\frac{\partial}{\partial \boldsymbol{\beta}_2}\begin{cases} 0 & \text{if } y_1 = 0 \text{ and } y_2 = 0 \\ 0 & \text{if } y_1 > 0 \text{ and } y_2 = 0 \\ \frac{\partial u_2'}{\partial \boldsymbol{\beta}_2} & \text{if } y_1 = 0 \text{ and } y_2 > 0 \\ \frac{\partial c(u_1,u_2;\rho)}{\partial \boldsymbol{\beta}_2} u_1' u_2' + c(u_1,u_2;\rho) u_1' \frac{\partial u_2'}{\partial \boldsymbol{\beta}_2} & \text{if } y_1 > 0 \text{ and } y_2 > 0 \end{cases}\right),$$

$$= \frac{-1}{\mathcal{L}^2}\left[\frac{\partial \mathcal{L}}{\partial \boldsymbol{\beta}_2}\right]^2 + \frac{1}{\mathcal{L}}\begin{cases} 0 & \text{if } y_1 = 0 \text{ and } y_2 = 0 \\ 0 & \text{if } y_1 > 0 \text{ and } y_2 = 0 \\ \frac{\partial^2 u_2'}{\partial \boldsymbol{\beta}_2^2} & \text{if } y_1 = 0 \text{ and } y_2 > 0 \\ \frac{\partial^2 c(u_1,u_2;\rho)}{\partial \boldsymbol{\beta}_2^2} u_1' u_2' + \frac{\partial c(u_1,u_2;\rho)}{\partial \boldsymbol{\beta}_2} u_1' \frac{\partial u_2'}{\partial \boldsymbol{\beta}_2} + \frac{\partial c(u_1,u_2;\rho)}{\partial \boldsymbol{\beta}_2} u_1' \frac{\partial u_2'}{\partial \boldsymbol{\beta}_2} + c(u_1,u_2;\rho) u_1' \frac{\partial^2 u_2'}{\partial \boldsymbol{\beta}_2^2} & \text{if } y_1 > 0 \text{ and } y_2 > 0 \end{cases}.$$

A.4.3 2nd derivative with respect to $\boldsymbol{\alpha}_1$

$$\frac{\partial^2 \ell}{\partial \boldsymbol{\alpha}_1^2} = \frac{\partial}{\partial \boldsymbol{\alpha}_1}\left(\frac{1}{\mathcal{L}}\frac{\partial \mathcal{L}}{\partial \boldsymbol{\alpha}_1}\right) = \left(\frac{\partial}{\partial \boldsymbol{\alpha}_1}\frac{1}{\mathcal{L}}\right)\frac{\partial \mathcal{L}}{\partial \boldsymbol{\alpha}_1} + \frac{1}{\mathcal{L}}\frac{\partial^2 \mathcal{L}}{\partial \boldsymbol{\alpha}_1^2},$$

$$= \frac{-1}{\mathcal{L}^2}\left[\frac{\partial \mathcal{L}}{\partial \boldsymbol{\alpha}_1}\right]^2 + \frac{1}{\mathcal{L}}\left(\frac{\partial}{\partial \boldsymbol{\alpha}_1}\begin{cases} 0 & \text{if } y_1 = 0 \text{ and } y_2 = 0 \\ \frac{\partial u_1'}{\partial \boldsymbol{\alpha}_1} & \text{if } y_1 > 0 \text{ and } y_2 = 0 \\ 0 & \text{if } y_1 = 0 \text{ and } y_2 > 0 \\ \frac{\partial c(u_1,u_2;\rho)}{\partial \boldsymbol{\alpha}_1} u_1' u_2' + c(u_1,u_2;\rho)\frac{\partial u_1'}{\partial \boldsymbol{\alpha}_1} u_2' & \text{if } y_1 > 0 \text{ and } y_2 > 0 \end{cases}\right),$$

$$= \frac{-1}{\mathcal{L}^2}\left[\frac{\partial \mathcal{L}}{\partial \boldsymbol{\alpha}_1}\right]^2 + \frac{1}{\mathcal{L}}\begin{cases} 0 & \text{if } y_1 = 0 \text{ and } y_2 = 0 \\ \frac{\partial^2 u_1'}{\partial \boldsymbol{\alpha}_1^2} & \text{if } y_1 > 0 \text{ and } y_2 = 0 \\ 0 & \text{if } y_1 = 0 \text{ and } y_2 > 0 \\ \frac{\partial^2 c(u_1,u_2;\rho)}{\partial \boldsymbol{\alpha}_1^2} u_1' u_2' + \frac{\partial c(u_1,u_2;\rho)}{\partial \boldsymbol{\alpha}_1}\frac{\partial u_1'}{\partial \boldsymbol{\alpha}_1} u_2' + \frac{\partial c(u_1,u_2;\rho)}{\partial \boldsymbol{\alpha}_1}\frac{\partial u_1'}{\partial \boldsymbol{\alpha}_1} u_2' + c(u_1,u_2;\rho)\frac{\partial^2 u_1'}{\partial \boldsymbol{\alpha}_1^2} u_2' & \text{if } y_1 > 0 \text{ and } y_2 > 0 \end{cases}.$$

A.4.4 2nd derivative with respect to $\boldsymbol{\alpha}_2$

$$\frac{\partial^2 \ell}{\partial \boldsymbol{\alpha}_2^2} = \frac{\partial}{\partial \boldsymbol{\alpha}_2}\left(\frac{1}{\mathcal{L}}\frac{\partial \mathcal{L}}{\partial \boldsymbol{\alpha}_2}\right) = \left(\frac{\partial}{\partial \boldsymbol{\alpha}_2}\frac{1}{\mathcal{L}}\right)\frac{\partial \mathcal{L}}{\partial \boldsymbol{\alpha}_2} + \frac{1}{\mathcal{L}}\frac{\partial^2 \mathcal{L}}{\partial \boldsymbol{\alpha}_2^2},$$

$$= \frac{-1}{\mathcal{L}^2}\left[\frac{\partial \mathcal{L}}{\partial \boldsymbol{\alpha}_2}\right]^2 + \frac{1}{\mathcal{L}}\left(\frac{\partial}{\partial \boldsymbol{\alpha}_2}\begin{cases} 0 & \text{if } y_1 = 0 \text{ and } y_2 = 0 \\ 0 & \text{if } y_1 > 0 \text{ and } y_2 = 0 \\ \frac{\partial u_2'}{\partial \boldsymbol{\alpha}_2} & \text{if } y_1 = 0 \text{ and } y_2 > 0 \\ \frac{\partial c(u_1,u_2;\rho)}{\partial \boldsymbol{\alpha}_2} u_1' u_2' + c(u_1,u_2;\rho) u_1' \frac{\partial u_2'}{\partial \boldsymbol{\alpha}_2} & \text{if } y_1 > 0 \text{ and } y_2 > 0 \end{cases}\right),$$

$$= \frac{-1}{\mathcal{L}^2}\left[\frac{\partial \mathcal{L}}{\partial \boldsymbol{\alpha}_2}\right]^2 + \frac{1}{\mathcal{L}}\begin{cases} 0 & \text{if } y_1 = 0 \text{ and } y_2 = 0 \\ 0 & \text{if } y_1 > 0 \text{ and } y_2 = 0 \\ \frac{\partial^2 u_2'}{\partial \boldsymbol{\alpha}_2^2} & \text{if } y_1 = 0 \text{ and } y_2 > 0 \\ \frac{\partial^2 c(u_1,u_2;\rho)}{\partial \boldsymbol{\alpha}_2^2} u_1' u_2' + \frac{\partial c(u_1,u_2;\rho)}{\partial \boldsymbol{\alpha}_2} u_1' \frac{\partial u_2'}{\partial \boldsymbol{\alpha}_2} + \frac{\partial c(u_1,u_2;\rho)}{\partial \boldsymbol{\alpha}_2} u_1' \frac{\partial u_2'}{\partial \boldsymbol{\alpha}_2} + c(u_1,u_2;\rho) u_1' \frac{\partial^2 u_2'}{\partial \boldsymbol{\alpha}_2^2} & \text{if } y_1 > 0 \text{ and } y_2 > 0 \end{cases}.$$

A.4.5 2nd derivative with respect to τ

$$\frac{\partial^2 \ell}{\partial \tau^2} = \frac{\partial}{\partial \tau}\left(\frac{1}{\mathcal{L}}\frac{\partial \mathcal{L}}{\partial \tau}\right) = \left(\frac{\partial}{\partial \tau}\frac{1}{\mathcal{L}}\right)\frac{\partial \mathcal{L}}{\partial \tau} + \frac{1}{\mathcal{L}}\frac{\partial^2 \mathcal{L}}{\partial \tau^2},$$

$$= \frac{-1}{\mathcal{L}^2}\left[\frac{\partial \mathcal{L}}{\partial \tau}\right]^2 + \frac{1}{\mathcal{L}}\left(\frac{\partial}{\partial \tau}\begin{cases} 0 & \text{if } y_1 = 0 \text{ and } y_2 = 0 \\ 0 & \text{if } y_1 > 0 \text{ and } y_2 = 0 \\ 0 & \text{if } y_1 = 0 \text{ and } y_2 > 0 \\ \frac{\partial c(u_1,u_2;\rho)}{\partial \tau} u_1' u_2' & \text{if } y_1 > 0 \text{ and } y_2 > 0 \end{cases}\right),$$

$$= \frac{-1}{\mathcal{L}^2}\left[\frac{\partial \mathcal{L}}{\partial \tau}\right]^2 + \frac{1}{\mathcal{L}}\begin{cases} 0 & \text{if } y_1 = 0 \text{ and } y_2 = 0 \\ 0 & \text{if } y_1 > 0 \text{ and } y_2 = 0 \\ 0 & \text{if } y_1 = 0 \text{ and } y_2 > 0 \\ \frac{\partial^2 c(u_1,u_2;\rho)}{\partial \tau^2} u_1' u_2' & \text{if } y_1 > 0 \text{ and } y_2 > 0 \end{cases}.$$

A.4.6 Mixed derivative with respect to β_1 and β_2

$$\frac{\partial^2 \ell}{\partial \beta_1 \partial \beta_2} = \frac{\partial}{\partial \beta_1}\left(\frac{1}{\mathcal{L}}\frac{\partial \mathcal{L}}{\partial \beta_2}\right) = \left(\frac{\partial}{\partial \beta_1}\frac{1}{\mathcal{L}}\right)\frac{\partial \mathcal{L}}{\partial \beta_2} + \frac{1}{\mathcal{L}}\frac{\partial^2 \mathcal{L}}{\partial \beta_1 \partial \beta_2},$$

$$= \frac{-1}{\mathcal{L}^2}\frac{\partial \mathcal{L}}{\partial \beta_1}\frac{\partial \mathcal{L}}{\partial \beta_2} + \frac{1}{\mathcal{L}}\left(\frac{\partial}{\partial \beta_1}\begin{cases} 0 & \text{if } y_1 = 0 \text{ and } y_2 = 0 \\ 0 & \text{if } y_1 > 0 \text{ and } y_2 = 0 \\ \frac{\partial u_2'}{\partial \beta_2} & \text{if } y_1 = 0 \text{ and } y_2 > 0 \\ \frac{\partial c(u_1,u_2;\rho)}{\partial \beta_2} u_1' u_2' + c(u_1,u_2;\rho) u_1' \frac{\partial u_2'}{\partial \beta_2} & \text{if } y_1 > 0 \text{ and } y_2 > 0 \end{cases}\right),$$

$$= \frac{-1}{\mathcal{L}^2}\frac{\partial \mathcal{L}}{\partial \beta_1}\frac{\partial \mathcal{L}}{\partial \beta_2} + \frac{1}{\mathcal{L}}\begin{cases} 0 & \text{if } y_1 = 0 \text{ and } y_2 = 0 \\ 0 & \text{if } y_1 > 0 \text{ and } y_2 = 0 \\ 0 & \text{if } y_1 = 0 \text{ and } y_2 > 0 \\ \frac{\partial^2 c(u_1,u_2;\rho)}{\partial \beta_1 \partial \beta_2} u_1' u_2' + \frac{\partial c(u_1,u_2;\rho)}{\partial \beta_2}\frac{\partial u_1'}{\partial \beta_1} u_2' + \frac{\partial c(u_1,u_2;\rho)}{\partial \beta_1} u_1' \frac{\partial u_2'}{\partial \beta_2} + c(u_1,u_2;\rho)\frac{\partial u_1'}{\partial \beta_1}\frac{\partial^2 u_2'}{\partial \beta_2^2} & \text{if } y_1 > 0 \text{ and } y_2 > 0 \end{cases}.$$

A.4.7 Mixed derivative with respect to α_1 and α_2

$$\frac{\partial^2 \ell}{\partial \alpha_1 \partial \alpha_2} = \frac{\partial}{\partial \alpha_1}\left(\frac{1}{\mathcal{L}}\frac{\partial \mathcal{L}}{\partial \alpha_2}\right) = \left(\frac{\partial}{\partial \alpha_1}\frac{1}{\mathcal{L}}\right)\frac{\partial \mathcal{L}}{\partial \alpha_2} + \frac{1}{\mathcal{L}}\frac{\partial^2 \mathcal{L}}{\partial \alpha_1 \partial \alpha_2},$$

$$= \frac{-1}{\mathcal{L}^2}\frac{\partial \mathcal{L}}{\partial \alpha_1}\frac{\partial \mathcal{L}}{\partial \alpha_2} + \frac{1}{\mathcal{L}}\left(\frac{\partial}{\partial \alpha_1}\begin{cases} 0 & \text{if } y_1 = 0 \text{ and } y_2 = 0 \\ 0 & \text{if } y_1 > 0 \text{ and } y_2 = 0 \\ \frac{\partial u_2'}{\partial \alpha_2} & \text{if } y_1 = 0 \text{ and } y_2 > 0 \\ \frac{\partial c(u_1,u_2;\rho)}{\partial \alpha_2} u_1' u_2' + c(u_1,u_2;\rho) u_1' \frac{\partial u_2'}{\partial \alpha_2} & \text{if } y_1 > 0 \text{ and } y_2 > 0 \end{cases}\right),$$

$$= \frac{-1}{\mathcal{L}^2}\frac{\partial \mathcal{L}}{\partial \alpha_1}\frac{\partial \mathcal{L}}{\partial \alpha_2} + \frac{1}{\mathcal{L}}\begin{cases} 0 & \text{if } y_1 = 0 \text{ and } y_2 = 0 \\ 0 & \text{if } y_1 > 0 \text{ and } y_2 = 0 \\ 0 & \text{if } y_1 = 0 \text{ and } y_2 > 0 \\ \frac{\partial^2 c(u_1,u_2;\rho)}{\partial \alpha_1 \partial \alpha_2} u_1' u_2' + \frac{\partial c(u_1,u_2;\rho)}{\partial \alpha_2}\frac{\partial u_1'}{\partial \alpha_1} u_2' + \frac{\partial c(u_1,u_2;\rho)}{\partial \alpha_1} u_1' \frac{\partial u_2'}{\partial \alpha_2} + c(u_1,u_2;\rho)\frac{\partial u_1'}{\partial \alpha_1}\frac{\partial^2 u_2'}{\partial \alpha_2^2} & \text{if } y_1 > 0 \text{ and } y_2 > 0 \end{cases}.$$

A.4.8 Mixed derivative with respect to τ and β_1

$$\frac{\partial^2 \ell}{\partial \beta_1 \partial \tau} = \frac{\partial}{\partial \beta_1}\left(\frac{1}{\mathcal{L}}\frac{\partial \mathcal{L}}{\partial \tau}\right) = \left(\frac{\partial}{\partial \beta_1}\frac{1}{\mathcal{L}}\right)\frac{\partial \mathcal{L}}{\partial \tau} + \frac{1}{\mathcal{L}}\frac{\partial^2 \mathcal{L}}{\partial \beta_1 \partial \tau},$$

$$= \frac{-1}{\mathcal{L}^2}\frac{\partial \mathcal{L}}{\partial \beta_1}\frac{\partial \mathcal{L}}{\partial \tau} + \frac{1}{\mathcal{L}}\left(\frac{\partial}{\partial \beta_1}\begin{cases} 0 & \text{if } y_1 = 0 \text{ and } y_2 = 0 \\ 0 & \text{if } y_1 > 0 \text{ and } y_2 = 0 \\ 0 & \text{if } y_1 = 0 \text{ and } y_2 > 0 \\ \frac{\partial c(u_1,u_2;\rho)}{\partial \tau} u_1' u_2' & \text{if } y_1 > 0 \text{ and } y_2 > 0 \end{cases}\right),$$

$$= \frac{-1}{\mathcal{L}^2}\frac{\partial \mathcal{L}}{\partial \beta_1}\frac{\partial \mathcal{L}}{\partial \tau} + \frac{1}{\mathcal{L}}\begin{cases} 0 & \text{if } y_1 = 0 \text{ and } y_2 = 0 \\ 0 & \text{if } y_1 > 0 \text{ and } y_2 = 0 \\ 0 & \text{if } y_1 = 0 \text{ and } y_2 > 0 \\ \frac{\partial^2 c(u_1,u_2;\rho)}{\partial \beta_1 \partial \tau} u_1' u_2' + \frac{\partial c(u_1,u_2;\rho)}{\partial \tau}\frac{\partial u_1'}{\partial \beta_1} u_2' & \text{if } y_1 > 0 \text{ and } y_2 > 0 \end{cases}.$$

A.4.9 Mixed derivative with respect to τ and β_2

$$
\begin{aligned}
\frac{\partial^2 \ell}{\partial \boldsymbol{\beta}_2 \partial \boldsymbol{\tau}} &= \frac{\partial}{\partial \boldsymbol{\beta}_2}\left(\frac{1}{\mathcal{L}}\frac{\partial \mathcal{L}}{\partial \boldsymbol{\tau}}\right) = \left(\frac{\partial}{\partial \boldsymbol{\beta}_2}\frac{1}{\mathcal{L}}\right)\frac{\partial \mathcal{L}}{\partial \boldsymbol{\tau}} + \frac{1}{\mathcal{L}}\frac{\partial^2 \mathcal{L}}{\partial \boldsymbol{\beta}_2 \partial \boldsymbol{\tau}}, \\
&= \frac{-1}{\mathcal{L}^2}\frac{\partial \mathcal{L}}{\partial \boldsymbol{\beta}_2}\frac{\partial \mathcal{L}}{\partial \boldsymbol{\tau}} + \frac{1}{\mathcal{L}}\left(\frac{\partial}{\partial \boldsymbol{\beta}_2}\begin{cases} 0 & \text{if } y_1 = 0 \text{ and } y_2 = 0 \\ 0 & \text{if } y_1 > 0 \text{ and } y_2 = 0 \\ 0 & \text{if } y_1 = 0 \text{ and } y_2 > 0 \\ \frac{\partial c(u_1,u_2;\rho)}{\partial \boldsymbol{\tau}} u_1' u_2' & \text{if } y_1 > 0 \text{ and } y_2 > 0 \end{cases}\right), \\
&= \frac{-1}{\mathcal{L}^2}\frac{\partial \mathcal{L}}{\partial \boldsymbol{\beta}_2}\frac{\partial \mathcal{L}}{\partial \boldsymbol{\tau}} + \frac{1}{\mathcal{L}}\begin{cases} 0 & \text{if } y_1 = 0 \text{ and } y_2 = 0 \\ 0 & \text{if } y_1 > 0 \text{ and } y_2 = 0 \\ 0 & \text{if } y_1 = 0 \text{ and } y_2 > 0 \\ \frac{\partial^2 c(u_1,u_2;\rho)}{\partial \boldsymbol{\beta}_2 \partial \boldsymbol{\tau}} u_1' u_2' + \frac{\partial c(u_1,u_2;\rho)}{\partial \boldsymbol{\tau}} u_1' \frac{\partial u_2'}{\partial \boldsymbol{\beta}_2} & \text{if } y_1 > 0 \text{ and } y_2 > 0 \end{cases}.
\end{aligned}
$$

A.4.10 Mixed derivative with respect to τ and α_1

$$
\begin{aligned}
\frac{\partial^2 \ell}{\partial \boldsymbol{\alpha}_1 \partial \boldsymbol{\tau}} &= \frac{\partial}{\partial \boldsymbol{\alpha}_1}\left(\frac{1}{\mathcal{L}}\frac{\partial \mathcal{L}}{\partial \boldsymbol{\tau}}\right) = \left(\frac{\partial}{\partial \boldsymbol{\alpha}_1}\frac{1}{\mathcal{L}}\right)\frac{\partial \mathcal{L}}{\partial \boldsymbol{\tau}} + \frac{1}{\mathcal{L}}\frac{\partial^2 \mathcal{L}}{\partial \boldsymbol{\alpha}_1 \partial \boldsymbol{\tau}}, \\
&= \frac{-1}{\mathcal{L}^2}\frac{\partial \mathcal{L}}{\partial \boldsymbol{\alpha}_1}\frac{\partial \mathcal{L}}{\partial \boldsymbol{\tau}} + \frac{1}{\mathcal{L}}\left(\frac{\partial}{\partial \boldsymbol{\alpha}_1}\begin{cases} 0 & \text{if } y_1 = 0 \text{ and } y_2 = 0 \\ 0 & \text{if } y_1 > 0 \text{ and } y_2 = 0 \\ 0 & \text{if } y_1 = 0 \text{ and } y_2 > 0 \\ \frac{\partial c(u_1,u_2;\rho)}{\partial \boldsymbol{\tau}} u_1' u_2' & \text{if } y_1 > 0 \text{ and } y_2 > 0 \end{cases}\right), \\
&= \frac{-1}{\mathcal{L}^2}\frac{\partial \mathcal{L}}{\partial \boldsymbol{\alpha}_1}\frac{\partial \mathcal{L}}{\partial \boldsymbol{\tau}} + \frac{1}{\mathcal{L}}\begin{cases} 0 & \text{if } y_1 = 0 \text{ and } y_2 = 0 \\ 0 & \text{if } y_1 > 0 \text{ and } y_2 = 0 \\ 0 & \text{if } y_1 = 0 \text{ and } y_2 > 0 \\ \frac{\partial^2 c(u_1,u_2;\rho)}{\partial \boldsymbol{\alpha}_1 \partial \boldsymbol{\tau}} u_1' u_2' + \frac{\partial c(u_1,u_2;\rho)}{\partial \boldsymbol{\tau}} \frac{\partial u_1'}{\partial \boldsymbol{\alpha}_1} u_2' & \text{if } y_1 > 0 \text{ and } y_2 > 0 \end{cases}.
\end{aligned}
$$

A.4.11 Mixed derivative with respect to τ and α_2

$$
\begin{aligned}
\frac{\partial^2 \ell}{\partial \boldsymbol{\alpha}_2 \partial \boldsymbol{\tau}} &= \frac{\partial}{\partial \boldsymbol{\alpha}_2}\left(\frac{1}{\mathcal{L}}\frac{\partial \mathcal{L}}{\partial \boldsymbol{\tau}}\right) = \left(\frac{\partial}{\partial \boldsymbol{\alpha}_2}\frac{1}{\mathcal{L}}\right)\frac{\partial \mathcal{L}}{\partial \boldsymbol{\tau}} + \frac{1}{\mathcal{L}}\frac{\partial^2 \mathcal{L}}{\partial \boldsymbol{\alpha}_2 \partial \boldsymbol{\tau}}, \\
&= \frac{-1}{\mathcal{L}^2}\frac{\partial \mathcal{L}}{\partial \boldsymbol{\alpha}_2}\frac{\partial \mathcal{L}}{\partial \boldsymbol{\tau}} + \frac{1}{\mathcal{L}}\left(\frac{\partial}{\partial \boldsymbol{\alpha}_2}\begin{cases} 0 & \text{if } y_1 = 0 \text{ and } y_2 = 0 \\ 0 & \text{if } y_1 > 0 \text{ and } y_2 = 0 \\ 0 & \text{if } y_1 = 0 \text{ and } y_2 > 0 \\ \frac{\partial c(u_1,u_2;\rho)}{\partial \boldsymbol{\tau}} u_1' u_2' & \text{if } y_1 > 0 \text{ and } y_2 > 0 \end{cases}\right), \\
&= \frac{-1}{\mathcal{L}^2}\frac{\partial \mathcal{L}}{\partial \boldsymbol{\alpha}_2}\frac{\partial \mathcal{L}}{\partial \boldsymbol{\tau}} + \frac{1}{\mathcal{L}}\begin{cases} 0 & \text{if } y_1 = 0 \text{ and } y_2 = 0 \\ 0 & \text{if } y_1 > 0 \text{ and } y_2 = 0 \\ 0 & \text{if } y_1 = 0 \text{ and } y_2 > 0 \\ \frac{\partial^2 c(u_1,u_2;\rho)}{\partial \boldsymbol{\alpha}_2 \partial \boldsymbol{\tau}} u_1' u_2' + \frac{\partial c(u_1,u_2;\rho)}{\partial \boldsymbol{\tau}} u_1' \frac{\partial u_2'}{\partial \boldsymbol{\alpha}_2} & \text{if } y_1 > 0 \text{ and } y_2 > 0 \end{cases}.
\end{aligned}
$$

A.4.12 Mixed derivative with respect to β_1 and α_1

$$
\begin{aligned}
\frac{\partial^2 \ell}{\partial \boldsymbol{\alpha}_1 \partial \boldsymbol{\beta}_1} &= \frac{\partial}{\partial \boldsymbol{\alpha}_1}\left(\frac{1}{\mathcal{L}}\frac{\partial \mathcal{L}}{\partial \boldsymbol{\beta}_1}\right) = \left(\frac{\partial}{\partial \boldsymbol{\alpha}_1}\frac{1}{\mathcal{L}}\right)\frac{\partial \mathcal{L}}{\partial \boldsymbol{\beta}_1} + \frac{1}{\mathcal{L}}\frac{\partial^2 \mathcal{L}}{\partial \boldsymbol{\alpha}_1 \partial \boldsymbol{\beta}_1}, \\
&= \frac{-1}{\mathcal{L}^2}\frac{\partial \mathcal{L}}{\partial \boldsymbol{\alpha}_1}\frac{\partial \mathcal{L}}{\partial \boldsymbol{\beta}_1} + \frac{1}{\mathcal{L}}\left(\frac{\partial}{\partial \boldsymbol{\alpha}_1}\begin{cases} 0 & \text{if } y_1 = 0 \text{ and } y_2 = 0 \\ \frac{\partial u_1'}{\partial \boldsymbol{\beta}_1} & \text{if } y_1 > 0 \text{ and } y_2 = 0 \\ 0 & \text{if } y_1 = 0 \text{ and } y_2 > 0 \\ \frac{\partial c(u_1,u_2;\rho)}{\partial \boldsymbol{\beta}_1} u_1' u_2' + c(u_1,u_2;\rho)\frac{\partial u_1'}{\partial \boldsymbol{\beta}_1} u_2' & \text{if } y_1 > 0 \text{ and } y_2 > 0 \end{cases}\right), \\
&= \frac{-1}{\mathcal{L}^2}\frac{\partial \mathcal{L}}{\partial \boldsymbol{\alpha}_1}\frac{\partial \mathcal{L}}{\partial \boldsymbol{\beta}_1} + \frac{1}{\mathcal{L}}\begin{cases} 0 & \text{if } y_1 = 0 \text{ and } y_2 = 0 \\ \frac{\partial^2 u_1'}{\partial \boldsymbol{\alpha}_1 \partial \boldsymbol{\beta}_1} & \text{if } y_1 > 0 \text{ and } y_2 = 0 \\ 0 & \text{if } y_1 = 0 \text{ and } y_2 > 0 \\ \frac{\partial^2 c(u_1,u_2;\rho)}{\partial \boldsymbol{\alpha}_1 \partial \boldsymbol{\beta}_1} u_1' u_2' + \frac{\partial c(u_1,u_2;\rho)}{\partial \boldsymbol{\beta}_1}\frac{\partial u_1'}{\partial \boldsymbol{\alpha}_1} u_2' + \frac{\partial c(u_1,u_2;\rho)}{\partial \boldsymbol{\alpha}_1}\frac{\partial u_1'}{\partial \boldsymbol{\beta}_1} u_2' + c(u_1,u_2;\rho)\frac{\partial^2 u_1'}{\partial \boldsymbol{\alpha}_1 \partial \boldsymbol{\beta}_1} u_2' & \text{if } y_1 > 0 \text{ and } y_2 > 0 \end{cases}.
\end{aligned}
$$

A.4.13 Mixed derivative with respect to $\boldsymbol{\beta}_2$ and $\boldsymbol{\alpha}_2$

$$
\begin{aligned}
\frac{\partial^2 \ell}{\partial \boldsymbol{\alpha}_2 \partial \boldsymbol{\beta}_2} &= \frac{\partial}{\partial \boldsymbol{\alpha}_2}\left(\frac{1}{\mathcal{L}}\frac{\partial \mathcal{L}}{\partial \boldsymbol{\beta}_2}\right) = \left(\frac{\partial}{\partial \boldsymbol{\alpha}_2}\frac{1}{\mathcal{L}}\right)\frac{\partial \mathcal{L}}{\partial \boldsymbol{\beta}_2} + \frac{1}{\mathcal{L}}\frac{\partial^2 \mathcal{L}}{\partial \boldsymbol{\alpha}_2 \partial \boldsymbol{\beta}_2}, \\
&= \frac{-1}{\mathcal{L}^2}\frac{\partial \mathcal{L}}{\partial \boldsymbol{\alpha}_2}\frac{\partial \mathcal{L}}{\partial \boldsymbol{\beta}_2} + \frac{1}{\mathcal{L}}\left(\frac{\partial}{\partial \boldsymbol{\alpha}_2}\begin{cases} 0 & \text{if } y_1 = 0 \text{ and } y_2 = 0 \\ 0 & \text{if } y_1 > 0 \text{ and } y_2 = 0 \\ \frac{\partial u_2'}{\partial \boldsymbol{\beta}_2} & \text{if } y_1 = 0 \text{ and } y_2 > 0 \\ \frac{\partial c(u_1,u_2;\rho)}{\partial \boldsymbol{\beta}_2} u_1' u_2' + c(u_1,u_2;\rho) u_1' \frac{\partial u_2'}{\partial \boldsymbol{\beta}_2} & \text{if } y_1 > 0 \text{ and } y_2 > 0 \end{cases}\right), \\
&= \frac{-1}{\mathcal{L}^2}\frac{\partial \mathcal{L}}{\partial \boldsymbol{\alpha}_2}\frac{\partial \mathcal{L}}{\partial \boldsymbol{\beta}_2} + \frac{1}{\mathcal{L}}\begin{cases} 0 & \text{if } y_1 = 0 \text{ and } y_2 = 0 \\ 0 & \text{if } y_1 > 0 \text{ and } y_2 = 0 \\ \frac{\partial^2 u_2'}{\partial \boldsymbol{\alpha}_2 \partial \boldsymbol{\beta}_2} & \text{if } y_1 = 0 \text{ and } y_2 > 0 \\ \frac{\partial^2 c(u_1,u_2;\rho)}{\partial \boldsymbol{\alpha}_2 \partial \boldsymbol{\beta}_2} u_1' u_2' + \frac{\partial c(u_1,u_2;\rho)}{\partial \boldsymbol{\beta}_2} u_1' \frac{\partial u_2'}{\partial \boldsymbol{\alpha}_2} + \frac{\partial c(u_1,u_2;\rho)}{\partial \boldsymbol{\alpha}_2} u_1' \frac{\partial u_2'}{\partial \boldsymbol{\beta}_2} + c(u_1,u_2;\rho) u_1' \frac{\partial^2 u_2'}{\partial \boldsymbol{\alpha}_2 \partial \boldsymbol{\beta}_2} & \text{if } y_1 > 0 \text{ and } y_2 > 0 \end{cases}.
\end{aligned}
$$

A.4.14 Mixed derivative with respect to $\boldsymbol{\beta}_2$ and $\boldsymbol{\alpha}_1$

$$
\begin{aligned}
\frac{\partial^2 \ell}{\partial \boldsymbol{\alpha}_1 \partial \boldsymbol{\beta}_2} &= \frac{\partial}{\partial \boldsymbol{\alpha}_1}\left(\frac{1}{\mathcal{L}}\frac{\partial \mathcal{L}}{\partial \boldsymbol{\beta}_2}\right) = \left(\frac{\partial}{\partial \boldsymbol{\alpha}_1}\frac{1}{\mathcal{L}}\right)\frac{\partial \mathcal{L}}{\partial \boldsymbol{\beta}_2} + \frac{1}{\mathcal{L}}\frac{\partial^2 \mathcal{L}}{\partial \boldsymbol{\alpha}_1 \partial \boldsymbol{\beta}_2}, \\
&= \frac{-1}{\mathcal{L}^2}\frac{\partial \mathcal{L}}{\partial \boldsymbol{\alpha}_1}\frac{\partial \mathcal{L}}{\partial \boldsymbol{\beta}_2} + \frac{1}{\mathcal{L}}\left(\frac{\partial}{\partial \boldsymbol{\alpha}_1}\begin{cases} 0 & \text{if } y_1 = 0 \text{ and } y_2 = 0 \\ 0 & \text{if } y_1 > 0 \text{ and } y_2 = 0 \\ \frac{\partial u_2'}{\partial \boldsymbol{\beta}_2} & \text{if } y_1 = 0 \text{ and } y_2 > 0 \\ \frac{\partial c(u_1,u_2;\rho)}{\partial \boldsymbol{\beta}_2} u_1' u_2' + c(u_1,u_2;\rho) u_1' \frac{\partial u_2'}{\partial \boldsymbol{\beta}_2} & \text{if } y_1 > 0 \text{ and } y_2 > 0 \end{cases}\right), \\
&= \frac{-1}{\mathcal{L}^2}\frac{\partial \mathcal{L}}{\partial \boldsymbol{\alpha}_1}\frac{\partial \mathcal{L}}{\partial \boldsymbol{\beta}_2} + \frac{1}{\mathcal{L}}\begin{cases} 0 & \text{if } y_1 = 0 \text{ and } y_2 = 0 \\ 0 & \text{if } y_1 > 0 \text{ and } y_2 = 0 \\ \frac{\partial u_2'}{\partial \boldsymbol{\beta}_2} & \text{if } y_1 = 0 \text{ and } y_2 > 0 \\ \frac{\partial^2 c(u_1,u_2;\rho)}{\partial \boldsymbol{\alpha}_1 \partial \boldsymbol{\beta}_2} u_1' u_2' + \frac{\partial c(u_1,u_2;\rho)}{\partial \boldsymbol{\beta}_2} \frac{\partial u_1'}{\partial \boldsymbol{\alpha}_1} u_2' + \frac{\partial c(u_1,u_2;\rho)}{\partial \boldsymbol{\alpha}_1} u_1' \frac{\partial u_2'}{\partial \boldsymbol{\beta}_2} + c(u_1,u_2;\rho) \frac{\partial u_1'}{\partial \boldsymbol{\alpha}_1} \frac{\partial u_2'}{\partial \boldsymbol{\beta}_2} & \text{if } y_1 > 0 \text{ and } y_2 > 0 \end{cases}.
\end{aligned}
$$

A.4.15 Mixed derivative with respect to $\boldsymbol{\beta}_1$ and $\boldsymbol{\alpha}_2$

$$
\begin{aligned}
\frac{\partial^2 \ell}{\partial \boldsymbol{\alpha}_2 \partial \boldsymbol{\beta}_1} &= \frac{\partial}{\partial \boldsymbol{\alpha}_2}\left(\frac{1}{\mathcal{L}}\frac{\partial \mathcal{L}}{\partial \boldsymbol{\beta}_1}\right) = \left(\frac{\partial}{\partial \boldsymbol{\alpha}_2}\frac{1}{\mathcal{L}}\right)\frac{\partial \mathcal{L}}{\partial \boldsymbol{\beta}_1} + \frac{1}{\mathcal{L}}\frac{\partial^2 \mathcal{L}}{\partial \boldsymbol{\alpha}_2 \partial \boldsymbol{\beta}_1}, \\
&= \frac{-1}{\mathcal{L}^2}\frac{\partial \mathcal{L}}{\partial \boldsymbol{\alpha}_2}\frac{\partial \mathcal{L}}{\partial \boldsymbol{\beta}_1} + \frac{1}{\mathcal{L}}\left(\frac{\partial}{\partial \boldsymbol{\alpha}_2}\begin{cases} 0 & \text{if } y_1 = 0 \text{ and } y_2 = 0 \\ \frac{\partial u_1'}{\partial \boldsymbol{\beta}_1} & \text{if } y_1 > 0 \text{ and } y_2 = 0 \\ 0 & \text{if } y_1 = 0 \text{ and } y_2 > 0 \\ \frac{\partial c(u_1,u_2;\rho)}{\partial \boldsymbol{\beta}_1} u_1' u_2' + c(u_1,u_2;\rho) \frac{\partial u_1'}{\partial \boldsymbol{\beta}_1} u_2' & \text{if } y_1 > 0 \text{ and } y_2 > 0 \end{cases}\right), \\
&= \frac{-1}{\mathcal{L}^2}\frac{\partial \mathcal{L}}{\partial \boldsymbol{\alpha}_2}\frac{\partial \mathcal{L}}{\partial \boldsymbol{\beta}_1} + \frac{1}{\mathcal{L}}\begin{cases} 0 & \text{if } y_1 = 0 \text{ and } y_2 = 0 \\ 0 & \text{if } y_1 > 0 \text{ and } y_2 = 0 \\ 0 & \text{if } y_1 = 0 \text{ and } y_2 > 0 \\ \frac{\partial^2 c(u_1,u_2;\rho)}{\partial \boldsymbol{\alpha}_2 \partial \boldsymbol{\beta}_1} u_1' u_2' + \frac{\partial c(u_1,u_2;\rho)}{\partial \boldsymbol{\beta}_1} u_1' \frac{\partial u_2'}{\partial \boldsymbol{\alpha}_2} + \frac{\partial c(u_1,u_2;\rho)}{\partial \boldsymbol{\alpha}_2} \frac{\partial u_1'}{\partial \boldsymbol{\beta}_1} u_2' + c(u_1,u_2;\rho) \frac{\partial u_1'}{\partial \boldsymbol{\beta}_1} \frac{\partial u_2'}{\partial \boldsymbol{\alpha}_2} & \text{if } y_1 > 0 \text{ and } y_2 > 0 \end{cases}.
\end{aligned}
$$

B Additional Figures and Tables

B.1 Additional Simulation Plots

This section contains the plots of CV σ and zero-inflation rates p in Scenario II Simulation shown in Figure 1. The plots of CV σ in Scenario III Simulation are shown in Figure 2.

B.2 Significant Gene Pair Plots

This section contains the remaining significant 39 gene pairs correlation plots shown in Figure 3.

B.3 QQ Plots

This section shows the QQ plots of 4 gene pairs model fitting in Section 3.

B.4 Zero-inflation Plots

Figure 5 shows more examples of zero-inflation rate (P_0) changes from important genes.

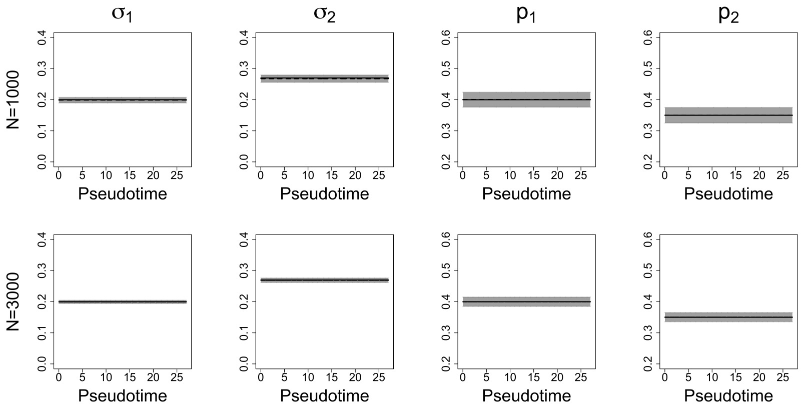

Fig. 1: σ and p plots in Scenario II simulation: The solid lines are the true smooth functions, and the dashed lines are the mean estimates for 1,000 iterations. The shaded areas are point-wise ranges from 5% to 95% quantile. The numbers of observations are 1,000 and 3,000 for each row.

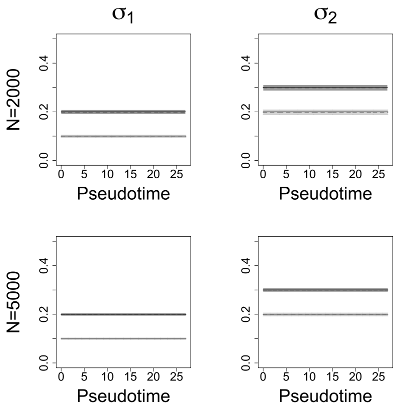

Fig. 2: σ plots in Scenario III simulation: The light gray color is for the control group, and the dark gray color is for the mutant group. The solid lines are the true smooth functions, and the dashed lines are the mean estimates for 1,000 iterations. The shaded areas are point-wise ranges from 5% to 95% quantile. The numbers of observations are 2,000 and 5,000 for each row.

Table 1. WNT signaling pathway and TF gene lists used in Experiment Data Analysis.

	**Gene**
WNT pathway	*Apc, Btrc, Cacybp, Camk2d, Cby1, Ccnd2, Ccnd3, Chd8, Crebbp, Csnk1a1, Csnk1e Csnk2a1, Csnk2a2, Csnk2b, Ctbp1, Ctnnb1, Ctnnbip1, Ctnnd2, Cul1, Cxxc4, Ep300 Fbxw11, Fzd2, Fzd3, Gsk3b, Jun, Lef1, Lgr4, Lrp6, Nlk, Plcb4, Ppp3ca, Ppp3cb Ppp3r1, Prickle1, Prkaca, Prkacb, Rac1, Rbx1, Rhoa, Rock2, Ruvbl1, Ryk, Senp2 Senp2, Siah1a, Smad4, Tbl1x, Tcf7l2, Tle1, Tle4*
TF	*Aldh1a2, Axin2, Bmp2, Bmp4, Cga, Egr1, Fgf1, Fgf10, Fgf8,Gata2, Gli1, Gli2, Gli3 Hesx1, Isl1, Lef1, Lhx2, Lhx3, Lhx4, Mki67, Msx1, Neurod1, Neurod4, Nkx2-1, Nkx2-4 Notch2, Otx2, Pax6, Pitx1, Pitx2, Pomc, Pou1f1, Prl, Prop1, Robo2, Rxrg, Sf1, Shh Shh, Six3, Sox1, Sox2, Sox3, Tbx19, Tcf7l2, Tef, Tshb, Wnt11, Wnt3, Wnt4, Wnt5a Wnt5b, Wnt6, Wnt7b, Wnt8b, Wnt9a*

B.5 Data Analysis Gene List

Table 1 provides the two gene lists we used in Experiment Data Analysis.

References

R. A. Rigby, M. D. Stasinopoulos, G. Z. Heller, and F. D. Bastiani. *Distributions for modeling location, scale, and shape: Using GAMLSS in R.* Chapman and Hall/CRC, 2019.

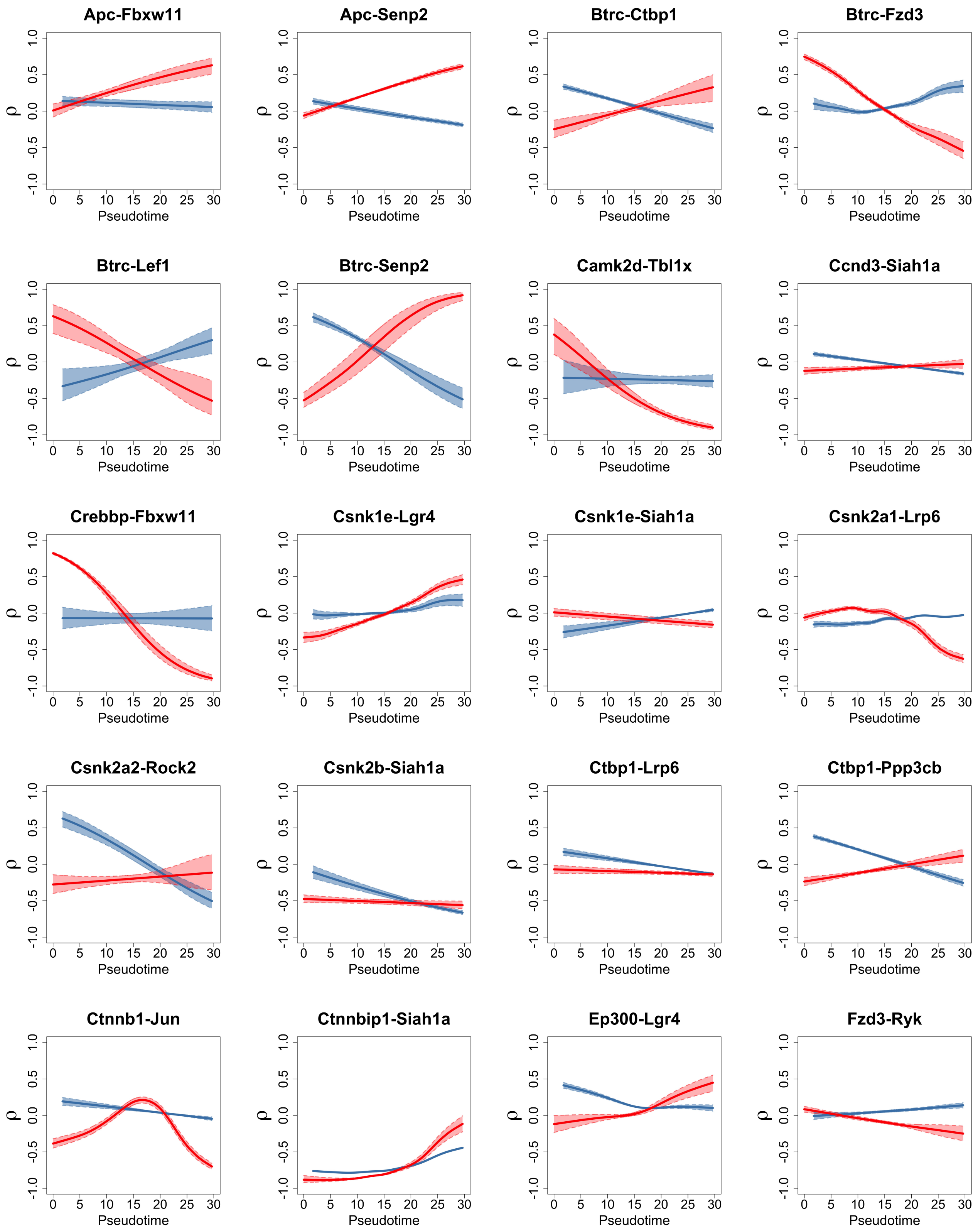

Fig. 3: Significant gene pairs with adjusted p-values less than 0.05. The blue solid curve is the fitted line for the control group, and the blue dashed line is 95% CI of the fit. The red solid curve is the fitted line for the mutant group, and the red dashed line is 95% CI of the fit.

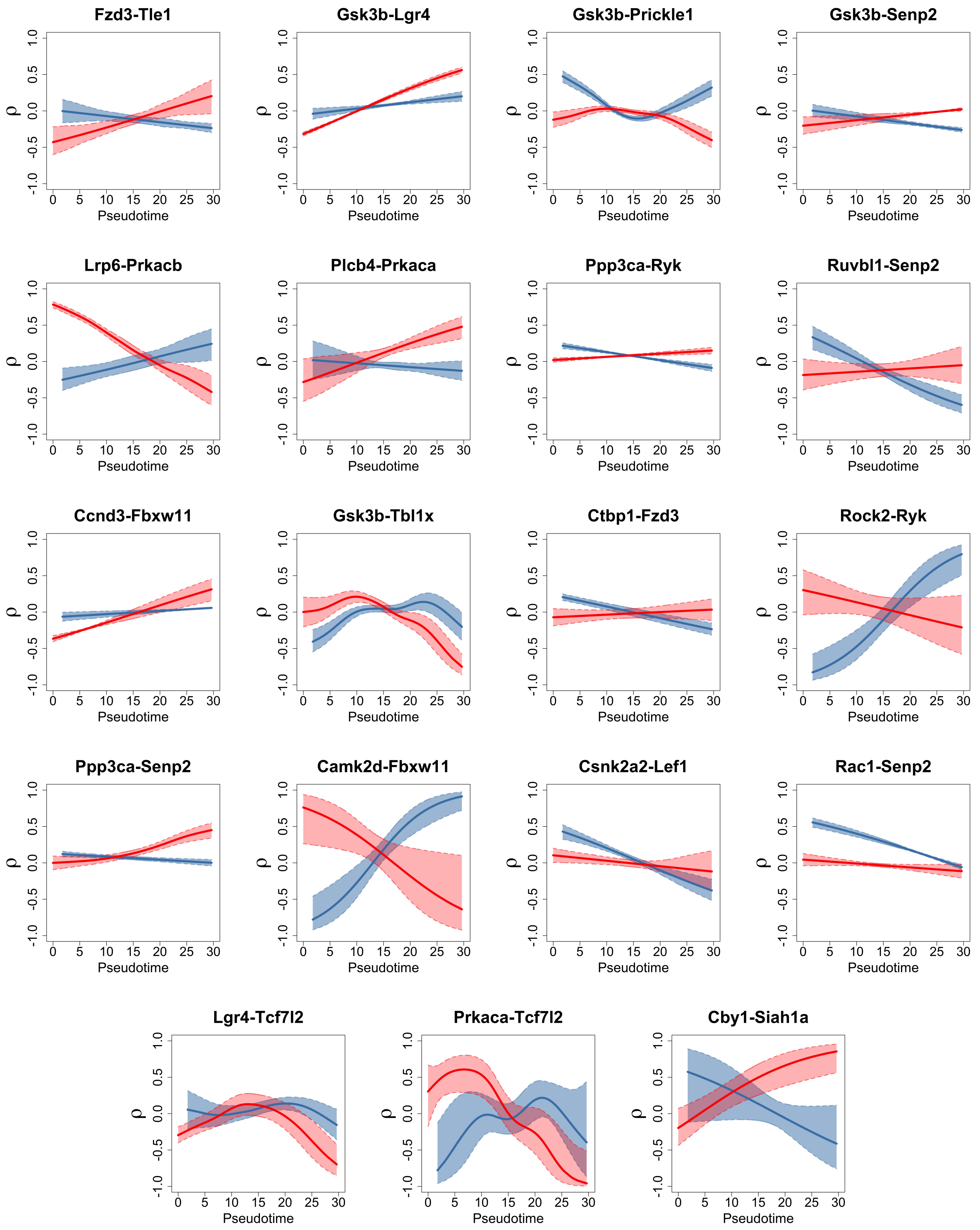

Fig. 3: Figure 3 (continued)

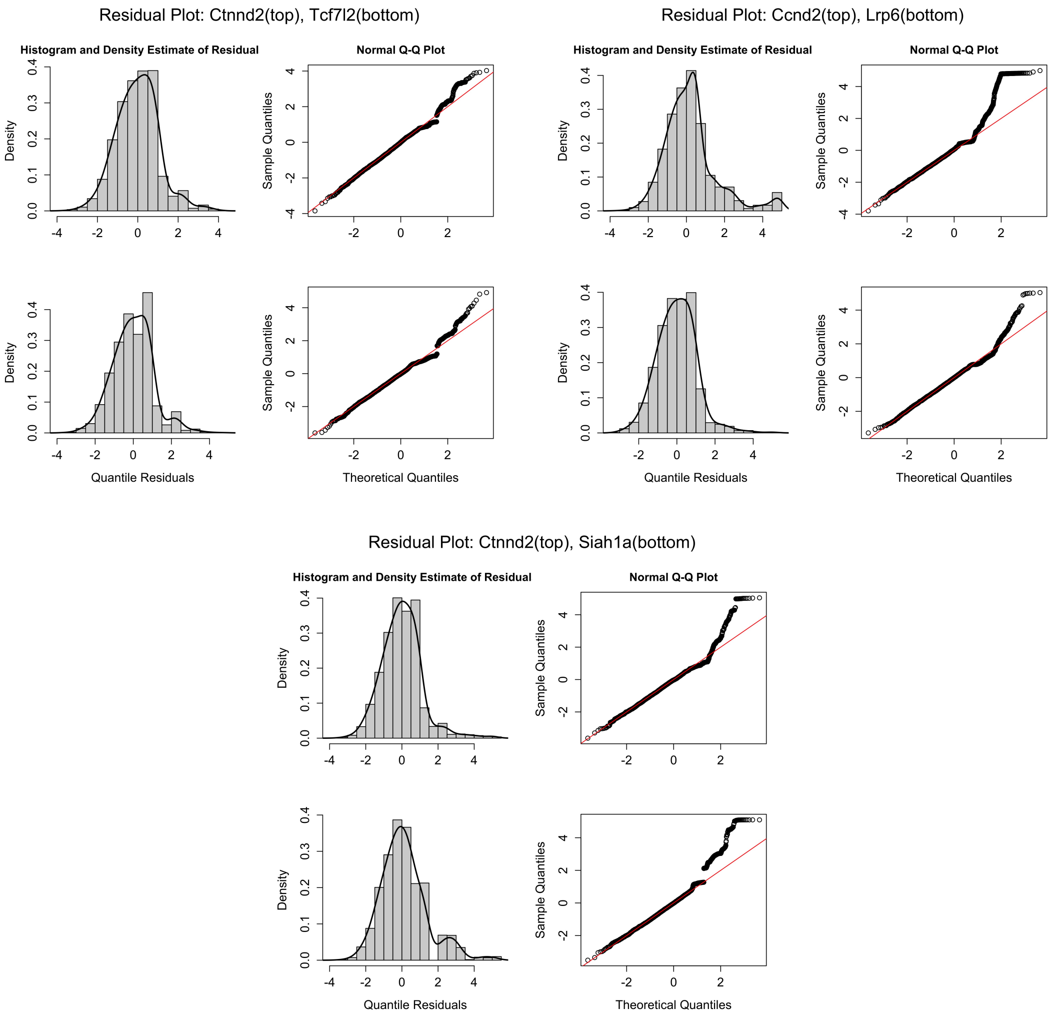

Fig. 4: Residual plots of normalized quantile for 3 example gene pairs. The red solid line is $y = x$.

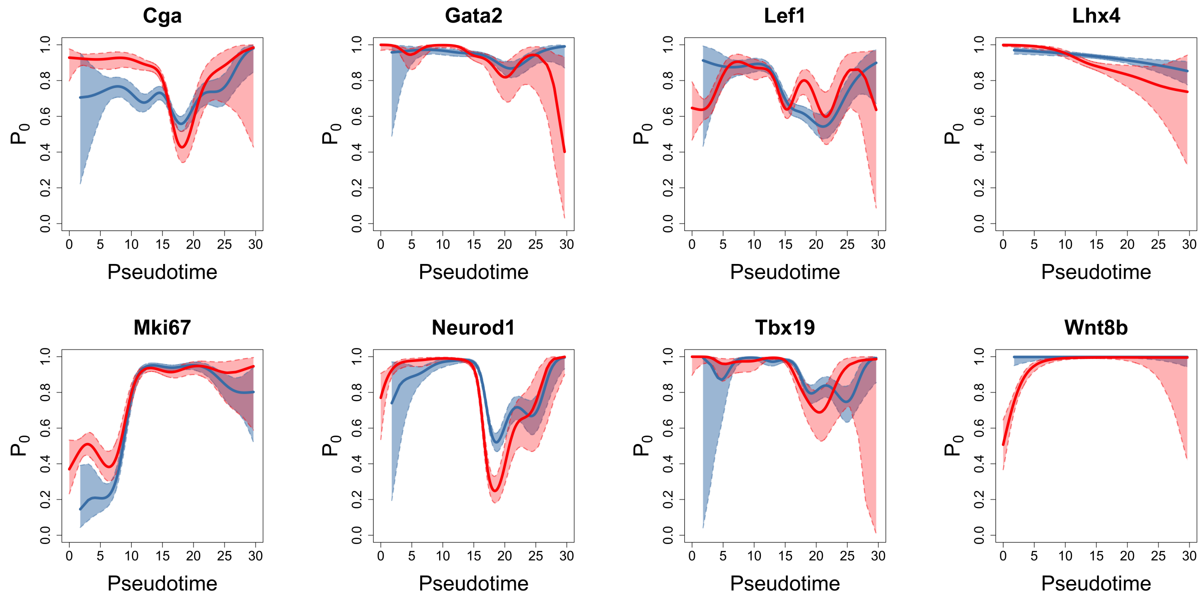

Fig. 5: More examples of zero-inflation rate (P_0) changes from important genes. The blue solid curve is the fitted line for the wild-type group, and the blue dashed line is 95% CI of the fit. The red solid curve is the fitted line for the mutant group, and the red dashed line is 95% CI of the fit. .
